# Quantum Dots Compete at the Acme of MXene Family for the Optimal Catalysis

**DOI:** 10.1007/s40820-022-00908-3

**Published:** 2022-08-02

**Authors:** Yuhua Liu, Wei Zhang, Weitao Zheng

**Affiliations:** Key Laboratory of Automobile Materials MOE, and School of Materials Science and Engineering, and Jilin Provincial International Cooperation Key Laboratory of High-Efficiency Clean Energy Materials, and Electron Microscopy Center, and International Center of Future Science, Jilin University, Changchun, 130012 People’s Republic of China

**Keywords:** MXene, Quantum dots, Catalysis, Surface groups, Structure

## Abstract

It is well known that two-dimensional (2D) MXene-derived quantum dots (MQDs) inherit the excellent physicochemical properties of the parental MXenes, as a Chinese proverb says*, “Indigo blue is extracted from the indigo plant, but is bluer than the plant it comes from.”* Therefore, 0D QDs harvest larger surface-to-volume ratio, outstanding optical properties, and vigorous quantum confinement effect. Currently, MQDs trigger enormous research enthusiasm as an emerging star of functional materials applied to physics, chemistry, biology, energy conversion, and storage. Since the surface properties of small-sized MQDs include the type of surface functional groups, the functionalized surface directly determines their performance. As the Nobel Laureate Wolfgang Pauli says, *“God made the bulk, but the surface was invented by the devil*,*”* and it is just on the basis of the abundant surface functional groups, there is lots of space to be thereof excavated from MQDs. We are witnessing such excellence and even more promising to be expected. Nowadays, MQDs have been widely applied to catalysis, whereas the related reviews are rarely reported. Herein, we provide a state-of-the-art overview of MQDs in catalysis over the past five years, ranging from the origin and development of MQDs, synthetic routes of MQDs, and functionalized MQDs to advanced characterization techniques. To explore the diversity of catalytic application and perspectives of MQDs, our review will stimulate more efforts toward the synthesis of optimal MQDs and thereof designing high-performance MQDs-based catalysts.

## Highlights

- All the synthesis routes and surfaced-modified strategy of MXene-derived quantum dots (MQDs), the synthesis of MQDs-based nanocomposites, and advanced characterization techniques of MQDs are fully covered.
- Catalytic application is classified and discussed by judging the roles of MQDs.
- Current challenge and prospect are proposed for promoting the development and catalytic application of MQDs.

## Graphical abstract

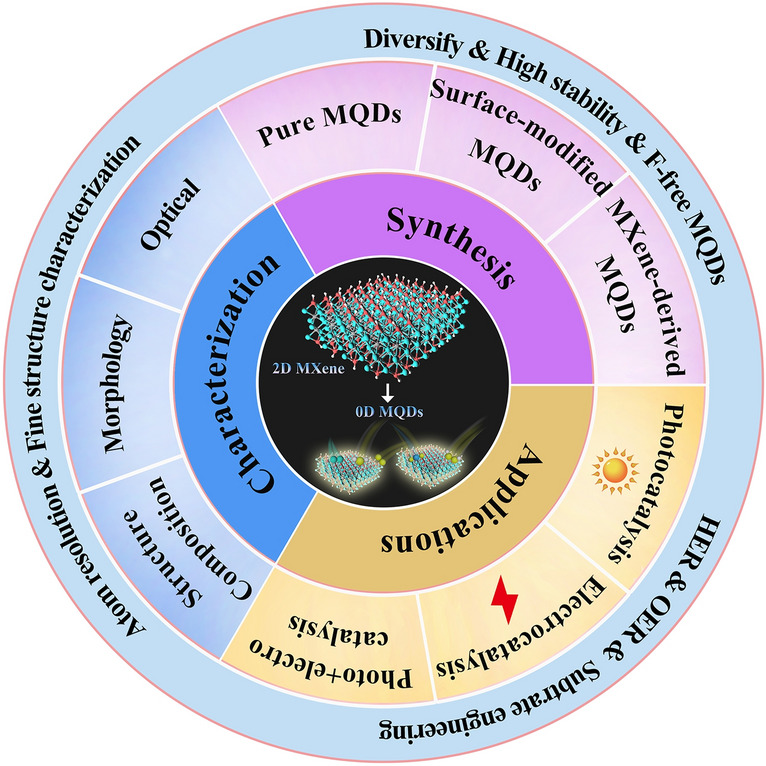

## Introduction

**Fig. 1:**
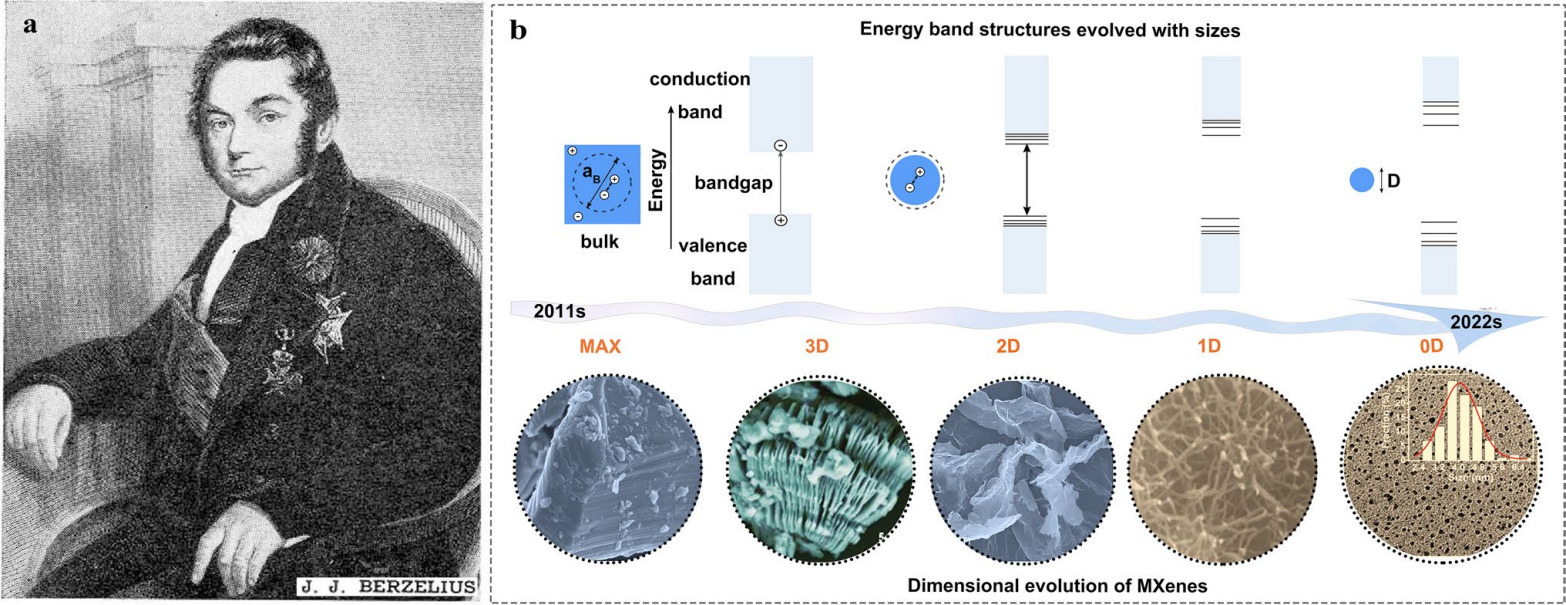
**a** Jöns Jacob Berzelius (1779–1848) [1]. Copyright @1948, American Chemical Society. **b** Schematic illustration of the energy band structure of materials with different sizes, and typical images of MAX and MXene from 3D multilayer to 2D nanosheets to 1D nanowires to 0D nanodots [6]. Copyright @2021, The American Association for the Advancement of Science. The morphology was obtained by field emission scanning electron microscopy [7–10], Copyright @2011, WILEY–VCH, @2019, WILEY–VCH, @2018, Elsevier and @2018, American Chemical Society

In 1836, the terms of catalyst and catalysis were firstly defined by the Swedish scientist Jöns Jakob Berzelius (Fig. 1a), describing “the catalyst was a new matter that can produce chemical activity,” and Pt catalyzing the conversion of ethanol to acetic acid witnessed this magical discovery [1, 2]. Thereafter, considerable various catalytic reactions were applied to an industrial production, largely promoting the development of chemistry and human society. Until 1910 [3], synthesis of ammonia promotes the development of agricultural cultivation, fuel production, and industrial manufacture, and thereof becomes a landmark in the history and development of catalytic technologies [4, 5]. Furthermore, converting water, carbon monoxide (CO), and carbon dioxide (CO_2_) electrocatalytically to clean fuels (e.g*.*, H_2_, CH_3_OH) for replacing the limited fossil fuels is an important production route to confront the energy crisis. Currently, the catalytic reactions are mainly classified into electrocatalysis, photocatalysis, and photoelectrochemical reaction in dependence of various external energy devices.

Nowadays, the precious metal catalysts are crucial, e.g., Pt-based [11, 12], ruthenium oxide (RuO_2_) [13], and iridium oxide (IrO_2_) nanomaterials [14, 15] are listed as the most effective catalysts for driving CO oxidation, hydrogen evolution reaction (HER), NH_3_ synthesis, and oxygen evolution reaction (OER). However, their high-cost and limited reserves hinder a large-scale utilization in industry. Therefore, many researchers focus on reducing the contents of noble-metal (*e.g.*, Au, Pt, Ru, and Pd) loading on a support [16–18], or decreasing size for increasing amounts of active ingredients (single atoms, nanoclusters, and quantum dots (QDs)) [19–21], or coupling noble-metal with non-metal to regulate electron structure for preparing highly active catalysts (RhB, Pt_3_Ni, and Pd_2_B) [22–24], or searching for the replaceable non-precious metals (e.g., transition metal (TM) Fe, Co, and Ni) and their alloys [25, 26]. Presently, TM oxides (MoO, MnO_2_, Co_3_O_4_) [27, 28], Co, Ni, and Cu binary oxides (NiO/CuO, Co_3_O_4_/NiO, and CuO/Co_3_O_4_) [29, 30], and layered double hydroxide systems (FeNi-LDH, NiCo-LDH, and CoAl-LDH) have been widely investigated in the catalytic fields [31, 32]. Particularly, some TM-based catalysts (Bi, Cu, Mo, Cr, and W) have made great progress [33]. Furthermore, TM and non-metallic single atoms, carbon-based hybrid, perovskite, and MOF-derived nanomaterials become also the research hotspots of catalytic fields. Therefore, it is witnessing the flourishing landscape to long-term explore low-cost, highly effective, and durable catalysts.

In 2004, the exfoliation for graphene opened the door of low dimensional materials, trigging great enthusiasm for exploring a wide range of 2D layered materials, such as graphitic carbon nitride (g-C_3_N_4_) [34, 35], hexagonal boron nitride (h-BN) [36, 37], transition metal dichalcogenides (TMDs) and transition metal oxides (TMOs) [38, 39]. Among a variety of 2D materials, graphene holds the highest flexibility, conductivity (10^6^ S cm^−2^), and transmittance (97.7%) so far [40]. In 2011, the 3D bulk Ti_3_AlC_2_ was immersed in hydrofluoric (HF) acid solution by Gogotsi group. As a result, Ti_3_C_2_T_x_ (T represents functional groups such as hydroxyl (–OH), oxygen (–O), fluorine (–F) or chlorine (–Cl), and x is the contents of groups) was stripped, called as MXene with a layered structure similar to graphene, and the excellent conductivity (6000–8000 S cm^−2^) well comparable to graphene [41–43]. As a new 2D layered material, MXenes have the merits of other 2D materials; more importantly, surface functionalization renders them easily achieve the improved properties. Therefore, the application covers biomedical [44], energy storage devices (battery, supercapacitor) [45, 46], sensors [47], catalysis [48], and electromagnetic interference shielding [49].

Usually, MXenes can be prepared by selecting removal of “A” layers of MAX phases or with the similar compositions, and the forces include either mechanical or chemical exfoliations [50]. However, the wet-chemical etching method is most of the facile and high-production yield processes. Bulk MAX is referred to as a hexagonal layered ternary transition metal carbide, nitride, or carbonitride, where M is an early transition metal, A is a group IIIA or IVA element, X is C or/and N, which can be described by the formula as M_n+1_AX_n_ (n = 1, 2 or 3). The stronger M–X bond is a mixture of covalent, metallic, and ionic ones, but the M–A metallic bond is weaker. Therefore, the M_n+1_X_n_T_x_ is usually prepared by etching “A” layer of the specific solvent, such as HF, NH_4_HF, and HCl/LiF [51].

**Fig. 2:**
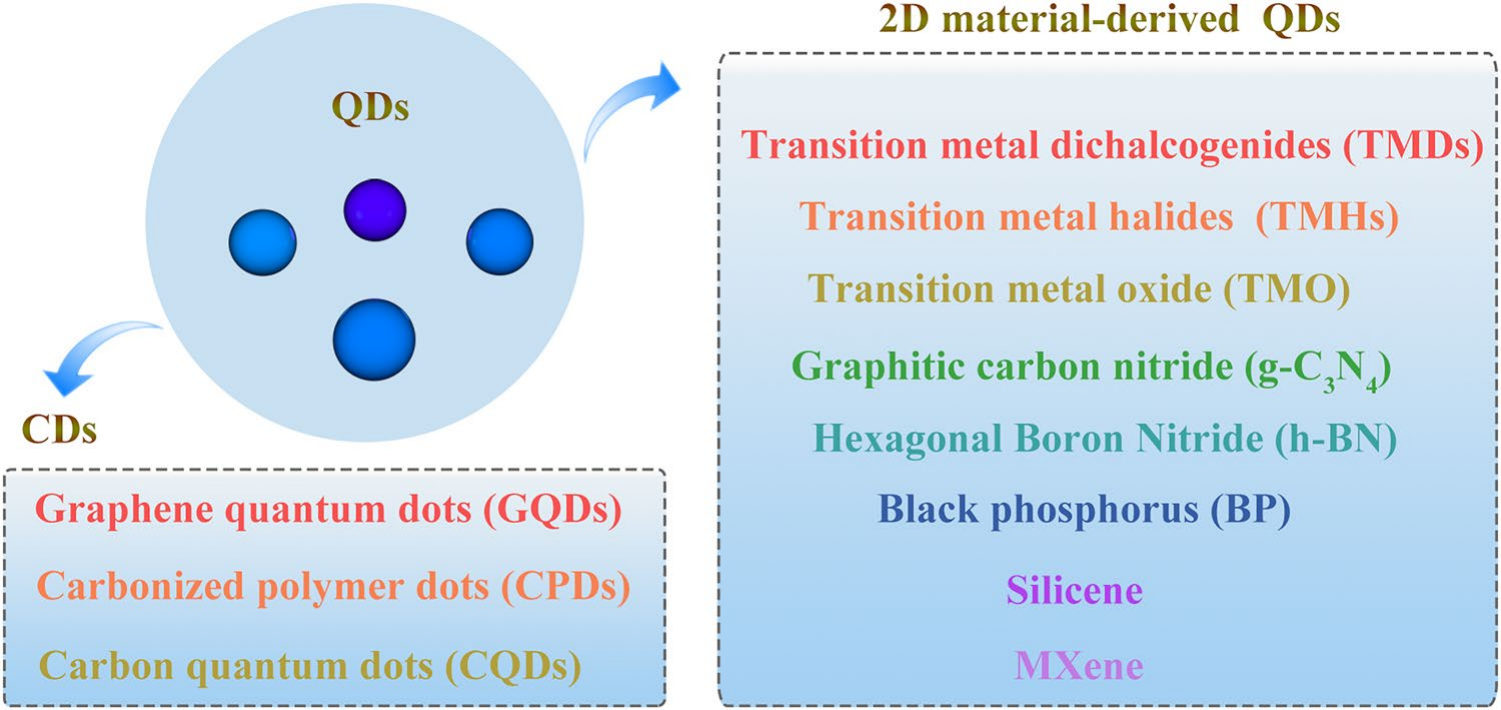
The classification of quantum dots

Moreover, preparing fluorine-free (F-free) MXenes has attracted serious concern for meeting the requirements of specific functions and avoiding the corrosive reagents. [52–54] After etching, MXenes nanosheets were obtained by using intercalation agent such as dimethyl sulfoxide (DMSO), tetrabutylammonium hydroxide (TBAOH), tetramethylammonium hydroxide (TMAOH), alcohols, choline hydroxide, or n-butylamine through centrifugation or sonication method. However, when the lateral size of MXenes nanosheets is further reduced to nanometer size that is smaller than Bohr radius of the exciton (lateral size < 10 nm), shows strong photoluminescence, called zero-dimensional semiconductor nanomaterials—MXenes QDs (MQDs), which is an emerging branch of QDs (Fig. 2) [55]. In 2017 [56], Wang et al. firstly reported the Ti_3_C_2_ fluorescent ultrasmall monolayers MXene sheets by concurrent intralayer cutting and interlayer delamination. The method was also extended to prepare Ti_2_C and Nb_2_C ultrasmall sheets. The Ti_3_C_2_ ultrasmall sheet has the lateral dimension of 2–8 nm and the average thickness of 1 nm. Furthermore, similar to the carbon dots, Ti_3_C_2_ monolayers sheets showed the strong and tunable photoluminescence and excitation-dependent behavior with the change of pH. Although they were not defined as Ti_3_C_2_ QDs, both their size and the fluorescence behavior are typical of QDs. MXene-derived MQDs not only inherit the merits of low toxicity, heavy metal-free ones, natural hydrophilicity, metallic conductivity, flexibility, and abundant active catalytic sites of the parental MXene [40, 57], but also afford the characteristics likes other QDs, such as best dispersibility, unique photoluminescence (PL) properties, quantum confinement effects, and small-size effects. Such diverse properties expand their applications for energy storage, catalysis, optoelectronic device, environmental monitoring, biomedical, and sensors.

**Fig. 3:**
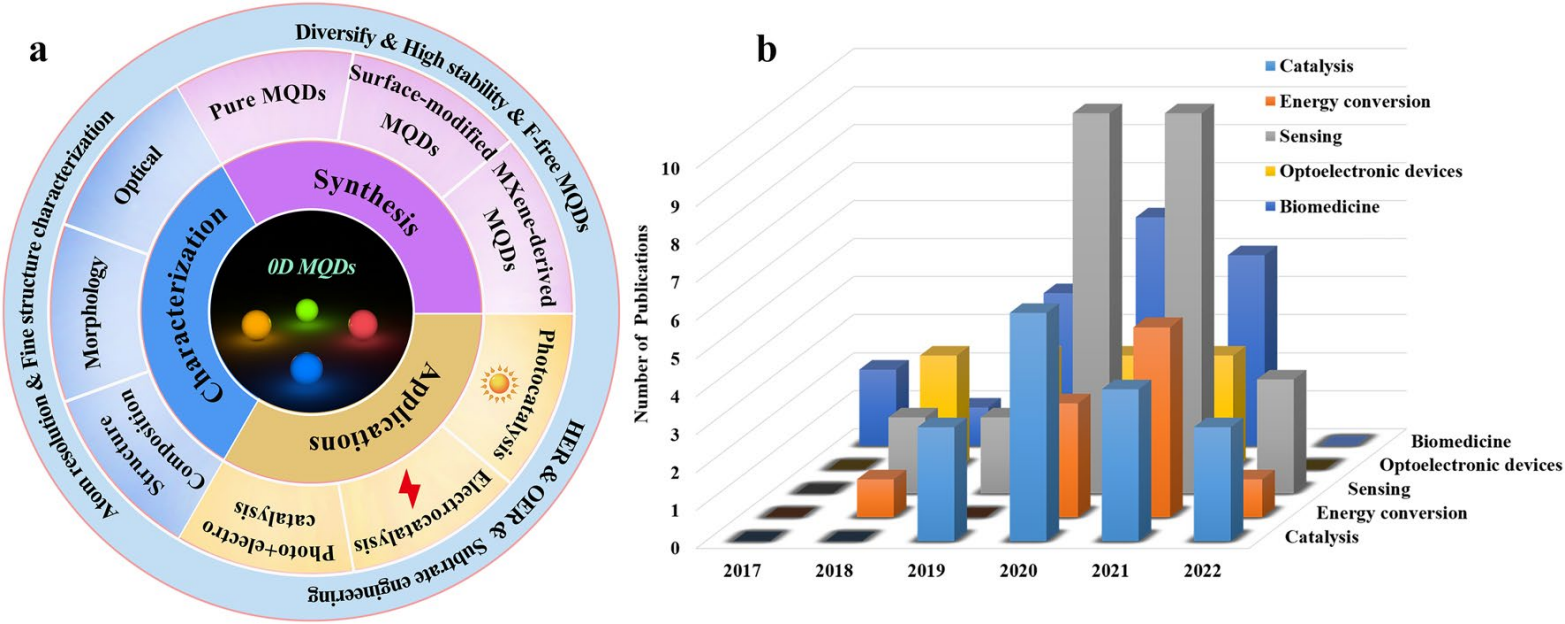
**a** State of the art and prospect of 0D MQDs. **b** Number of journal publications related to publication time and applications science 2017 (Source: Web of Science)

To date, the publications of MQDs have been increasing dramatically, and the focused fields include optoelectronic device, sensors, catalysis, energy storage, and biomedical applications. In this review, we highlight systematically the research status of MQDs on catalysis, rather than covering all the respects of other promising application. Also, the research progress of MQDs, ranging from their synthesis and modification to advanced characterization techniques are summarized. Finally, their perspectives in catalytic field are discussed briefly (Fig. 3a). Expectedly, we hope this review will contribute to guide a rational design of high-performance MQDs-based catalysts in catalytic applications.

## Development of MQDs

Since the MXene was discovered in 2011 by Gogotsi [7], achieving the soaring development from 3D layered bulk materials to 2D nanosheets, 1D nanowires, 0D QDs. Meanwhile, physical and chemical properties of materials change with the decrease in the lateral size, which can effectively enrich the surface areas, increase amounts of active sites that are particularly relevant to the homogeneous–heterogeneous catalysis. Especially, as the size of materials is reduced and smaller than its exciton Bohr radius, they show strong photoluminescence, endowing small-size effect and quantum confinement effect (Fig. 1b). However, the shortcoming is obvious since the required harsh synthesis condition and accompanied high surface energy leads to easy agglomeration. The photoluminescent Ti_3_C_2_ MQDs first synthesized by a facile hydrothermal method for imaging in 2017. The average size of MQDs can be regulated by controlling the reaction temperature. However, the product is strictly dependent on the reaction conditions, when the reaction temperature surpasses 100 °C, the hybrid structures are obtained and even phase transition may occur [58, 59]. Afterward, some researches involve in synthesis of the MQDs, but an increasing number of efforts were devoted toward preparing the multifunctional MQDs. We summarized the number of publications and application fields about MQDs. As described in Fig. 3b, over the past five years, the number of articles about MQDs has an obvious increase, and the application covers catalysis, energy storage, sensors, biomedical, and optoelectronic devices, especially in sensors, catalysis, and biomedical applications. Until now, the research enthusiasm of MQDs continues growing, and the application in the field of catalysis gradually becomes a hot topic.

**Fig. 4:**
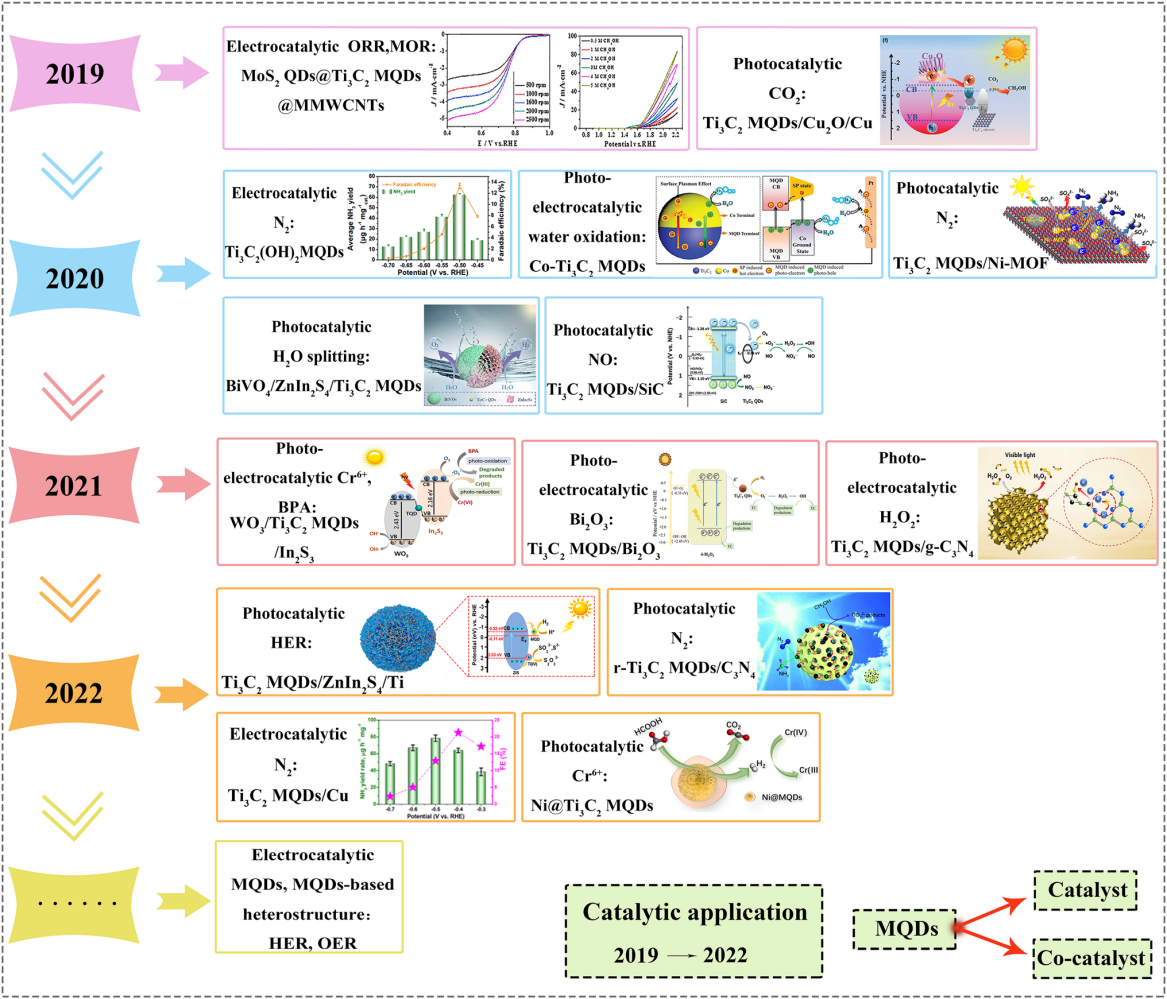
The timeline showing the development of MQDs in catalysis in the past few years. Reproduced with permission from Refs. [66–79]. Copyright @2019, Elsevier, @2019, WILEY–VCH, @2020, Elsevier, @2020, American Chemcial Society, @2020, WILEY–VCH, @2020, American Chemical Society, @2020, WILEY–VCH, @2021, American Chemical Society, @2021, Elsevier, @2021, Elsevier, @2022, The Royal Society of Chemistry, @2022, Elsevier, @2022. MDPI and the author, @2022, Zhengzhou University and Wiley

Timeline recording the development of MQDs is listed for the fields of photocatalysis, electrocatalysis, and photoelectrochemical (Fig. 4). However, other applications such as electrocatalytic hydrogen evolution reaction (HER) and oxygen evolution reaction (OER) require to be mechanistically explored, and various catalysts of MQDs such as Ti_2_C, V_2_N, and Mo_2_C worth to be thereof prepared. For instance, the HER activities of 2D Ti_2_C, Ti_3_C_2_, Nb_2_C, Nb_3_C_4_, and V_2_C MXene with O* or/and OH* terminals calculated by using density functional theory [60]. Their result shows that Ti_2_CO_2_ MXene has optimized Gibbs free energy of hydrogen adsorption (*ΔG*_H*_), which is regarded as an ideal candidate of electrocatalysts. Furthermore, some literatures report that Ti_2_CT_x_ MXene catalyst has achieved excellent HER performance under acidic conditions, whereas 570 mV@-10 mA cm^−2^ was afforded for the HER activity affords under alkaline conditions [54, 61, 62]. Additionally, the excellent HER activity of MQDs is also beneficial to achieve outstanding catalytic dehydrogenation ability, as most studies show that the strong H adsorption ability of MXene leads to easier dehydrogenation [63–65]. Anyhow, the ongoing efforts are needed toward the ultimate ideal commercial alkaline water splitting electrocatalyst. Thus, it is very necessary to optimize the performance of Ti_2_C MXene catalyst under alkaline conditions, and exploring the HER performance of 0D Ti_2_C MQDs or other analogues is worthy for pondering in the future.

## Preparation of MQDs

Over the past decade, various methods have been adopted to synthesize two-dimensional inorganic derivatives such as graphene [80], phosphorene [81], 2D layered carbides-based QDs [82], and TMOs-based QDs [83]. Due to the similar layer structures (the strong covalent or ionic bonds in layers, the weak van der Waals forces in interlayers), the synthesis of 2D MXene-derived QDs is quite similar to other inorganic QDs. Up to now, there are different types of MQDs that have been prepared by top-down synthesis methods, such as Ti_3_C_2_ QDs, V_2_C QDs, Nb_2_C QDs, Ti_2_N QDs, TiCN QDs, MXene-derived Ti_n_O_2n-1_ and TiO_2_/C-QDs [84–90]. Furthermore, various surface modifications were used to improve the properties for further catalytic applications. A summary on the synthesis is classified into pure MQDs, and MQDs with surface modifications in the following.

### Synthesis of Pure MQDs

**Fig. 5:**
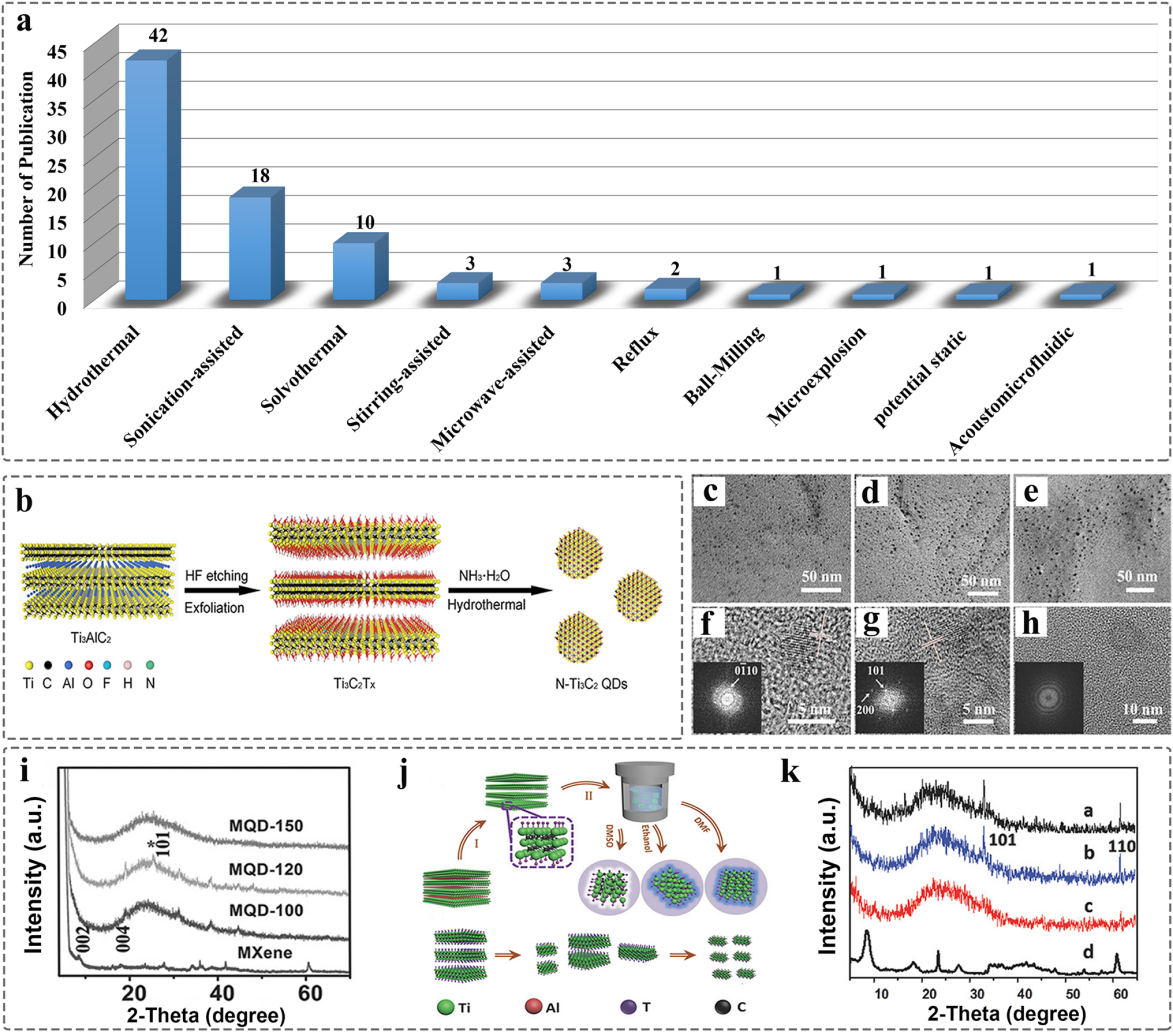
Development of synthesis methods and synthesis of MQDs.** a** Number of synthesis methods publications on MQDs. (Source: Web of science, 2017 to 2022s). **b** Scheme of hydrothermal synthesis method [107]. Copyright @2021, American Chemical Society. **c-h** Morphology of MQDs at different reaction temperature of 100, 120, and 150 °C. The data was obtained by transmission electron microscopy; **i** XRD characterization of MQDs [58]. Copyright @2017, WILEY–VCH. **j** Scheme of synthesis MQDs at different solvents of DMSO, DMF, and ethanol; **k** XRD characterization of MQDs [96]. Copyright @2018, WILEY–VCH

Generally, the synthesis of MQDs consists of two steps, including the chemical exfoliations of 2D MXenes from 3D bulk MAX for the first step, or the homogeneous 2D MXene can be obtained by bottom-up route such as chemical vapor deposition (CVD) growth [91, 92]. The next step is the preparation of MQDs mainly by top-down methods. Figure 5a shows all the synthesis methods in the current reports. Hydrothermal method with low energy consumption is regarded as the most common approach (Fig. 5b), and thereof MQDs have the advantages of morphology, size control, high crystallinity, high yield, etc. In the process, the formation mechanism assisted with high temperature and pressure enables 2D MXenes for easy cracking and assembling. The base or acid as medium with the controlled pH value 6–9, which reacts with metal hydroxides, accelerating the reaction process and promoting the formation of QDs [93–95]. Simultaneously, the inert gas such as argon (Ar) must be introduced into the reactors for avoiding oxidation of MQDs. However, considering the surface of MXenes covered by oxygen-containing groups (–OH, –O), higher reaction temperature or longer reaction time can lead to surface oxidation due to the dissolution of oxygen groups. Xue et al. [58] pointed the connection between reaction temperature and final product; when it is above 150 °C, the MXene-derived carbon quantum dots (CQDs) was formed due to the dissolution of metallic Ti. Also, the crystallinity of MQDs decreases with the increasing temperature (Fig. 5c-h), confirmed by X-ray diffraction (XRD) (Fig. 5i).

The mechanism of solvothermal reaction is the same as the aforementioned technique, but the solvent is organic, such as N, N-Dimethylformamide (DMF), Dimethyl sulfoxide (DMSO), and ethanol. Therefore, the formation of MQDs is related to the boiling point, and oxidation ability of solvents. The Ti_3_C_2_ MQDs was prepared by using different organic solvent, showing that MQDs prepared by DMSO have a reduced quantum yield and photoluminescence properties, attributed to high boiling point and oxidation ability of DMSO (Fig. 5j-k) [96]. Moreover, as the reaction temperature determines the size of MQDs due to the solubility difference of MXene in the solvent, MQDs usually show excellent solubility in both the water and ethanol [97]. Also, the strong quantum confinement of MQDs induced by size effect further affects photoluminescence (PL) behavior. Similar to other QDs, the PL undergoes blue shifts as the size of MQDs decrease, which is one of the viewpoints in the mechanism of luminescence. However, the products of MQDs vary with the reaction temperature. As a consequence, it results in different surface composition, further affecting fluorescence behavior. Currently, it remains a challenge for clarification of the fluorescence mechanisms of MQDs [98].

Moreover, the mechanics-assisted methods (e.g*.*, sonic tip, bath sonication, and agitation) have become alternative to the hydrothermal or solvothermal methods. The process can bypass the necessity of the high temperature and high pressure, but an inert gas protection is required. The mechanism of mechanics-assisted preparation relies on the layer cutting and stacking cleavage. MXene materials are sensitive to surface functional groups. The functional groups (e.g*.*, –O, –OH, –F, and –Cl) cannot avoid being introduced into the surface due to the intrinsic liquid-phase exfoliation process [99–101]. Many reports have confirmed the type of groups impact on the electrochemical performance of the MXene-based materials [40, 102, 103]. Furthermore, it is important for synthesis F-free MXenes, which contributes to improve the electrochemical activity [104]. Jang et al. prepared oxygen-functionalized MXenes by alkalized and heat process for hydrogen evolution reaction (HER), and the result is consistent with the previous calculation, oxygen sites as catalytic active sites provide ideal Gibbs free energy for hydrogen adsorption (△G_H*_) [105, 106]. Compared to 2D MXenes, the F-free 0D MQDs have the same property. The Ti_3_C_2_(OH)_2_ MQDs with hydroxyl groups modification prepared by the alkalization treatment and mechanical agitation method for electrochemical N_2_ reduction [70]. The experiment combines with computational findings confirmed that the –OH functional groups and abundant Ti edges contributed to the obtained outstand ammonia production performance. Such method expects to be extended to a wide range of MQDs-based catalytic systems.

**Fig. 6:**
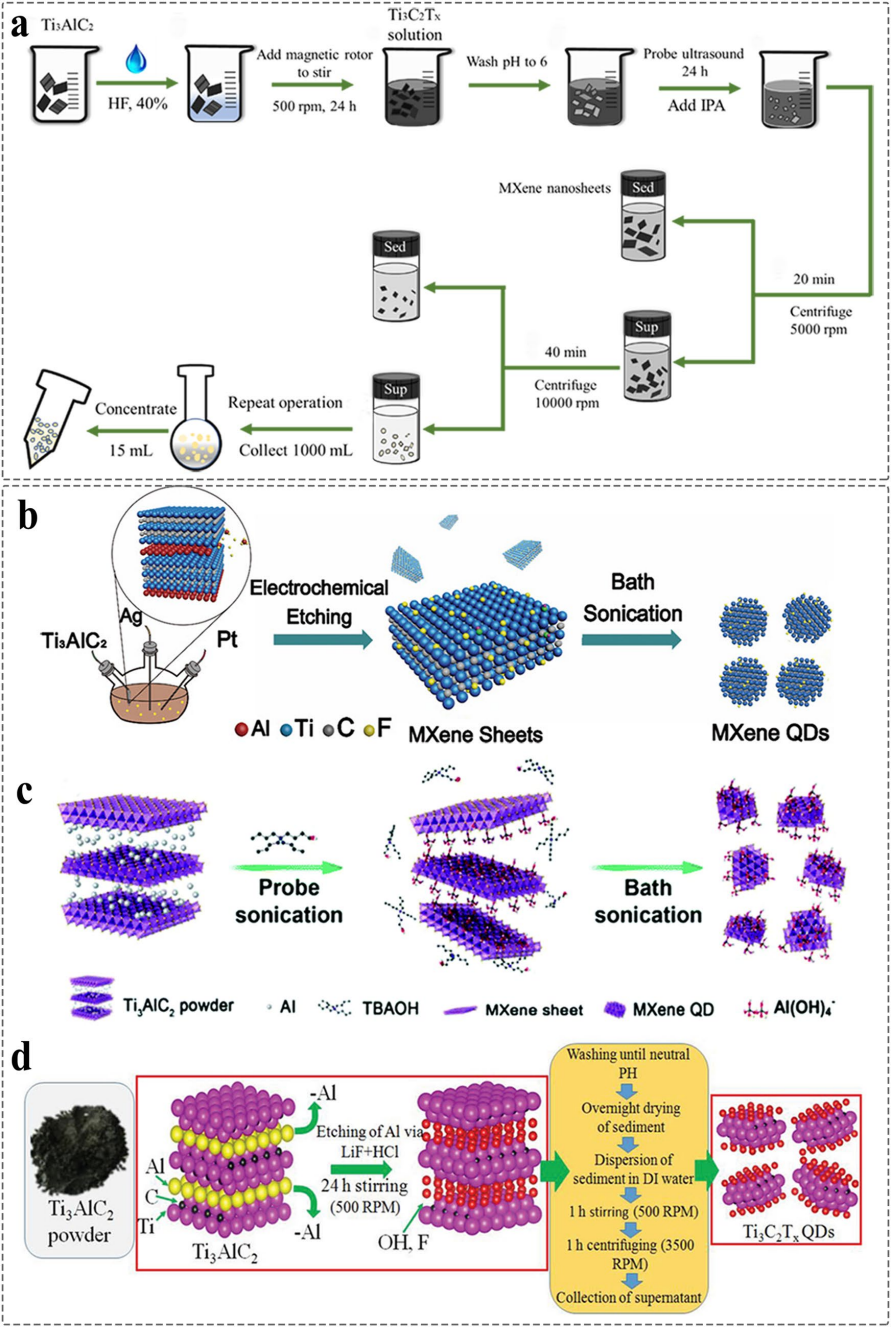
Schematic illustration for the synthesis of MQDs by using different methods. **a** Probe ultrasound [108]. Copyright @2020, Wiley–VCH. **b** Bath sonication [109]. Copyright @2020, American Chemical Society. **c** A combination of probe sonication and bath sonication [110]. Copyright @2017, The Royal Society of Chemistry. **d** Mechanical stirring method [111]. Copyright @2021, Wiley–VCH

However, the probe sonication depends on high power probe to break MXene nanosheet into small-sized MQDs (Fig. 6a) [108]. The probe of sonic tip is selective to the size and hardness of raw MXene materials. Thus, it is important for establishing the correlation to prepare our expected MQDs. The discipline is yet to be explored. In addition, the bath sonication needs the protection of low temperature for preventing surface oxidation caused by overheating (Fig. 6b) [109]. The method of bath sonication is time-consuming. Therefore, the choice of the appropriate intercalation solvent affects the subsequent preparation, and the parameters of power and time affect the production yield and size of MQDs. Furthermore, the size and concentration of MQDs are also related to the amount of solution and final centrifugation speed due to the quality difference between MXene nanosheet and MQDs [110]. Sometimes, it is more convenient and safer to prepare MXene QDs by F-free probe sonication combined with bath sonication (Fig. 6c). Mechanical stirring has attracted extensive attention due to the advantages of simple and low cost (Fig. 6d) [111]. There are merely three articles that reported the method so far. In addition, there are some methods for the synthesis of MQDs. For example, the Ti_3_C_2_ MQDs have been prepared through reflux [112], ball-milling, and microwave-assisted method [113, 114]. The emerging technologies such as ultrafast shaped laser [115], micro-explosion [87], potential static and acoustomicrofluidic method remain in an exploratory stage [116, 117].

Generally, the preparation of QDs is either top-down or bottom-up. Although the former has been wieldy used, the disadvantage of the complicated synthetic process, time-consuming and low yield requires to develop highly efficient methods. Compared to the top-down, the main synthesis mechanism of the latter lies at the cross-linking and polymerization through small molecules, thereof leading to controllable structure, size, composition, and morphology of QDs. So, the atomic utilization is maximized, thus obtaining the desired type of QDs [118]. However, there are seldom reports that MQDs were prepared by using bottom-up methods, mainly due to the issue that MQDs must simultaneously satisfy two points: (1) inheriting the structure of MXenes; (2) holding the physicochemical properties of QDs. It is worth mentioning that the composites of both Mo_2_C QDs/carbon nanosheets and Mo_2_C QDs/carbon polyhedron were prepared via bottom-up style, i.e., molten salt method and pyrolysis method, respectively [119, 120]. Although they are not clearly defined as the MQDs, such simple, low-cost, and high-yield method is expected to a successful preparation of MQDs.

Currently, the production yield of MQDs is rarely referred, and the reported technique applied for further improving the yield remains a challenge. In addition, there is a key issue that the process of preparation produces small amount of metal oxidation in the surface of MQDs. Therefore, more efforts will be made and explored for preparing MQDs of the high purity.

### Synthesis of Surface-modified MQDs

MQDs inherit abundant surface functional groups of the MXenes, including oxygen (–O), hydroxyl (–OH), chlorine (–Cl), or fluorine (–F) [121]. Gogotsi group reported the Ti_3_C_2_ MXene containing the aforementioned groups was synthesized in water solution, which shows the ζ-potential of about −40 mV [90], indicating the groups are negatively charged. Therefore, various of organic/inorganic molecules, ions, and atoms were used as surface modification/functionalization [122–125] of the MQDs through the electrostatic interaction or physical adsorption to improve the stability, selectivity, conductivity, quantum yield, and photoluminescent properties [126–128]. Furthermore, 2D MXenes possess the excellent flexible, natural hydrophilicity, and the MQDs with the same structure are easily combined with other functional materials to form composites, producing a heterogeneous material by integrating their advantages. Also, MQDs possess strong quantum confine effect compared to 2D MXenes, and the MQDs can be as co-catalyst to control the energy band structure. Based on the different modifiers, the synthesis of surfaced-modified MQDs is summarized in Table 1. However, there are a few articles to address the surface chemical of MQDs on the catalytic research, so we only emphasize the common synthesis methods.

**Table 1:** Synthesis routes of surface-modified MQDs

Surface-modified MQDs		Sample	Synthesis	Solvents/reaction atmosphere	Size (nm)	Applications	Refs.
Surface-modified MQDs	MQDs modified by heteroatoms	N-Ti_3_C_2_	Hydrothermal	Ethylenediamine	2–7	Environmental/biomedical	[147]
		N, P-MQDs	Hydrothermal	Phosphate (DAP)	2.73 ± 0.50	Cu^2+^ detection	[144]
		S, N-Ti_3_C_2_	Hydrothermal	Na_2_S_2_O_3_ NH_3_·H_2_O	50	Light-emitting diodes	[133]
		N-Ti_3_C_2_	Solvothermal	oPD	7.5	Detection of ARS	[148]
		N-Ti_3_C_2_	Solvothermal	DMF	6.2	Cu^2+^ detection	[149]
		S, N-Nb_2_C	Hydrothermal	L-cysteine	2.6–4.7	Biological sensing	[86]
		S, N-Nb_2_C	Hydrothermal	L-cysteine, urea	3.54	Cells imaging	[130]
		N, B-Ti_3_C_2_	Hydrothermal	Boric acid, ammonia	2.25 ± 0.55	Testing of tetracycline	[142]
		N-Ti_2_C	Hydrothermal	EDA	-	Antioxidants	[145]
		N-Ti_3_C_2_	Solvothermal	DMF	3.09 ± 0.04	Sensor	[150]
		Cl, N-Ti_3_C_2_	Potential static	Ammonium hydroxide	3.45	Hydroxyl Radical Scavenging	[117]
		N-Ti_3_C_2_	Hydrothermal	Ethylenediamine	4	Fluorescence imaging	[23]
		N-Ti_3_C_2_	Hydrothermal	Tetramethylammonium hydroxide	-	H_2_O_2_ Detection	[107]
		Co-Ti_3_C_2_	Hydrothermal	NH_3_·H_2_O	6.66	Photoelectrochemical Water Oxidation	[72]
		Eu-Ti_3_C_2_	Hydrothermal	NH_3_·H_2_O	2.81	detector	[146]
MQDs-based heterostructure	MQDs modified by organic molecules	Amino-Ti_3_C_2_	Hydrothermal	NH_4_·H_2_O	2.73	Diagnosing histidine	[140]
		N-Ti_3_C_2_	Hydrothermal	Ethylenediamine	3.32	Mucin 1 detection	[141]
		PLL-Ti_3_C_2_	Hydrothermal	ε-Poly-L-lysine	3	Fluorometric determination of cytochrome c and trypsin	[151]
		Glutathione–Ti_3_C_2_	Hydrothermal	Glutathione, deionized water	2.5	Fluorescence probe	[152]
		N-Ti_3_C_2_ @DAP	Solvothermal	2,3-diaminophenazine NH_3_·H_2_O	3.4 ± 0.5	Detect H_2_O_2_	[153]
		Uric acid–Ti_3_C_2_	Microwave	Water	50 ± 0.5	Fluorescence probe	[112]
		BSA@Ti_3_C_2_	Hydrothermal	Bovine serum albumin	2	Fluorescence probe	[154]
		MQD-PVP	Hydrothermal	Polyvinylpyrrolidone	3	Nonvolatile Memory Devices	[155]
	0D MQDs/0D heterostructure	CsPbBr_3_QD/Ti_3_C_2x_ QD	Hot-injection	Ar	-	Photoluminescence probe/photodetector	[156]
		Ni@Ti_3_C_2_	Hydrothermal	ethylene glycol	5.96	Cr (VI) reduction	[77]
	0D MQDs/1D heterostructure	Au NRs/ Ti_3_C_2_ QDs	Hydrothermal	1% trisodium citrate	1–6	Photoelectrochemical water splitting	[157]
		Ti_3_C_2_/Au NB	Microwave	TMAOH	4.13	Sensor	[158]
		Ti_3_C_2_ QDs/Cu_2_O NWs/Cu	Self-assembly	Ar	–	Electrocatalytic CO_2_	[67]
		WO_3_/TQDs/In_2_S_3_		ethylene glycol	1,66 ± 0.04	Environmental remediation	[74]
	0D MQDs/2D heterostructure	BiVO_4_ @ZnIn_2_S_4_ /Ti_3_C_2_ QDs	Ultrasonication-stirring	Water	10	Photocatalytic water splitting	[68]
		TiO_2_/C_3_N_4_/Ti_3_C_2_	Self-assembly	NH_3_·H_2_O	3	Photocatalytic CO_2_	[159]
		Ti_3_C_2_ QDs/SiC	Self-assembly	ultrapure water	–	Photocatalytic NO	[71]
		NiFe LDH/ Ti_3_C_2_ QDs/NG	Urea-assisted co-precipitation	N-methylpyrrolidone	5	Zinc–air batteries	[136]
		Ti_3_C_2_ QDs/WS_2_	Dry Transfer Technique	-	5	-	[111]
		g-C_3_N_4_@Ti_3_C_2_ QDs	Self-assembly	Vacuum	–	Photocatalytic hydrogen production	[160]
		Ti_3_C_2_ QDs/N–C	electrostatically adsorb	Deionized water	5–6	Li–O_2_ Batteries	[137]
		S, N-Ti_3_C_2_ QDs/SnO_2_	ultrasonication	Deionized water	–	Perovskite solar cells	[138]
		MoO_x_/Ti_3_C_2_ QDs	spin-coating	–	–	Photoelectrochemical water splitting	[161]
		Ti_3_C_2_ QDs/Cu nanosheet	–	CuSO_4_ aqueous solution	4.97	N_2_ Electroreduction	[79]
		Ti_3_C_2_ QD/LRGO	–	Ar	1.5–4.5	Transparent supercapacitors	[115]
		Ti_3_C_2_Cl_2_@NiAl-LDHs	Electrostatic assembly	N–N-dimethylformamide	4–10	Pseudocapacitor	[143]
		Ti_3_C_2_ QD/Ni-MOF	Ultrasonic	ethanol, DMF, TEA	4.19	N_2_ Photoreduction	[69]
		NiCo-LDH @Ti_3_C_2_ QDs	Hydrothermal	DI water	3.06 ± 0.78	Supercapacitor	[162]
	0D MQDs/3D heterostructure	Ti_3_C_2_ QDs/g-C_3_N_4_	Self-assembly	DI water	2–10	Photocatalytic H_2_O_2_	[73]
		Ti_3_C_2_ QDs/ TiO_2_	Laxly self-organized	Water	8.2	Photoelectrochemical biosensing	[163]
		Ti_2_CO_x_ QDs/Cu_2_O/Cu foam	Electrostatic assembly	hydrochloric acid	2.98 ± 0.62	Electrocatalytic hydrogen production	[164]
		C_3_N_4_/r-Ti_3_C_2_	Self-assembly	Ar	5.2 ± 0.97	N_2_ photofixation	[76]
		Ti_3_C_2_-QDs/ZnIn_2_S_4_/Ti	Impregnation	DI water	2–5	Photocatalytic	[78]
		Ti_3_C_2_/watermelon peel aerogels	Soak	DI water	< 10	Hydrogen Evolution	[165]

#### MQDs Modified by Single/Dual Heteroatoms

MQDs have been applied to biomedical [129, 130], optical device [131–133], energy storage [134–138], and sensor fields [112, 139, 140] due to that they possess the advantages of non-toxicity, metal conductivity, excellent chemical stability and low cost. Currently, the research of pure MQDs cannot meet the development needs of practical application. The surface-modified MQDs by heteroatoms, still in its infancy stage, have intrigued great research enthusiasm. It can be seen from Table 1 that such heteroatoms are almost all non-metallic such as nitrogen (N) [139, 141, 142], phosphorus (P) [114], sulfur (S) [133] and chlorine (Cl) [117, 143]. The common synthesis methods are hydrothermal. However, the improvement of properties of MQDs modified by using metal atoms is just on the beginning.

**Fig. 7:**
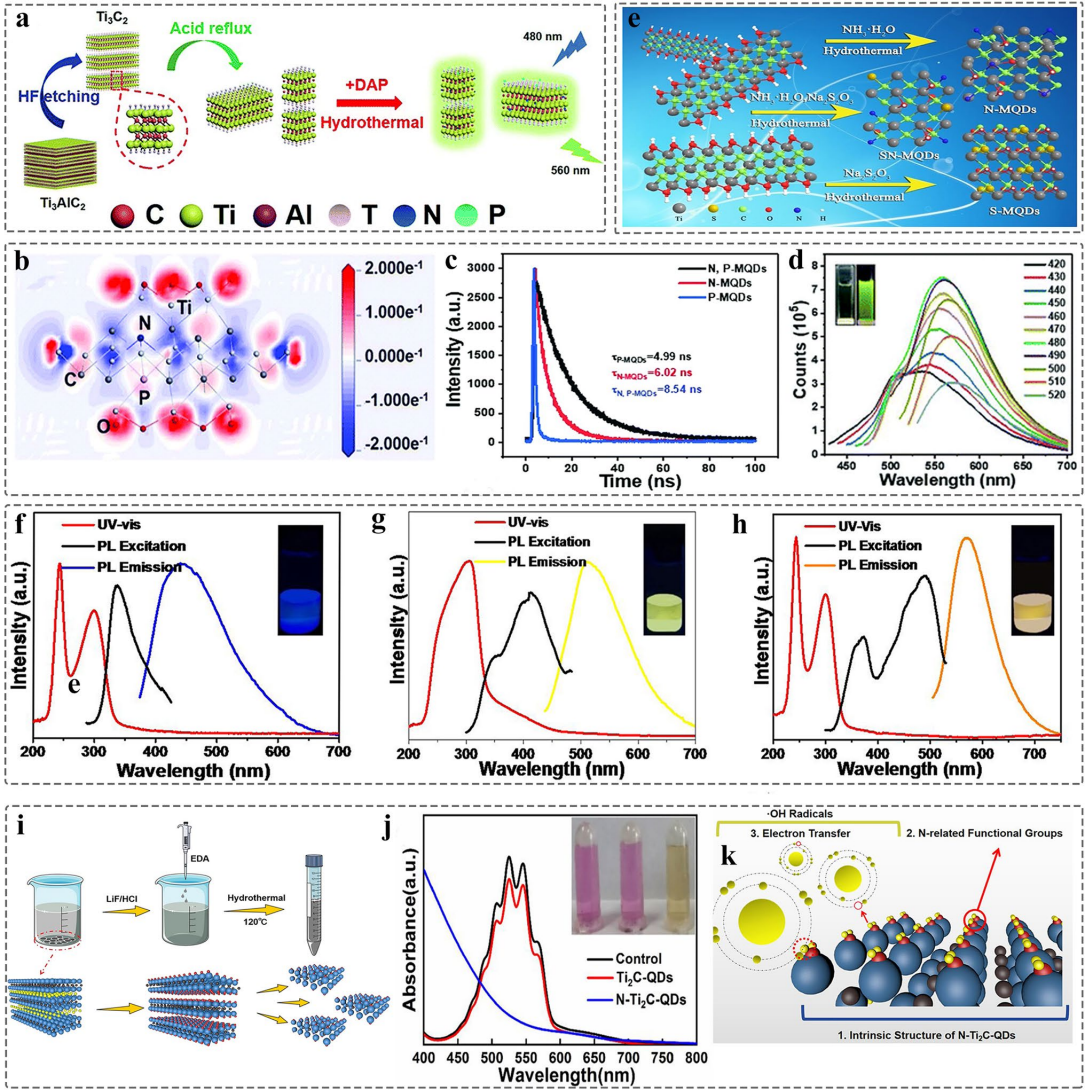
Schematics, structural and optical behavior characterizations of MQDs modified by single/dual heteroatoms. **a** Schematic illustration of the synthesis of N, P-Ti_3_C_2_ MQDs; **b** Charge density difference of N, P functionalized Ti_3_C_2_ MQDs; **c** Fluorescence emission spectra of N, P-Ti_3_C_2_ MQDs; **d** Photoluminescence decay spectra of the N-Ti_3_C_2_ MQDs, P-Ti_3_C_2_ MQDs, N, P-Ti_3_C_2_ MQDs [144]. Copyright @2019, The Royal Society of Chemistry. **e** Schematic illustration of the synthesis of S-Ti_3_C_2_ MQDs, N-Ti_3_C_2_ MQDs, S, N-Ti_3_C_2_ MQDs; **f–h** UV–Vis adsorption spectra of S-Ti_3_C_2_ MQDs, N-Ti_3_C_2_ MQDs, S, N-Ti_3_C_2_ MQDs [133]. Copyright @2019, Elsevier. **i** Schematic illustration of the synthesis of N-Ti_2_C MQDs; **j** Antioxidants performance test at KMnO_4_ solutions; **k** Mechanism of antioxidants [145]. Copyright @2021, American Chemical Society

Generally, the strong electronegativity of non-metallic atoms is beneficial to passivate the active sites of MQDs, leading to the change of electronic structure, and thereof producing surface defects. Thus, some obvious changes will occur for the physicochemical properties of MQDs [86]. As shown in Fig. 7a, Guan et al. prepared N, P-doped Ti_3_C_2_ MQDs with green fluorescence and size of 2.93 nm by hydrothermal method, and density functional theory (DFT) calculation reveals the electron transfer from P to N facilitates to improve the fluorescence (Fig. 7b-d). Compared to pure MQDs and single-atom-doped MQDs, the favorable electron transfer can enhance the photoluminescence quantum yield (PLQY) of 20.1% [144].

Likewise, The PLQY is an important parameter for judging the performance of QDs in the fields of biological, sensing, and optoelectronic devices. The S, N-Nb_2_C MQDs with an average size of 2.66 nm was synthesized, enhancing the QY of Nb_2_C and the stability by optimizing the PL properties. It is known the PL properties of QDs are related to size, surface composition, and pH. The non-metal doping often occurs at carbon sites with a larger shrinkage of the defect-induced bond of MQDs, leading to a variety of fluorescence [130]. In addition, in 2019, the S, N-doped Ti_3_C_2_ MQDs prepared by hydrothermal method (Fig. 7e), achieving the multiple-color emissive from blue to orange light (Fig. 7f-h) [133], which will benefit for the mankind in the field of energy storage, photocatalysis, medicine and biology. MXenes are easily oxidized due to the dissolved oxygen and oxygen-contain groups, especially in the high temperature and pressure condition [58]. Therefore, this heteroatoms modification can also enhance the antioxidant capacity of MQDs. For example, the ethylenediamine (EDA) was introduced into the surface of MQDs as the additive, forming the surface electron-rich N-Ti_3_C_2_ MQDs (Fig. 7i) [145]. The method not only avoids the surface oxidation of MQDs, and retains the intrinsic structure of MXenes, but also enhances the antioxidant ability, enabling N-Ti_3_C_2_ MQDs as effective reductants (Fig. 7j-k). Apart from the above-mentioned issue, S, N co-doped Ti_3_C_2_ MQDs [133], Cl, N co-doped Ti_3_C_2_ MQDs [117], and N, B co-doped Ti_3_C_2_ MQDs [142] have been prepared by using the same method to improve their physical–chemical properties. Furthermore, the Ti_3_C_2_ MQDs modified by metal atom also contribute to enhance energy transfer process, leading to enhance the sensitive of detector [146].

Apart from non-metallic elements as the dopants to control the functional application, the metal atom modification is helpful to adjust the energy level structure of MQDs, thereby achieving highly catalytic active sites. Tang et al. synthesized Co-Ti_3_C_2_ MQDs with a Janus-structured style by using Co ion thermal-anchoring reaction and ammonia-assisted hydrothermal method [72]. The introduction of Co constructs the Schottky junction, produces the rectifying effect, promoting effectively the photogenerated carrier separation/injection efficiency. It shows the excellent photoelectrochemical water oxidation capability.

Although the doped MQDs have made great progress, the application prospect in the catalytic field is still unknown, whether the MQDs make the breakthroughs like other inorganic QDs (e.g., CQDs, GQDs, and MoS_2_ QDs) in the future is something worth investigating. Except for such finding, whether the modification of organic molecules also brings the considerable improvement of properties?

#### MQDs Modified by Organic Molecules

Organic molecules have been applied to modify the surface of QDs to improve the fluorescence responses in aqueous solution, enhanced the application in biological and optical fields [166–168]. However, such molecules modified MQDs have little application in catalysis. Compared to the non-metal and metals, organic molecules have the advantages of low toxicity, low cost, easily biodegradability and better biocompatibility and so on. It was reported that such molecules are usually adsorbed to the surface of MQDs by physical absorptions or electrostatic interactions, contributing to improve the compatibility of MQDs, leading to the enhancement of dispersion, mechanical, and fire retarded properties [169, 170]. For instance, the glutathione functionalized Ti_3_C_2_ MQDs prepared by hydrothermal method, the MQDs of surface passivated by glutathione show outstanding fluorescence stability regardless of any pH value or time, which is a promising fluorescence probe [152]. Such surface modification facilitates to the stabilization of surface energy traps, leading to surface state luminescence with excitation independence [84]. Furthermore, MQDs have excellent stability of the PL intensity at different pH values, so MQDs-based nanomaterials are an ideal sensor [58].

Additionally, uric acid (UA) was used as ligand to enhance photophysical property. Wang et al. [112] prepared UA@Ti_3_C_2_ MQDs by facile microwave-assisted strategy. 2D Ti_3_C_2_ MXene was broken into 0D MQDs based on the acid etching and the high power. The UA as reaction solvent forming the large molecules that encase Ti_3_C_2_ MQDs. The method is not only easily operation, but also enhanced the oxidation resistance and highly quantum yield of MQDs. Apart from the above-mentioned molecules, the 2,3-diaminophenazine (DAP), ε-Poly-L-lysine (PLL), polyvinylpyrrolidone (PVP), and bovine serum albumin (BSA) were used to synthesis functionalized MQDs, thereby promoting the MQD in application to biomedical and physical fields [151, 153–155]. Furthermore, MQDs possess abundant hydrophilic functional groups. To avoid the self-aggregation of MQDs, improving the stability during the synthesis and reaction, the organic molecules were introduced onto the surface of MQDs, as possibly an effective method to increase the yields.

#### MQDs-based Heterostructures

Like single atoms and other 2D inorganic QDs, the MQDs with small-size affect easily the aggregation due to their high surface energy during synthesis and reaction process [96, 171]. For catalysts, we not only pursuit excellent conductivity, low cost, environmentally friendly, and outstanding performance, but also the durability of operation. Constructing the MQDs/support heterostructure is an effective strategy. Such hierarchical heterostructures contributes to adjusting the band structure, achieving the excellent Catalytic activity. We will introduce the routine synthesis routes of heterostructure between 0D MQDs and different dimension support in the following section.

##### 0D MQDs/0D Nanomaterials

**Fig. 8:**
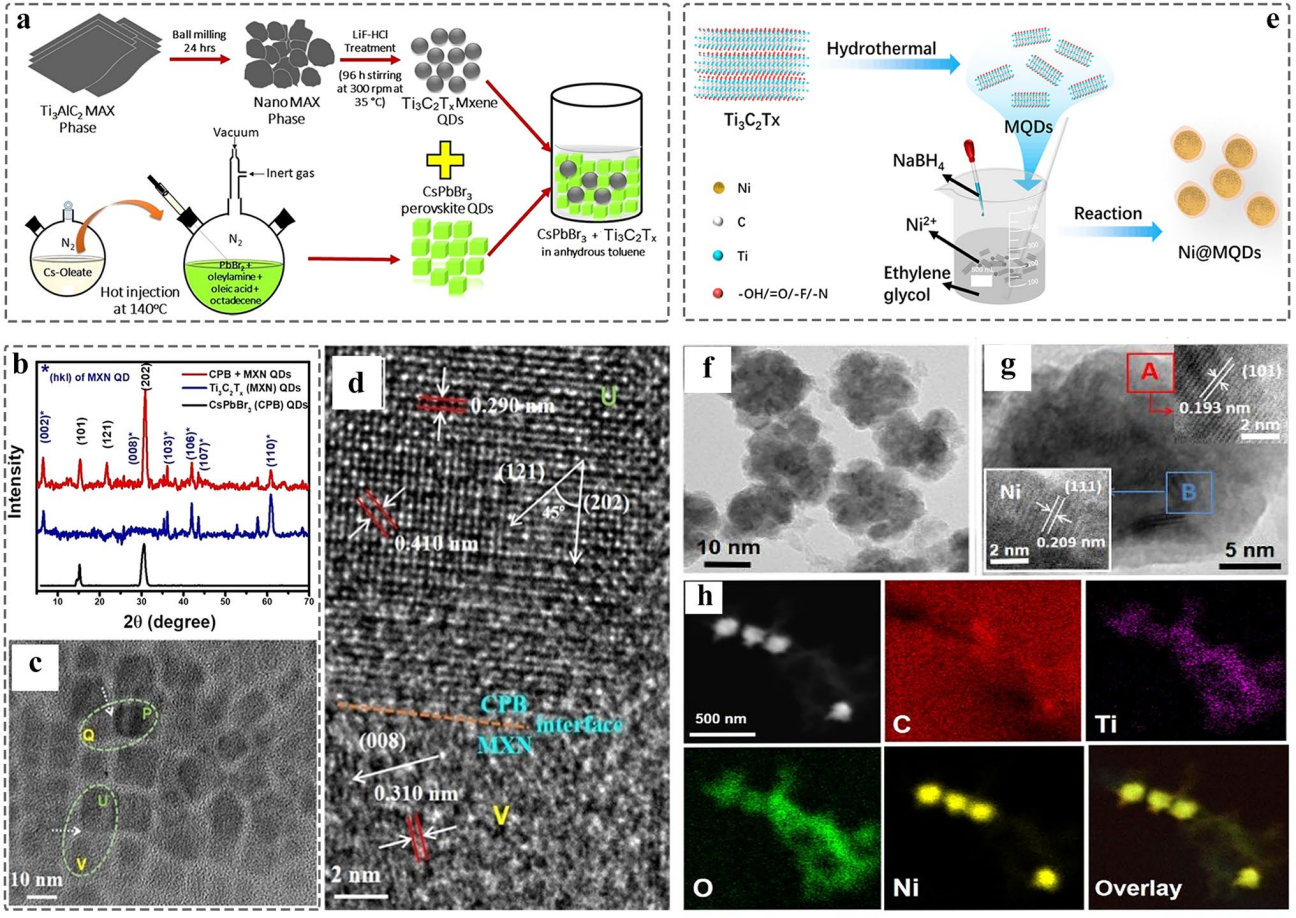
Schematic and morphological and structural characterizations of 0D MQDs/0D nanocomposite. **a** Schematic illustration of the synthesis of CsPbBr_3_–Ti_3_C_2_T_x_ MQD/QD; **b** XRD patterns of CsPbBr_3_ QDs, Ti_3_C_2_T_x_ MQD, CsPbBr_3_–Ti_3_C_2_T_x_ MQD/QD; **c** TEM image of CsPbBr_3_–Ti_3_C_2_T_x_ MQD/QD; **d** HRTEM image of CsPbBr_3_–Ti_3_C_2_T_x_ MQD/QD [156]. Copyright @2020, American Chemical Society. **e** Schematic of the formation of Ni@Ti_3_C_2_ MQDs; **f-g** TEM image of Ni@Ti_3_C_2_ MQDs. insets of A and B represent HRTEM image of Ni and MQDs, respectively; **h** EDS of Ni@ Ti_3_C_2_ MQDs [77]. Copyright @2022, Elsevier

The electronic coupling at the interface is essential for regulating the electronic structure and producing efficient charges transfer, which contributes to an improved electrochemical reaction process and device performance [172, 173]. 0D nanomaterials has the lateral size range of 0.1 ~ 100 nm. Currently, there are few reports on the composite of MQD with other 0D nanomaterials, and such improving physical chemical properties is expected to be further explored. In 2020, the CsPbBr_3_ QDs-Ti_3_C_2_T_x_ MQD heterostructure was constructed by facile ultra-sonicating method (Fig. 8a) [156]. XRD of CPB-MXene QD/QDs composites retain the crystal structure of CsPbBr_3_ QDs and MQDs, and no impurity phase was found (Fig. 8b). The morphology of nanocomposite is shown in Fig. 8c, and the corresponding high-resolution transmission electron microscopy (HRTEM) image is displayed in Fig. 8d. The local magnification of “U” and “V” represent the lattice fringes of CsPbBr_3_ QDs and MQDs, respectively. The interface (orange dashed line) relies on the strong interaction between the functional groups on the surface of MQDs and Cs^+^, causing the photoluminescence (PL) quenching due to the charge transfer from Cs^+^ to MQDs. However, when Cs^+^ was introduced into the heterostructure again, the PL will recovery. Therefore, the 0D/0D heterostructure is expected to apply to ion detection and photodetector.

Currently, there are reports for the introduction of single atoms (SAs), nanoparticles into inorganic QDs such as cadmium–zinc sulfide quantum dots (ZCS QDs), carbon quantum dots (CQDs), graphene quantum dots (GQDs), and so on [174–177]. Compared to the bulk support, the exposure of specific crystalline planes can be precisely controlled, contributing to the synergistic effect between the SAs and coordinating elements, and the coordination environment of SAs can be regulated for increasing the selectivity of products [178]. Interestingly, MQDs possess the same properties as other inorganic QDs, but were endowed with the abundant surface groups (–OH, –O, –Cl, or –F). Therefore, MQDs are a promising support catalyst. The Ti_3_C_2_ MQDs coated Ni nanoflowers were synthesized by using facile reduction reaction for wastewater treatment (Fig. 8e), the transmission electron microscopy (TEM) and HRTEM images confirmed the core–shell structure (the core: Ni flowers, the shell: MQDs), and the corresponding lattice fringes (Fig. 8f-g) [77]. The elemental mapping shows that the Ti/C/O/Ni were uniformly distributed in the surface of nanocomposites (Fig. 8h). Such interfacial interaction not only avoids any aggregation of Ni nanoparticles, but also lowers the catalytic reaction activation energy of Cr (VI). It is of great interests to extend 0D/0D heterostructures to other catalytic fields.

##### 0D MQDs/1D Nanomaterials

**Fig. 9:**
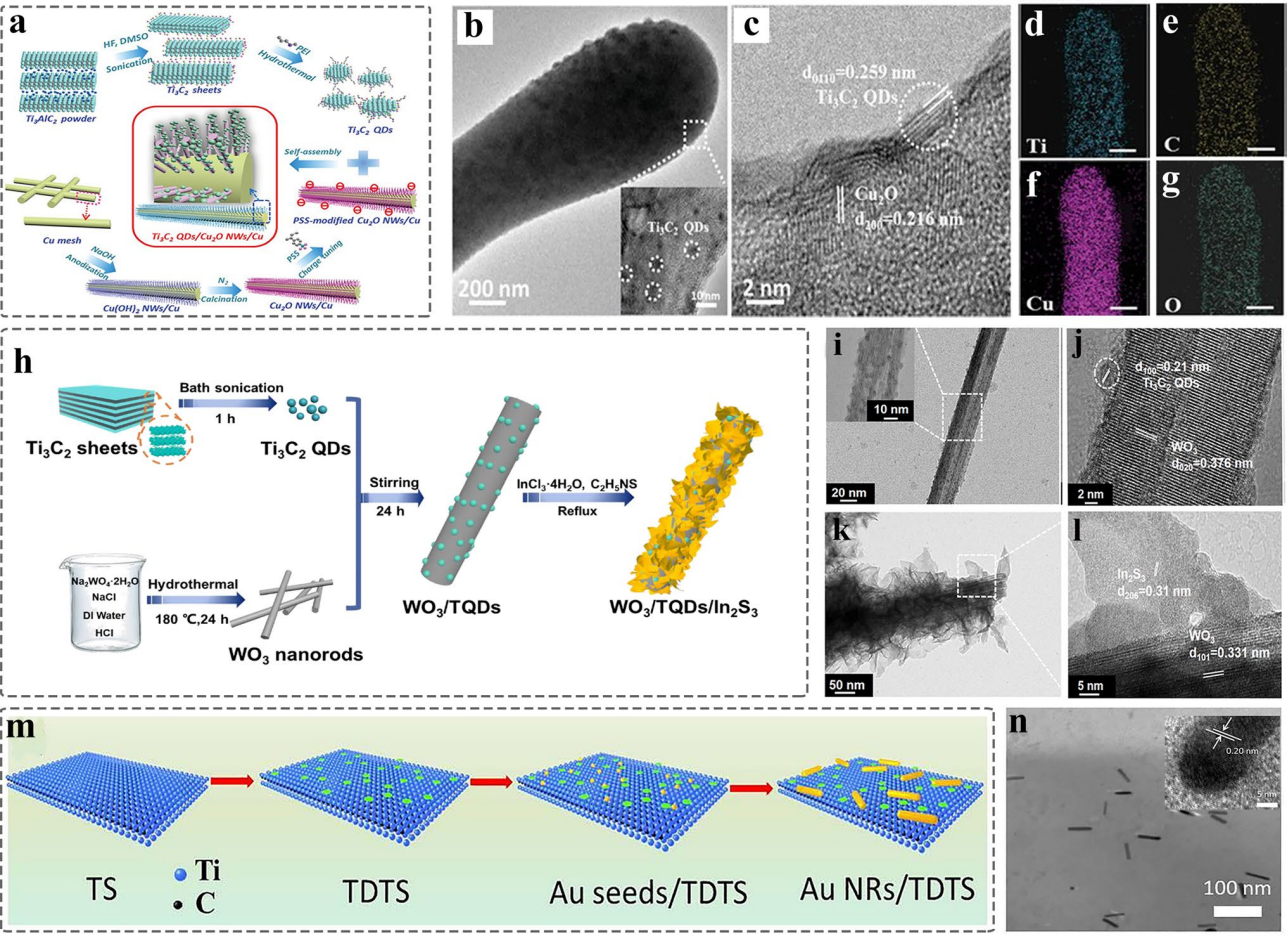
Schematic and morphological and structure characterizations of 0D MQDs/1D nanomaterials heterostructure. **a** Synthesis process of Ti_3_C_2_ QDs/Cu_2_O NWs/Cu heterostructure; **b** TEM image of the Ti_3_C_2_ QDs/Cu_2_O NWs heterojunction; **c** HRTEM image of the interface in Ti_3_C_2_ QDs and Cu_2_O; **d-g** EDX elemental mapping of Ti_3_C_2_ QDs/Cu_2_O NWs [67]. Copyright @2019, WILEY–VCH. **h** Schematic illustration of WO_3_/TQDs/In_2_S_3_ heterostructure; **i** TEM image of WO_3_/TQDs; **j** HRTEM image of WO_3_/TQDs; **k** TEM image of WO_3_/TQDs/In_2_S_3_; **l** HRTEM image of WO_3_/TQDs/In_2_S_3_ [74]. Copyright @2021, Elsevier. **m** Schematic for the preparation of Au NRs/Ti_3_C_2_ MQDs/Ti_3_C_2_ nanosheets; **n** TEM image of Au NRs/TDTS. Inset illustration is HRTEM image of Ti_3_C_2_ MQDs [157]. Copyright @2021, Elsevier

1D nanomaterials allow electrons-dominating transfer, mainly including nanotubes, nanowires, nanorods, and nanobelts [10, 179]. Transition metal oxides are commonly used as ideal photocatalyst due to their adequate optic bandgap. However, the regulation of bandgap facilitates to hinder the photogenerated carriers’ recombination, achieving an efficient surface redox reaction. Zeng et al. [67] prepared Ti_3_C_2_ MQDs/Cu_2_O nanowire composite by using electrostatic self-assembly strategy for highly efficient photocatalytic CO_2_ conversion (Fig. 9a). TEM image confirmed the MQDs dispersed in the surface of Cu_2_O nanowires, and the corresponding HRTEM characterization confirmed the formation of heterogeneous interface (Fig. 9b-c), and 0.216 nm and 0.219 nm of lattice fringes are attributed to Ti_3_C_2_ MQDs _*(0110)*_ and Cu_2_O _*(200)*_, respectively. In addition, the energy dispersive X-ray (EDX) spectra displayed the Ti/C/O/Cu dispersed uniformly in the surface of Cu_2_O (Fig. 9d-g). Combined the DFT and experiment shows that the MQDs as co-catalyst to promote the separation of carriers and decrease the band bending edge, enhancing the light adsorption capability and the transport of carriers of Cu_2_O. Furthermore, the 1D nanowires not only provide long light adsorption path and short charge transport distance, but also enable quickly collecting the separated photogenerated carriers.

Likewise, the WO_3_/Ti_3_C_2_ QDs/In_2_S_3_ with Z-scheme heterostructure was fabricated by using facile solution method (Fig. 9h). TEM and HRTEM images confirmed that the In_2_S_3_ nanosheet and MQDs with an average size of 1.66 ± 0.04 nm were uniformly dispersed on surface of WO_3_ nanorods (Fig. 9i), and the WO_3_ was coated by In_2_S_3_ nanosheets (Fig. 9k), corresponding to the interface between the Ti_3_C_2_ MQDs and WO_3_ (or WO_3_ and In_2_S_3_) shown in Fig. 9j (Fig. 9l). This report shows that MQDs is an ideal co-catalyst to promote the separation of the photogeneration carriers, achieving efficient Cr (VI) reduction and photocatalytic oxidation of the BPA [74]. In addition, the MQDs were used as co-catalyst to promote photocatalytic water splitting due to their broader photoresponse and excellent conductivity. In 2021 [157], the Au nanorods/Ti_3_C_2_ MQDs heterostructure was prepared via electrostatic interaction (Fig. 9m). TEM image showing the plasmonic gold nanorods (NRs) were distributed in the Ti_3_C_2_ MXene QDs-interspersed Ti_3_C_2_ nanosheet (TDTS) (Fig. 9n), and the corresponding HRTEM image of Au _*(200)*_ is displayed in the inset of Fig. 9n. In addition, the Ti_3_C_2_ MQDs @Au nanobones were also prepared by using the seed-mediated growth method and self-assembly for exploring the improving performance in the biomedical application.

##### 0D MQDs/2D Nanomaterials

**Fig. 10:**
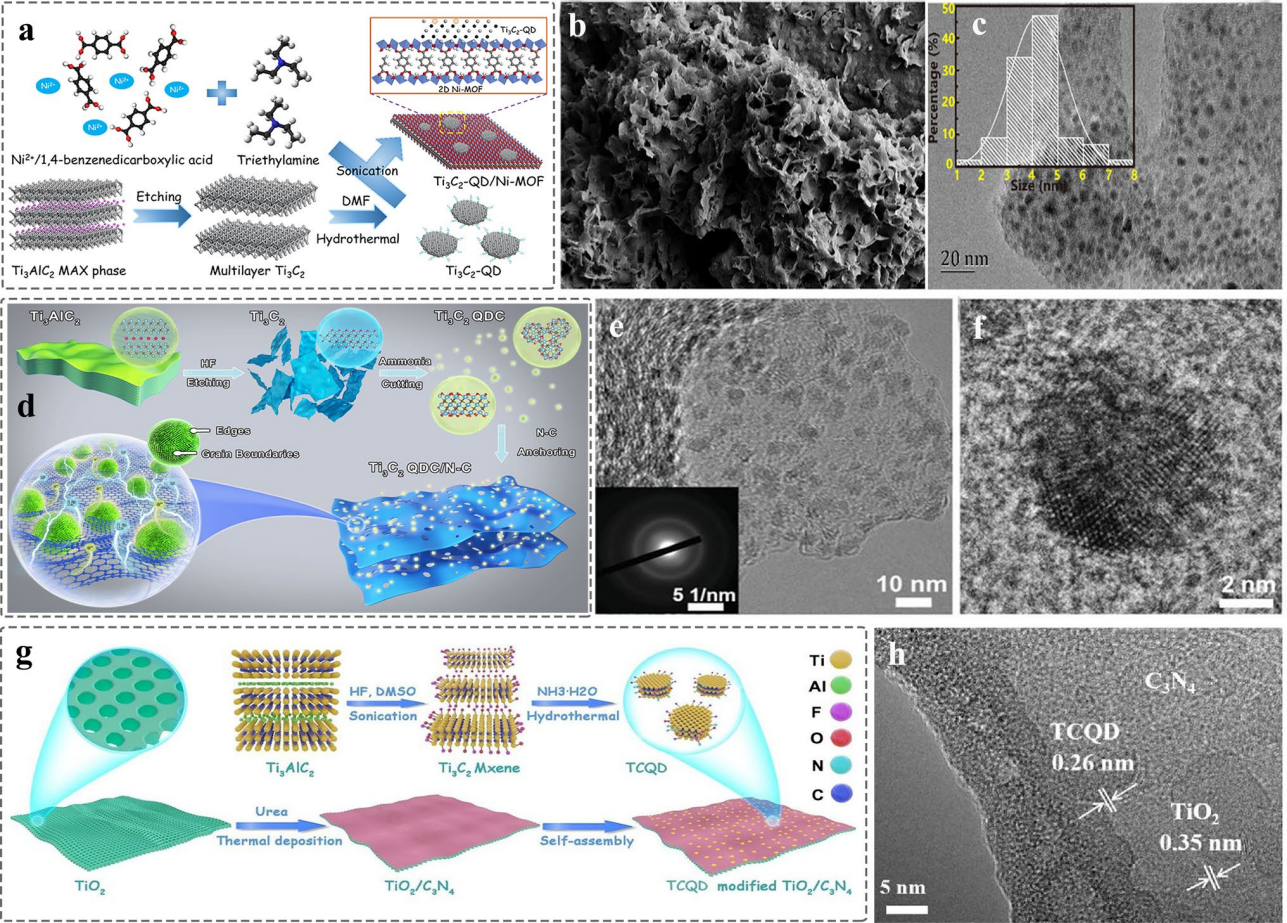
Schematic and morphological and structure characterizations of 0D MQDs/2D nanosheets heterostructure. **a** Schematic diagram of Ti_3_C_2_ MQDs/Ni-MOF; **b** SEM images of Ti_3_C_2_ MQDs/Ni-MOF; **c** TEM images of Ti_3_C_2_ MQDs/Ni-MOF. The inset illustration is the size distribution of Ti_3_C_2_ MQDs. Reproduced with permission [69]. Copyright @2020, American Chemical Society. **d** Synthesis process schematic of Ti_3_C_2_ QDC/N-C nanocomposites and **e** TEM image of Ti_3_C_2_ QDC/N-C, inset illustration is SAED pattern; **f** HRTEM image of Ti_3_C_2_ QDC/N-C [137]. Copyright @2021, Wiley–VCH. **g** Schematic preparation of Ti_3_C_2_ MQDs/TiO_2_/C_3_N_4_ hierarchical structure; **h** HRTEM image of T-CN-TC heterostructure [159]. Copyright @2020, Elsevier

Compared to 0D and 1D support materials, 2D nanomaterials are referred to as that the electrons motion is unrestricted in two directions, which has lager planar size. As a result, it provides the abundant basal plane that is active with a number of anchored sites [128, 180, 181]. Currently, many reports address the preparation of 2D few-layer or monolayer nanosheets by using chemical vapor deposition, organic solvent intercalation, liquid-phase exfoliation strategy and electrospinning, and so on [182–184], The introduction of intrinsic defects such as vacancies, lattice distortions and adatoms on the surface of graphene, g-C_3_N_4_ and MoS [185–187], is beneficial to improve the physical properties of materials such as electronic conductivity. In 2020, the MQDs were used as co-catalyst to enhance the photocatalytic activity of 2D metal–organic framework (MOF). The Ti_3_C_2_ MQD/Ni-MOF catalyst was prepared by self-assembly strategy (Fig. 10a) [69]. SEM image revealed the 2D nanosheet morphology of Ni-MOF (Fig. 10b), and the HRTEM image confirmed the MQDs with an average size of 4.19 nm that were uniformly dispersed on the surface of Ni nanosheet (Fig. 10c). The presence of MQDs helps to enhance the light absorption and interface charge transfer ability, promoting an efficient N_2_ photoreduction reaction. Besides, the 0D/2D heterostructure is also applied to energy storage. Moreover, the defect-rich MQDs cluster/N-doped carbon nanosheet nanocomposites were prepared by using electrostatically self-assembly for Li–O_2_ batteries (Fig. 10d) [137]. TEM image shows the uniform distribution of MQDs in the N–C nanosheets (Fig. 10e). However, as shown in inset of Fig. 10e, the quantum size effect MQDs contributes to the poor crystal quality of MQDs/N–C nanocomposites. This result can be proved by using HRTEM (Fig. 10f). Combining the experiment and DFT reveals the MQDs with abundant grain boundaries and edge defects as the active origins for increasing the adsorption of O_2_ molecules and intermediates LiO_2_. Controlling the number of interfaces is of great significance for improving the structure of photocatalyst and enhancing the performance. The 2D/2D/0D (TiO_2_/C_3_N_4_/Ti_3_C_2_ MQDs) hierarchical structure was engineered by van der Waals and electrostatic interactions (Fig. 10g) [159]. The TiO_2_/C_3_N_4_ exhibits core–shell structure, and the MQDs decorated on the surface of C_3_N_4_ nanosheet in three to four layers, and the corresponding HRTEM image is shown in Fig. 10h. Such ultrathin three-phase interface and the introduction of MQDs help to increase the transport channels of charges, providing the abundant photogenerated carries. In addition, g-C_3_N_4_ nanosheets [160], nanofilms [138], SiC [71], and graphene [115] 2D nanomaterial have been prepared for loading 0D MQDs, improving the performance in electrocatalysis, photocatalysis, and supercapacitors.

##### 0D MQDs/3D Nanomaterials

**Fig. 11:**
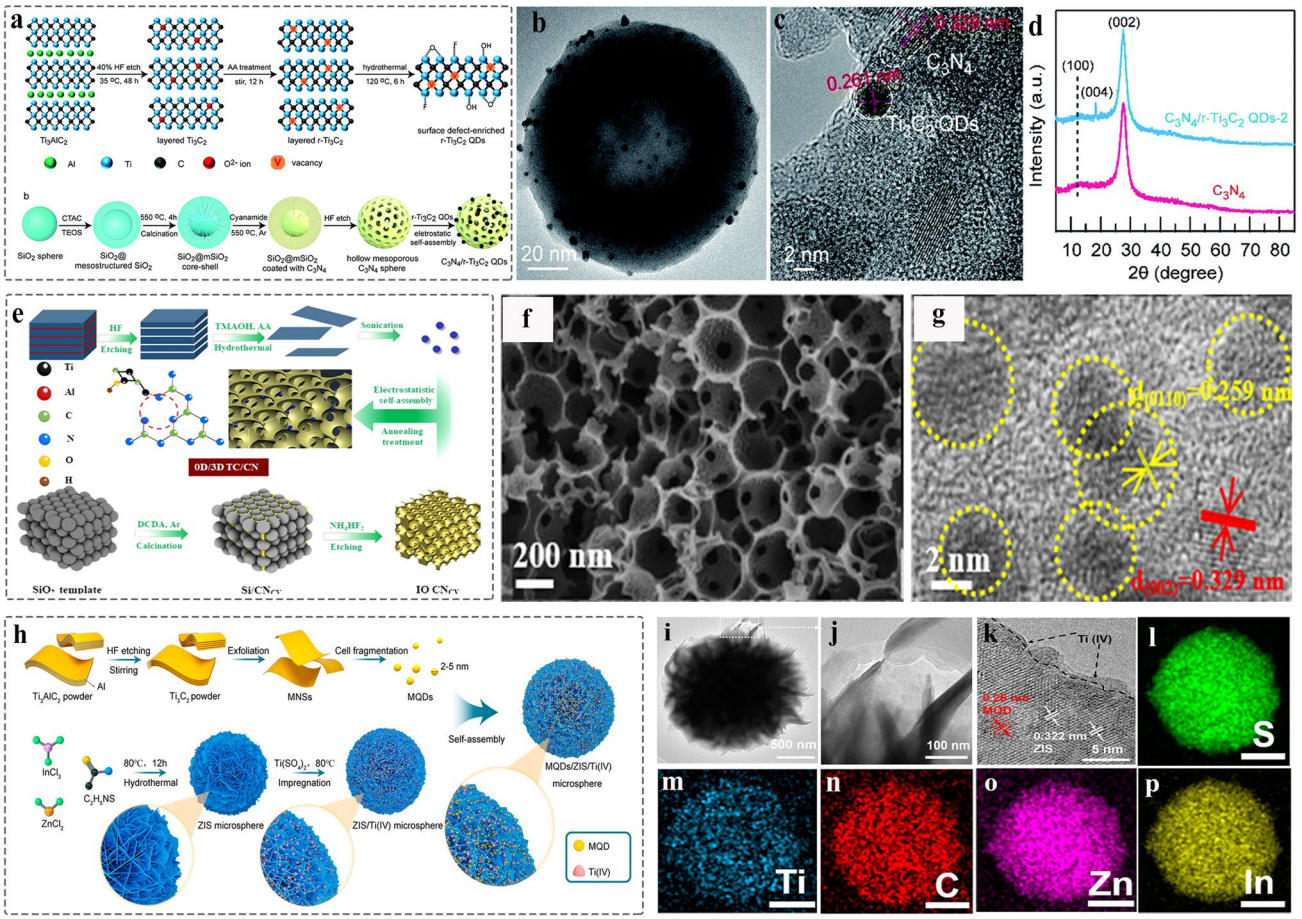
Schematic and morphological and structure characterizations of 0D/3D heterostructure. **a** The prepared process diagram of C_3_N_4_/r-Ti_3_C_2_ QDs; **b** TEM image of C_3_N_4_/r-Ti_3_C_2_ QDs; **c** HRTEM image of C_3_N_4_/r-Ti_3_C_2_ QDs; **d** XRD pattern of C_3_N_4_, C_3_N_4_/r-Ti_3_C_2_ QDs [76]. Copyright @2022, The Royal Society of Chemistry. **e** Schematic preparation of Ti_3_C_2_ MQDs/3D Inverse Opal g‑C_3_N_4_ heterojunction; **f** SEM image of TC/CN-20 after adding 20 mL of MQDs solution and 20 mL water; **g** HRTEM image of TC/CN-20 [73]. Copyright @2020, American Chemical Society. **h** Schematic illustration of Ti_3_C_2_-QDs/ZnIn_2_S_4_/Ti(IV) heterostructure; **i-k** TEM image of Ti_3_C_2_-QDs/ZnIn_2_S_4_/Ti(IV) at different magnifications; **l**-**p** Elemental mappings of Ti_3_C_2_-QDs/ZnIn_2_S_4_/Ti(IV) [78]. Copyright @2022, MDPI and the authors

Compared to other dimensional nanomaterials, 3D nanomaterials with porous structure provides the abundant gas diffusion channel and interface sites, and favorable for reactant diffusion direction [188, 189]. MQDs have served as co-catalyst to avoid the recombination between the photogenerated electrons and photogenerated holes. Recently, constructing 0D/3D heterostructure has been reported to make MQDs as electron acceptor to promote surface redox reaction. In 2022, the Ti_3_C_2_ MQDs with the surfaces detect-rich/3D mesoporous C_3_N_4_ were prepared by electrostatically self-assembly strategy (Fig. 11a) [76]. TEM image confirmed that the MQDs were uniformly dispersed on the surface of hollow C_3_N_4_ (Fig. 11b), and the HRTEM image gives the corresponding lattice spacing of 0.329 and 0.261 nm, attributed to the plane of C_3_N_4 (002)_ and Ti_3_C_2 (0110)_, respectively, indicating the formation of Schottky junction (Fig. 11c). The XRD analysis for the weak signal of MQDs correlate with the low content (Fig. 11d). Meanwhile, such MQDs-induced Schottky junction catalyst was used to promote the photocatalytic H_2_O_2_ production.

Lin et al. [73] prepared the Ti_3_C_2_ MQDs decorated defective inverse opal g-C_3_N_4_ (TC/CN) by using electrostatic self-assembly method (Fig. 11e). SEM image shows the microstructure of porous g-C_3_N_4_ with a long-range order (Fig. 11f), and the corresponding HRTEM confirmed the formation of the interface between MQDs and g-C_3_N_4_ (Fig. 11g). Such bonding contributes to achieve the carrier separation. In addition, the Ti_3_C_2_ MQDs/ZnIn_2_S_4_/Ti (IV) 3D hierarchical structure was constructed by impregnation and self-assembly methods (Fig. 11h) [78]. TEM and HRTEM images show that the Ti (IV) and MQDs are uniformly dispersed on the surface of 3D nanoflowers microspheres (Fig. 11i-k). The EDX elemental mapping confirmed the elements were uniformly dispersed in the surface of microsphere (Fig. 11l-p). The nanocomposites were used as co-catalyst to promote long-term stability. In addition, various of MQDs-based heterostructure has been designed such as MQDs/3D bio-aerogels [165], and the TiO_2_/MQDs [163] meet the growing needs of biomedical application, photoelectronic sensor, biosensor, and photocatalysis.

In summary, the properties difference of 0D MQDs-based heterostructure (0D/0D, 0D/1D, 0D/2D, 0D/3D) are mainly rooted from the variation of the support properties with different geometric structures. Whereas the coordination environment between the MQDs and their support is flexible and controllable, independent of the dimension of the support, and it determines the optimal performance to be achieved. Furthermore, the MQDs as catalyst offers abundant catalytic active sites. In order to maximize the utilization of active sites, achieve the fast electron transport channels, and ensure the efficient and stable working of catalysts, the support with different dimensions is often selected to optimize the overall performance. Also, the morphology of catalysts also needs to be considered to meet the application requirement.

### Other MXene-Derived Inorganic QDs

**Fig. 12:**
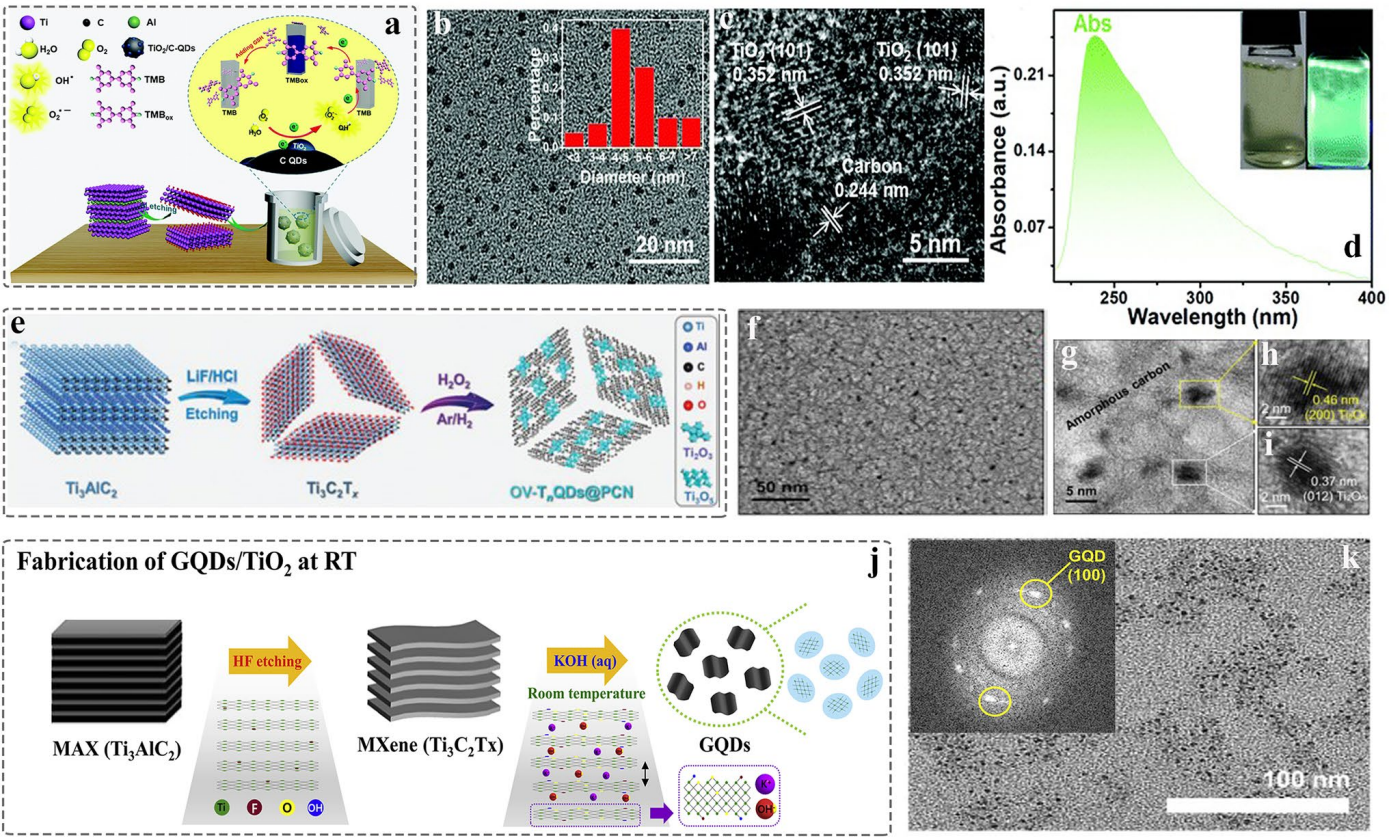
Schematic and morphological and structure characterizations of MXene-derived inorganic QDs. **a** Schematic of synthesis for TiO_2_/C-QDs; **b** TEM image of TiO_2_/C-QDs, the inset illustration is size distribution of TiO_2_/C-QDs; **c** HRTEM image of TiO_2_/C-QDs; **d** UV–Vis adsorption spectra of TiO_2_/C-QDs [88]. Copyright @2020, The Royal Society of Chemistry. **e** Illustration of preparation of oxygen-vacancy-rich Ti_n_O_2n−1_ QDs @PCN; **f-g** TEM image of OV–T_n_ QDs @PCN at different magnification; **h-i** HRTEM image of Ti_n_O_2n−1_ QDs @PCN with different number of oxygen vacancies [90]. Copyright @2021, Wiley–VCH. **j** Schematic illustration of the preparation of graphene quantum dots (GQDs); **k** TEM image of GQDs, and the inset illustration is size distribution of GQDs [195]. Copyright @2020, Elsevier

According to the previous reports, the MXenes will expose inner carbon layer, or produce a small number of amorphous carbon due to the partial dissolution of external M metal atoms during etching process, which can be proved by using Raman spectrum [7, 190, 191]. In 2017, Sun et al. [192] prepared F-free Ti_2_CT_x_ via electrochemical etching under the HCl aqueous solution. The result shows that such method enables easily exfoliating Ti layers, producing carbide-derived carbon (CDC), which is related to voltage, etching time, and electrolyte concentration. Furthermore, most of MXenes are sensitive to oxygen atmosphere, facilitating the formation of transition metal oxides in the surface [193, 194]. Therefore, many inorganic QDs such as carbon dots (CDs), graphene quantum dots (GQDs), and transition metal oxide QDs can be prepared by using such material-derivatives, which provides facile, safe, and environmentally friendly method to prepare inorganic QDs. In 2020, the TiO_2_ QDs supported on the surface of carbon layer were prepared by solvothermal method using small and fewer-layered Ti_3_C_2_ MXene nanosheets (Fig. 12a) [88].

The condition of high temperature and high pressure induced the oxidization of MXene surface via the dissolved oxygen in solution. TEM and HRTEM images confirmed the formation of TiO_2_/C-QDs with an average of 5.23 ± 0.3 nm (Fig. 12b-c), and the UV–Vis adsorption spectrum shows that QDs have adsorption peak at 250 nm, and the inset of Fig. 12d corresponds to the optical photo-blue TiO_2_/C-QDs in daylight under 365 nm excitation wavelength. Moreover, the oxygen-vacancy-rich Ti_n_O_2n−1_ QDs (OV–T_n_QDs) were prepared by H_2_O_2_ oxidation and subsequent quenching in liquid nitrogen (Fig. 12e) [90]. The quench process makes TiO_2_ nanoparticles fast crystallization and downsized to quantum size. After that, the annealing process with H_2_/Ar mixed gas promotes the generation of O vacancies. TEM image confirmed the uniform distribution of OV–T_n_ QDs (Fig. 12f). The corresponding HRTEM image exhibit that the OV–T_n_ QDs are made up of Ti_2_O_3_ and Ti_3_O_5_ (Fig. 12g-i).

We all know that the carbon dots are used in solar energy cell, optoelectronic, and biomedical applications due to low cost, environmentally friendly and non-toxic, and excellent biocompatibility. Currently, carbon dots are abundant in raw materials such as carbon nanotube [196], carbon-containing organic molecules [197], and biomass materials [198]. However, 2D MXene-derived CDs are rarely reported. In 2021, the 2D Ti_3_C_2_T_x_ MXene-derived (CDs) was prepared by hydrothermally [199]. Also, the GQDs were reported through controlling the alkalized time and concentration of 2D MXenes treated by KOH (Fig. 12j) [195]. The previous articles reported that KOH has been used as activator to promote the formation of micropore in the carbon-based nanomaterials [99, 200]. Therefore, the Ti-C covalent can be broken under the alkaline condition, producing the Ti and C-based nanoparticles and finally with formation of the CDs, or a small amount of amorphous C, and the metal oxides. As shown in Fig. 12k, the GQDs/TiO_2_ nanoparticles with an average size of 1.5 nm. In addition, solvothermal strategy is also used to prepare GQDs [201]. Such carbon-based nanomaterials derived QDs possess low cost, excellent photoluminescence properties and stability, expect to apply in the fields of energy storage, devices, and imaging.

## Characterization Techniques of MQDs

**Fig. 13:**
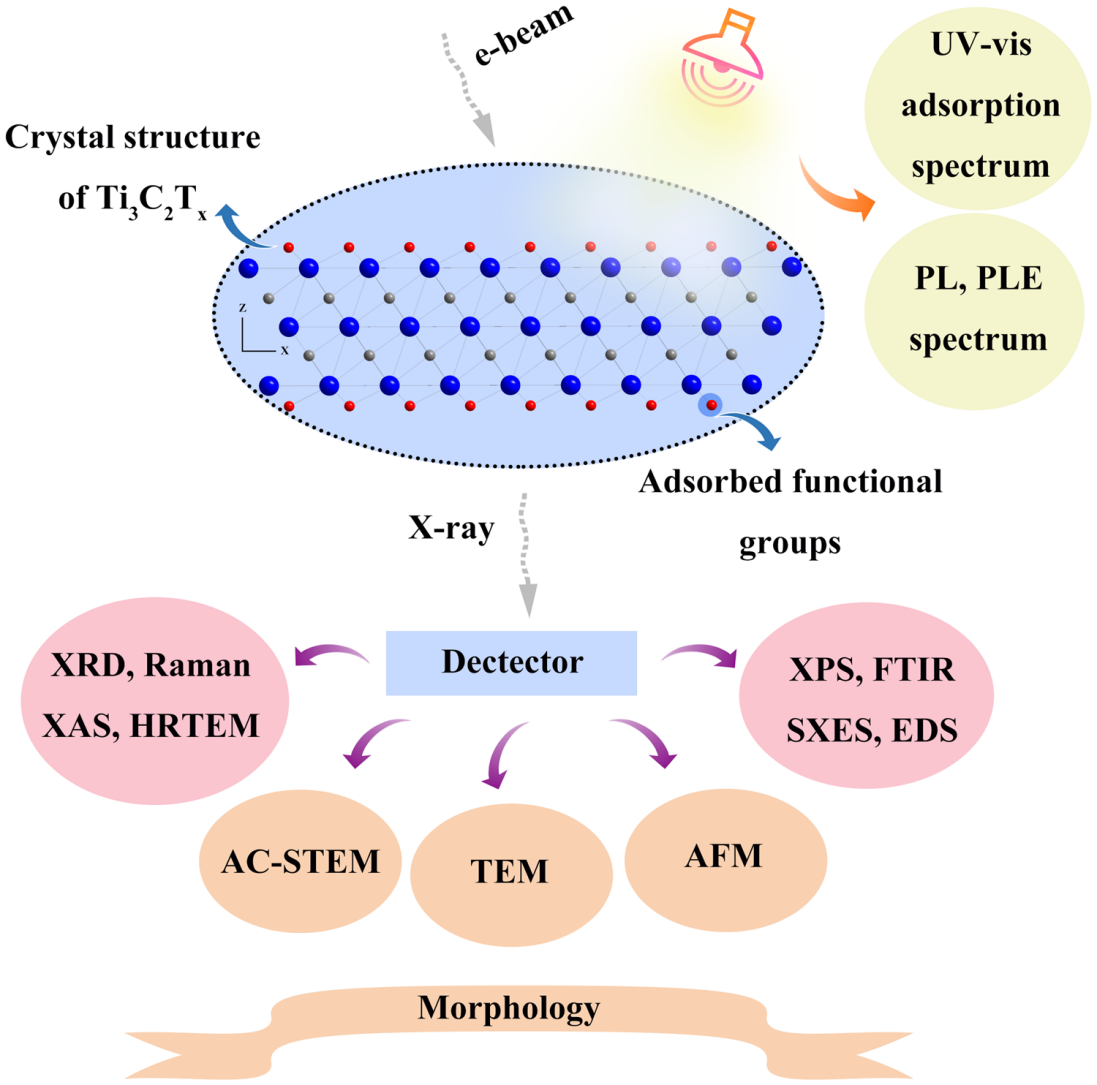
Characterization techniques of MQDs

Generally, structure determines the performance of the materials, which is important for design of the catalysts with the specific functions. The MQDs derived from 2D MXene, changing the synthesis routes of the MXenes, which will produce different kinds of functional groups, or removing the groups by some post-processing, thereby impacting on various properties of MQDs (conductivity, adsorption, and magnetic applications) [99, 105]. Furthermore, the MQDs with surface functional contribute to increase amounts of active sites, simultaneously, regulate the energy band structure. Such semiconductor engineering is challenging to hinder the recombination of electron and hole in the field of photocatalysis. Therefore, it is necessary to identify such materials by using basic characterization techniques, toward promoting their further development, the comprehensive characterization techniques of MQDs shown in Fig. 13.

### Morphology Characterization

**Fig. 14:**
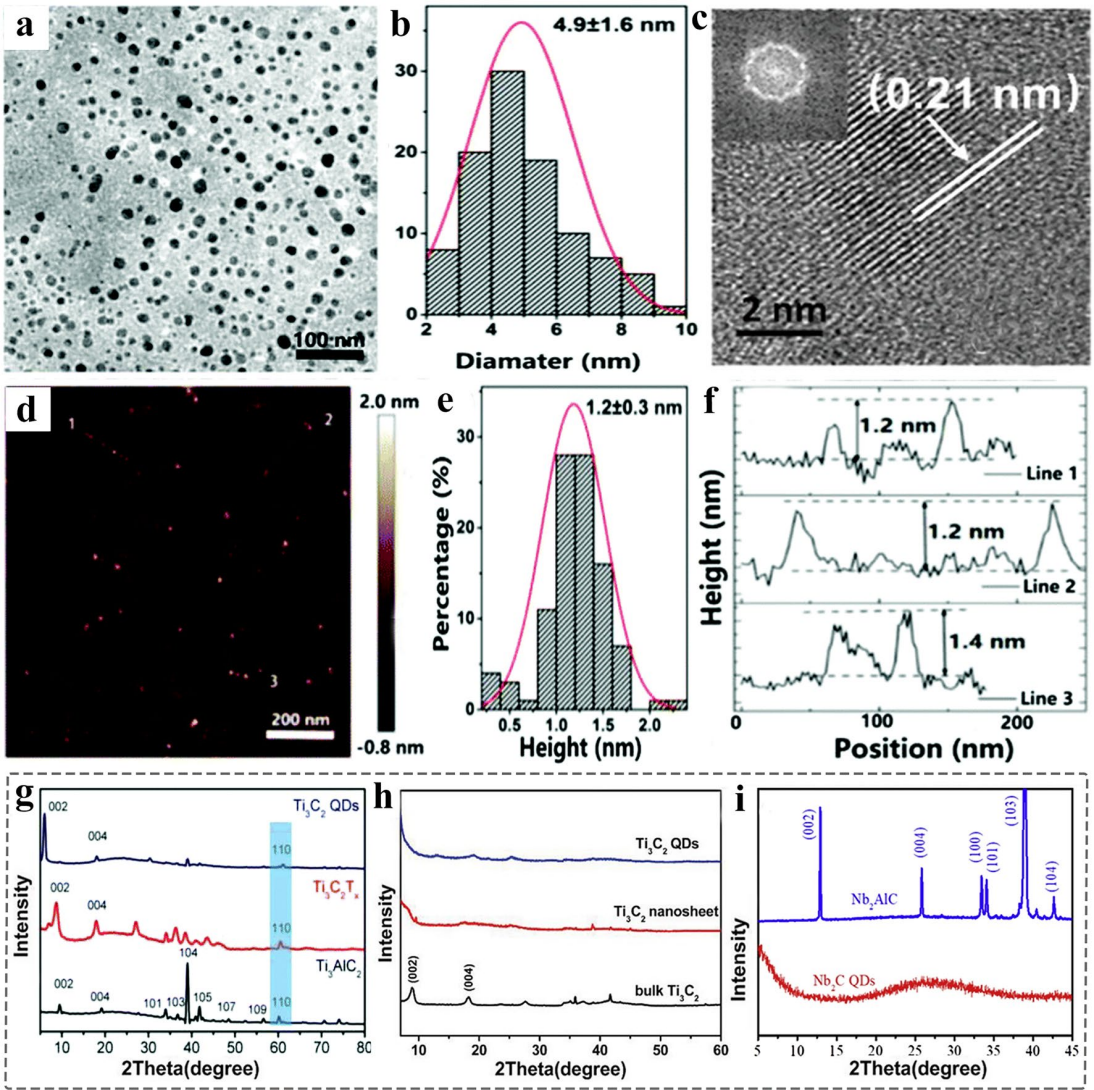
Morphology and structure characterization of MQDs. **a** TEM image of Ti_3_C_2_ MQDs; **b** Size distribution of Ti_3_C_2_ MQDs; **c** HRTEM image of Ti_3_C_2_ MQDs, the inset illustration is corresponded Fourier transform; **d** AFM image of Ti_3_C_2_ MQDs; **e** The height distribution based on AFM; **f** Height profiles of Ti_3_C_2_ MQDs along **d** image [110]. Copyright @2017, The Royal Society of Chemistry. **g** XRD patterns of Ti_3_AlC_2_, Ti_3_C_2_ MXene, and Ti_3_C_2_ MQDs [204]. Copyright @2019, The Royal Society of Chemistry. **h** XRD patterns of bulk Ti_3_C_2_ MXene, Ti_3_C_2_ nanosheet, and Ti_3_C_2_ MQDs [59]. Copyright @2019, WILEY–VCH. **i** XRD patterns of Nb_2_AlC and Nb_2_C MQDs [205]. Copyright @2020, Elsevier

Compared to their 2D counterparts, the obvious difference of MQDs lies at a series of changes in physicochemical properties due to the small-size effect [85, 202]. At present, the size of the reported MQDs is usually less than 10 nm. The morphology of MQDs is generally spherical. Since the spatial resolution of SEM usually insufficient to characterize the morphology. Therefore, TEM and atomic force microscopy (AFM) spectroscopy are often used to analyze the morphology of MQDs. They provide lateral size and height profile information, respectively. TEM images of Ti_3_C_2_ MQDs, TiCN MQDs, Nb_2_C MQDs and Ti_2_N MQDs show a size range of 4.2 ± 0.6, 2.7 ± 0.2, 1.6–4.0, and 4.83 ± 2.69 nm [87, 89, 203]. Yu et al. prepared Ti_3_C_2_ MQDs by using bath and probe sonication method [110]. TEM image shows the dot-like uniform distribution of Ti_3_C_2_ MQDs, with the average size of 4.9 ± 1.6 nm (Fig. 14a-b). HRTEM image can display the lattice fringes with an inner plane spacing of 0.21 nm (Fig. 14c), and such clearly visible lattice fringes represent a good crystallinity of MQDs [109]. AFM image indicates that the thickness was 1.2 ± 0.3 nm (Fig. 14d-f). The size distribution combined with AFM image confirms their spherical structure. Furthermore, some reports show that the lateral size of MQDs exceeds 10 nm, related to the synthesis methods, the molecular weight cut-off of the dialysis bag, and the centrifugation speed.

Currently, the aberration-corrected scanning transmission electron microscopy (STEM) (AC-STEM) has been used to identify the single atom, defects such as vacancies, atomic doping, and lattice distortions based on the super-resolution in both space and energy space [206, 207]. For example, the atomically dispersed Ni was introduced into the cadmium–zinc sulfide QDs (ZCS QDs). AC-STEM can clearly distinguish the real position of atoms, and the corresponding fast Fourier transform (FFT) pattern further proves the favorable (111) plane of Ni atoms dispersion [174]. Analogously, the coordination of single Co with S edge and strain from lattice mismatch induced the phase transition from 2H-MoS_2_ to 1T-MoS_2_. Such atomically visualizing technique directly show the different phase coordination environment and the presence of Co atoms at the 5 Å scale [208]. Currently, such spectroscopy and imaging technique has not been used to characterize 0D MQDs due to the limited development of MQDs. It is expected to be applied to the MQDs and MQDs-based nanocomposites in the future. However, it is noted that the highly electron irradiation will result in knock-on effect, as well as other electron-beam damages and changes [206, 209].

### Structure and Composition Identification

Identifying the composition and structure of matter through specific characterization techniques is essential for the development of materials science. XRD is basic characterization of phase composition and structure. However, XRD shows different peak shapes due to the high surface energy-induced aggregates [210, 211]. For example, Fig. 14g-i mainly shows three XRD patterns of Ti_3_C_2_ MQDs and Nb_2_C MQDs. Compared to 2D Ti_3_C_2_ MXene, the (002) lattice spacing was further expanded due to the intercalation of TMA ions during preparation. However, the reduced intensities of ((10* l*)), (004), and (110) diffraction peaks indicate a good dispersion of the Ti_3_C_2_ MQDs [204]. Besides, Lu et al. prepared Ti_3_C_2_ MQDs by hydrolyzing method; compared to the bulk Ti_3_C_2_, the weaker peak intensity and broad width of MQDs indicate the grain refinement [59]. More importantly, in 2020, the Nb_2_C MQDs were prepared by high-intensity ultrasonication strategy. Under the dual action of mechanical force and intercalation solvent, the strong layering effect leads to much smaller-sized MQDs without any obvious peaks and only showing the broad spreading, indicating the layered structure was completely broken down [205]. Therefore, the above results show that the peak intensity and peak width are closely correlated to the layer number and the lateral size and crystallinity of MQDs, which is across-validated with their TEM and AFM results.

**Fig. 15:**
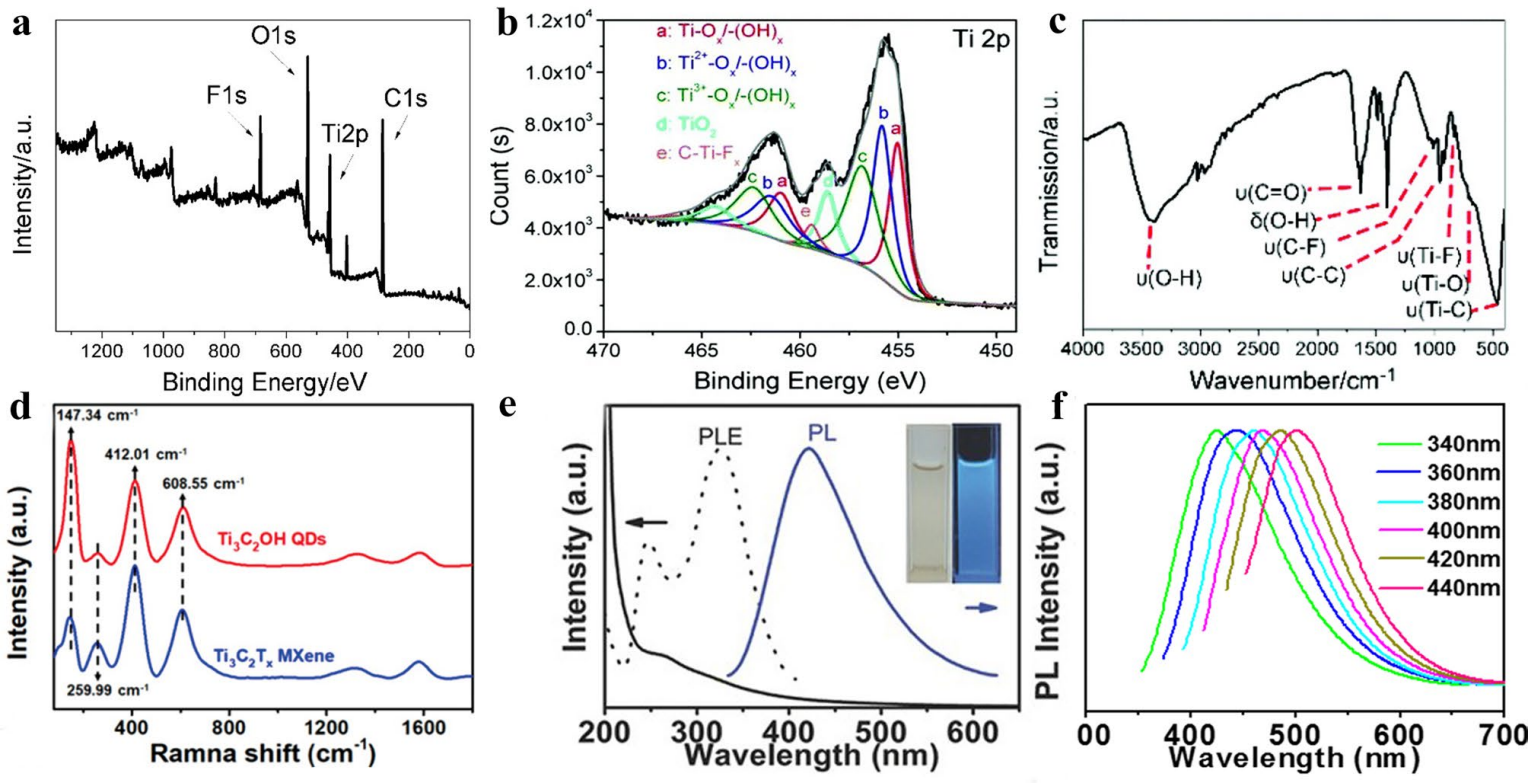
Composition and optical spectral characterization of MQDs. **a** XPS survey spectra of Ti_3_C_2_ MQDs; **b** High-resolution spectra of Ti 2p [204]. Copyright @2019, The Royal Society of Chemistry. **c** FTIR spectra of Ti_3_C_2_ MQDs [215]. Copyright @2018, The Royal Society of Chemistry. **d** Raman spectra of Ti_3_C_2_(OH)_2_ MQDs [70]. Copyright @2020, WILEY–VCH. **e** UV–Vis adsorption, PL, and PLE spectra of Ti_3_C_2_ MQDs; **f** PL spectra at different excitation wavenumbers [58]. Copyright @2017, WILEY–VCH

The unique surface chemical of MQDs can be detected by X-ray photoelectron spectroscopy (XPS) [52, 212] and Fourier transform infrared spectroscopy (FTIR) [213, 214]. They provide the surface composition, valence state, and functional groups information, respectively. As shown in Fig. 15a, the survey XPS spectrum of Ti_3_C_2_T_x_ MQDs provides all the composition elements of Ti 2*p* (457 eV), C 1*s* (285 eV), O 1*s* (529 eV), and F 1*s* (684 eV) [215]. The detailed element valance state and coordination conditions can be identified by deconvolution of the constituent element in high-resolution XPS spectra. For example, fitting the high-resolution spectrum of Ti2p by using the multi-peak Gaussian method (Fig. 15b), the binding energy peaks of 457.38, 463.18, 455.68, 461.88, and 469.98 eV can be attributed to the bond of Ti–O, Ti-C, C-Ti–O, and Ti-F, respectively [204]. According to previous report, the Ti–O comes from surface oxidation, i.e., tetravalent. Whereas the C-Ti–O, C-Ti–OH, Ti-F belong to bivalent (II) and trivalent (III). In addition, the high-resolution spectrum of each element was fitted by using the multi-peak Gaussian method. The element proportion on the surface of MQDs and the percentage of bonding were qualitatively determined according to the peak area and element sensitivity.

FTIR is another important characterization techniques of functional groups. The surface of MQDs has oxygen-containing groups due to that the synthesis is mostly solution-oriented method. For example, the vibration peak of –OH group at 3410 cm^−1^ and 1488 cm^−1^, is attributed to different vibration modes. Furthermore, the –F group has two different vibrations forms, Ti-F (830 cm^−1^) and C-F (1013 cm^−1^). The adsorption peaks of other groups such as Ti–O, Ti-C, C–C, and C = O at 701, 463, 917, and 1650 cm^−1^ (Fig. 15c) [215]. It is noted that the signal C-F and C = O originates from the breaking of the Ti-C bond during the MXene etching process, contributing to expose the inner carbon and adsorb the groups in solution [70].

Raman spectrum is also used to characterize the composition, layers, and defect intensity of QDs. For example, the 2D graphene-derived GQDs, the layers of GQDs can be judged by using characteristic G peak, the intensity, and shape of characteristic G´ peak, and the defect density of characteristic GQDs can be judged by the ratio of the D peak (1350 cm^−1^) to G peak (1580 cm^−1^) [176]. However, the present Raman characterization of MQDs elucidates the composition, while the defect states and amounts of layers remain to be further explained due to the uncertainty or the possibility of carbon exposure in the inner layer during the synthesis. Compared to 2D Ti_3_C_2_ MXene, the characteristic Raman bands of 147 cm^−1^, 260, 412, 609 cm^−1^ correspond to Ti–O and Ti-C (Fig. 15d) [54, 190, 216]. Furthermore, the D and G band comes from the exposure of inner C.

Apart from the above characterization techniques about structure and composition, other characterization of MQDs such as AC-STEM, X-ray synchrotron (XAS) [174, 217–219]. The synchrotron radiation provides a detailed ingredient analysis, including local coordination environments, valance states, and coordination number. Furthermore, the soft X-ray emission spectroscopy (SXES) based on electron microscopy can be applied to investigate the chemical bonding state of MQDs, especially in the form of nanocomposites based on the MQDs [220–222]. Besides, constructing in situ electrochemical reaction based on the synchrotron radiation means to monitor the dynamic changes of various substances in the catalytic reaction process. As a result, it reveals the catalytic reaction mechanism at the surface interface, which will help to promote the development of 0D MQDs in the field of catalysis.

### Optical Characterization

Like other organic or inorganic QDs, optical spectroscopy characterization of MQDs is obviously strong evidence for the information of MQDs. Photoluminescence spectrum (PL), photoluminescence excitation spectrum (PLE), electrochemiluminescence (ECL), and UV–Vis spectra can be used to characterize the luminous behavior of the MQDs [89, 223]. For example, the UV–Vis adsorption spectrum of Ti_3_C_2_ MQDs shows the adsorption at 320 nm, corresponding to two luminescence peaks of 250 and 320 nm in the PLE spectrum (Fig. 15e). Such UV–Vis spectra represent the different electronic transition (σ → σ*, n → σ*, π → π*) of groups. Furthermore, according to the PL spectrum at different excitation wavelengths (340–440 nm), the strong excitation-dependent PL behavior is correlated to the size effect (Fig. 15f) [58]. More importantly, the PL properties of MQDs is important for improving photocatalytic performance. It determines the light absorption range of the photocatalyst from ultraviolet to near-infrared (NIR) regions, affecting the amount of photogenerated carriers. Such PL properties are related to size, surface composition, and the pH of solution of MQDs [224]. Ti_3_C_2_ MQDs show white light, blue light in dimethyl sulfoxide (DMSO), N, N-Dimethylformamide (DMF), and ethanol, respectively, under the excitation wavelength of 365 nm [96]. Furthermore, other optical behavior should be concerned, which facilitate to understand the fluorescence mechanism of MQDs, and has great significance for promoting the development of MQDs and application in the fields of bioimaging [23, 129, 205], fluorescent probes [149, 153, 225], and optical devices [96, 108, 109].

**Fig. 16:**
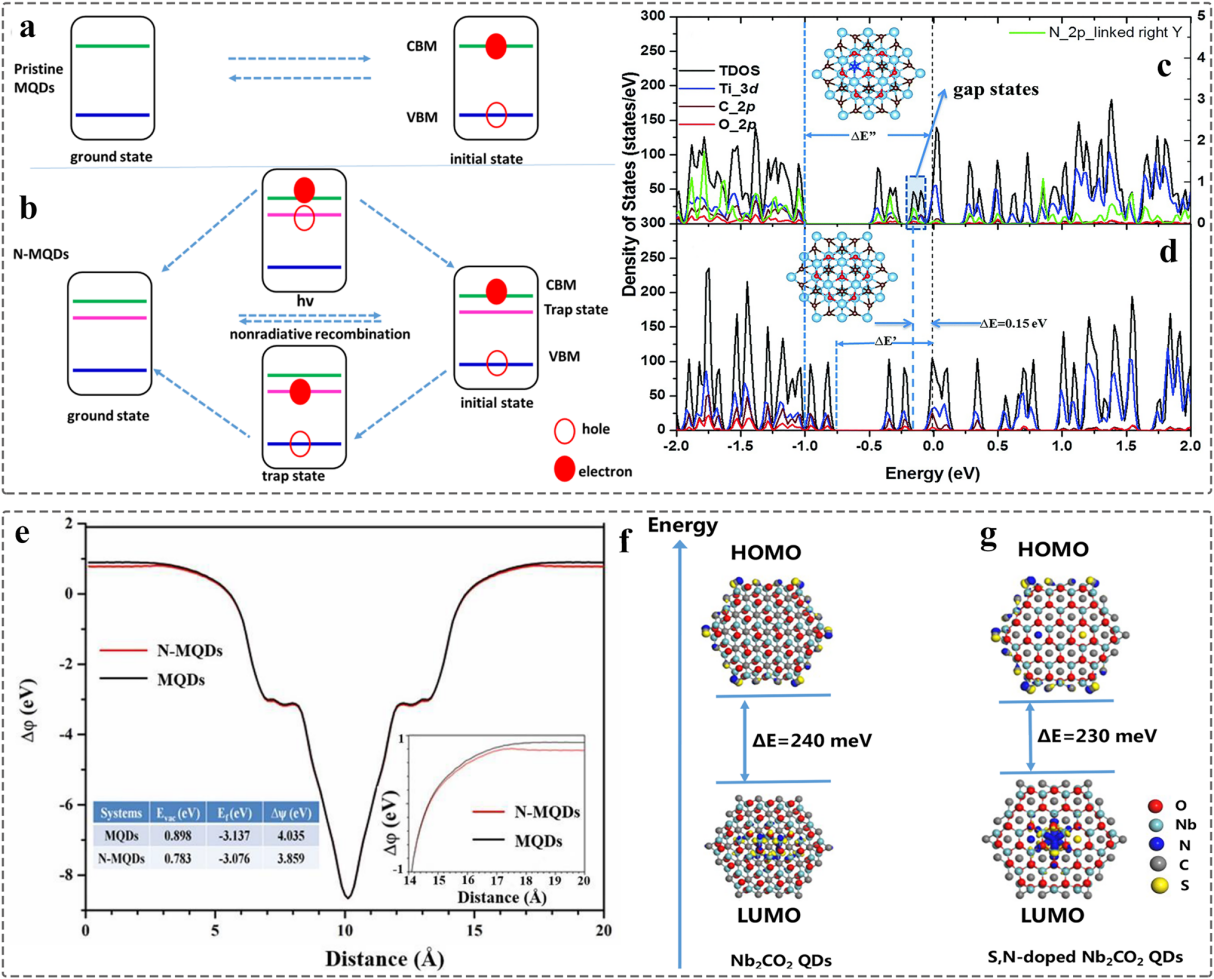
**a-b** Simulation diagram of energy levels and charge transfer processes in the MQDs and N-MQDs; **c-d** DOS calculation of MQDs and N-MQDs; **e** Work function of MQDs and N-MQDs [147]. Copyright @2018, The Royal Society of Chemistry. **f-g** DFT calculation of total and projected DOS of Nb_2_CO_2_ QDs and S, N-doped Nb_2_CO_2_ QDs [86]. Copyright @2020, The Royal Society of Chemistry

In addition, the optical properties can also be further proved by using theoretical simulation. The size effect of MQDs enables the bandgap control, while the introduction of gap states after the surface modified by single/dual atom (N, P, S, etc.) can improve the free carrier lifetimes and promote charge separation (Fig. 16a-b) [147]. Also, the density of states (DOS) demonstrates that the N defect increased the energy gap and work function of MQDs, contributing to fast electron migration (Fig. 16c-e). Simultaneously, the frontier orbitals are simulated by using DFT in the dual atoms modified MQDs (Fig. 16f-g) [86]. Compared to the pristine Nb_2_CO_2_ MQDs, the weak electrons exchange interaction between Highest Occupied Molecular Orbital (HOMO) and Lowest Unoccupied Molecular Orbital (LUMO) of S, N-Nb_2_CO_2_ MQDs contributes to small *△E*, leading to enhance the emission of PL and improve the PLQY of MQDs. Such the combination of experiments and simulation enables clarification of the fluorescence mechanism, and further designing highly efficient photocatalyst.

## Catalytic Applications

**Fig. 17:**
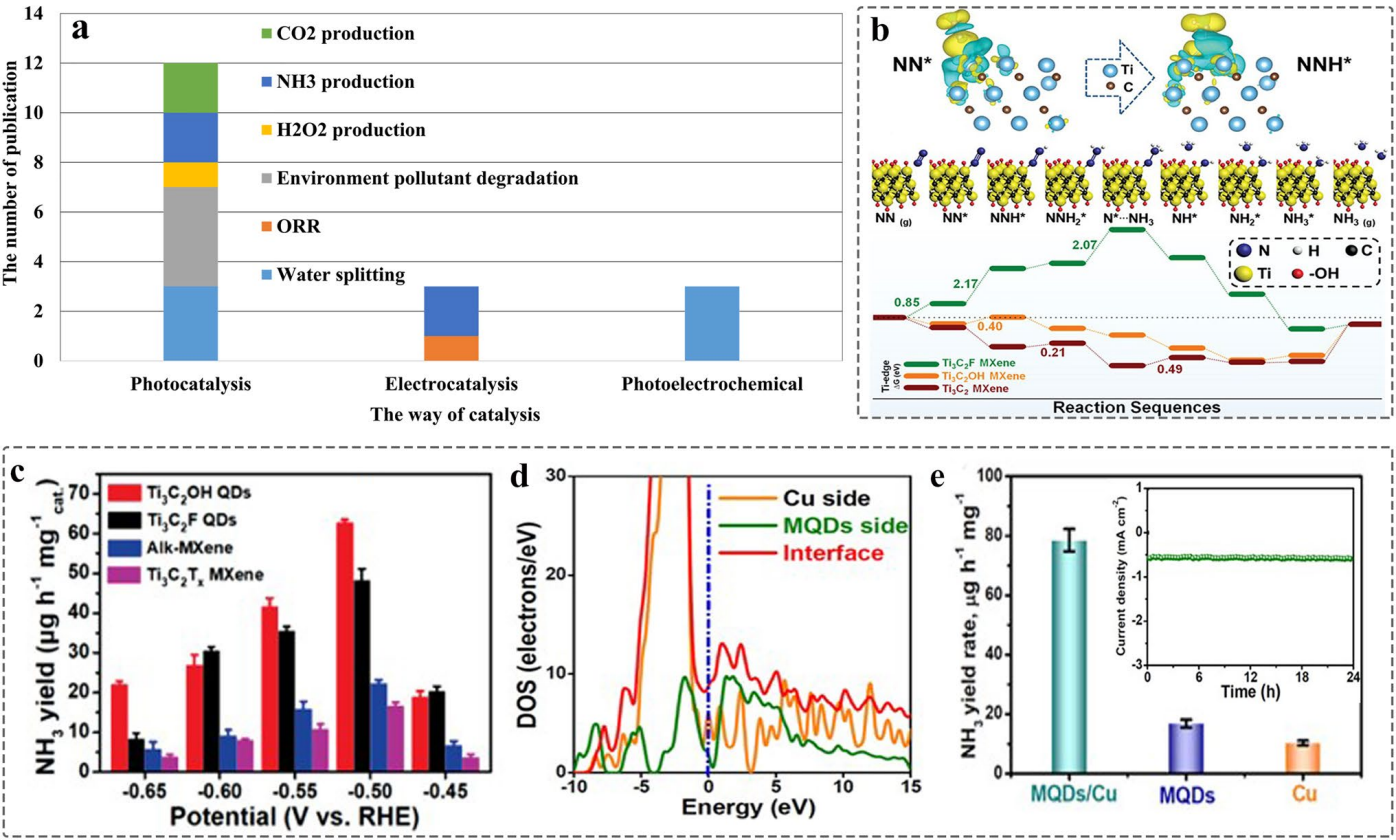
Electrocatalytic performance of MQDs. **a** Number of journal publications on different catalytic aspects (Source: Web of Science). **b** Reaction mechanism of electrocatalytic N_2_ reduction and free energy calculation on the Ti edge of Ti_3_C_2_, Ti_3_C_2_F, Ti_3_C_2_OH MXene from the adsorption of N_2_ to the reduction of NH_3_; **c** The average NH_3_ yield and faradaic efficiency of Ti_3_C_2_OH MQDs at different applied voltages [70]. Copyright @2020, WILEY–VCH. **d** Charge density difference of Ti_3_C_2_ MQDs/Cu; **e** The NH_3_ yield of Cu, MQDs, MQDs/Cu at applied voltage of -0.5 V, inset diagram is chronoamperometry test [79]. Copyright @2022, Zhengzhou University and Wiley

MQDs have been wildly applied to catalysis due to their unique physicochemical properties, especially quantum confinement effect. The way of catalysis can be classified into electrocatalysis, photocatalysis, and photoelectrochemical application. Currently, the application of MQDs mainly focused on photocatalysis due to their larger surface areas, tunable bandgap, and composition. The detailed application is summarized in Fig. 17a, including H_2_ production, oxygen reduction reaction (ORR), pollutant degradation, CO_2_ reduction, NH_3_ production, and H_2_O_2_ production.

### Electrocatalysis

2D MXenes have been used to the field of electrochemical energy conversion, including hydrogen evolution reaction (HER) [10, 103], oxygen evolution reaction (OER) [226, 227], and nitrogen reduction reaction (NRR) [228, 229] due to their highly tunable metal composition and surface functional groups, large specific surface area, good hydrophilicity, and excellent electrical conductivity. However, 2D MXene-derived MQDs are less reported in the field of electrocatalysis. Reducing the size of MXene to less than 10 nm is beneficial to increase abundant edge sites, decrease electron diffusion length, expecting to become the high-performance electrocatalyst candidates.

#### Electrocatalytic Ammonia Synthesis

Ammonia, as an important chemical raw material, plays an indispensable role in the development of agriculture, industry and energy storage [230, 231]. The traditional route of NH_3_ production is Haber–Bosch process, but the conditions of high temperature and pressure increases the operating cost. Over the few years, electrocatalytic NRR has attracted attention due to the mild reaction conditions and abundant resources. However, the strong and stable N≡N bond, the sluggish adsorption of N_2_ and competitive HER side reaction lead to low NRR selectivity and ammonia production rate.

##### The –OH Functional Groups of MQDs as Active Sites

MQDs as an emerging 0D nanomaterials, regarded as a promising NRR electrocatalyst due to their excellent conductivity, abundant surface catalytic active sites, and surface defects. For example, the Ti_3_C_2_OH MQDs were first prepared by agitate-assisting for NRR catalysts. Figure 17b shows the reason why Ti_3_C_2_OH acts as electrocatalyst for NRR with excellent performance: i) N_2_ molecules adsorb at the edge of Ti with positive charge, which accelerates the N≡N bond length from 1.10 to 1.16 Å and activates nitrogen molecules; ii) Compared to both Ti_3_C_2_F_2_ and Ti_3_C_2_, there occur no side reactions of HER when Ti_3_C_2_OH as catalyst to promote NRR, due to the free energy of the rate limiting step is 0.4 eV (the value of HER on the Ti edge of Ti_3_C_2_OH is 0.79 eV) [70]. Therefore, the Ti_3_C_2_OH MQDs with abundant Ti edge and –OH functional groups were prepared. Experiment result shows that the Ti_3_C_2_OH MQDs provides 62.94 µg h^−1^ mg^−1^_cat_ at − 0.50 V. Compared to 2D MXenes, it shows excellent NRR activity due to the offered more active sites, highlighting the unique advantages of size and surface functional groups of MQDs (Fig. 17c).

##### The Interface Design of MQDs-Based Composites

Apart from the surface terminal effect on the adsorption capability of N_2_, the interface engineering is of great significance for promoting the adsorption and activation of N_2_. Moreover, the synergistic catalysis has greater competitive merit compared with pure catalyst, such as enhanced conductivity and hydrophilicity. Consequently, it farcicalities improving the internal electron transport of catalyst or the catalyst-electrolyte interface, thereof further enhancing the catalytic activity, especially in semiconductor catalyst. Therefore, the porous Cu nanosheets with high conductivity were used as support to load Ti_3_C_2_ MQDs for NRR, which were synthesized by chemical reduction. The electron coupling of MQDs-Cu promotes the electrons of MQDs are enriched in the interface (Fig. 17d) contributing to the improvement of electron conductivity [79]. The result shows that Ti_3_C_2_ MQDs/Cu provides 78.5 µg h^−1^ mg^−1^_cat_ at − 0.50 V, better than the pure MQDs, Cu, and other reported analogues (Fig. 17e). The result is superior to the NRR activity of 2D Ti_3_C_2_ nanosheet (4.7 µg h^−1^ mg^−1^_cat_ at − 0.20 V) under same condition [232].

#### Electrocatalytic Water Splitting

HER and NRR are a pair of competing reactions. The –OH functional groups offer favorable free energy with NRR for facilitating the cleavage of N≡N bonds, whereas the 2D Ti_3_C_2_ MXene with –O group has been demonstrated to be HER active sites with the minimum Gibbs free energy (△G_H_*). Demonstrably, to reduce the size of MXenes to less than 10 nm and thereof increase the contact areas between the surface of MQDs and react environment, enable highlighting the unique surface properties. As a result, it will contribute to promote more active sites to participate in the reaction.

**Fig. 18:**
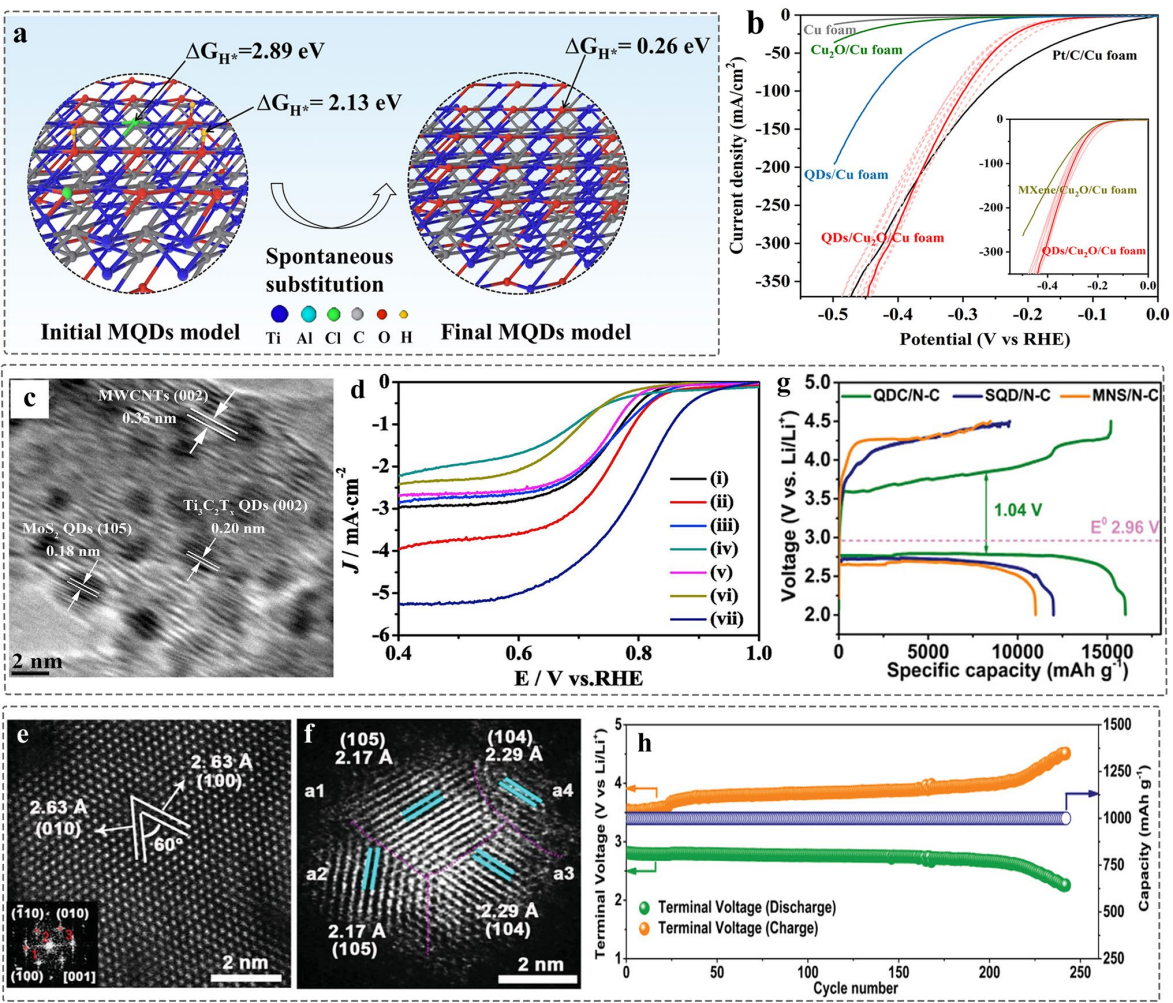
**a** Synthesis of Ti_2_CT_x_ MQDs/Cu_2_O/Cu foam nanocomposite (top), the evolution process of functional groups and Soft X-ray emission spectrum (SXES) image; **b** The LSV image [164]. Copyright @2022, Zhengzhou University and Wiley. **c** TEM image of MoS_2_QDs @Ti_3_C_2_T_x_QDs@MWCNTs and d ORR performance of sample [66]. Copyright @2019, Elsevier. **e** AC-STEM image of Ti_3_C_2_ nanosheet; **f** AC-STEM of Ti_3_C_2_ MQDs; **g** Initial deep discharge–charge curves of the three samples at 200 mA g^−1^; **h** Cycling stability and terminal discharge–charge voltages of Ti_3_C_2_ QDC/N–C electrode at 200 mA g^−1^ [137]. Copyright @2021, Wiley–VCH

The surface –O groups of MQDs as active sites: The support should be introduced into the MQDs based on the high surface energy. For instance, the Ti_2_CT_x_ MQDs/Cu_2_O/Cu foam nanocomposite was prepared by self-assembly method (Fig. 18a), and the Ti_2_CT_x_ MQDs with –Cl, –O, and –OH afford a spontaneous substantial process from –Cl groups to –O groups during HER [164]. The increase in oxygen-containing groups of active sites, the conductivity of the Cu-based support, as well as Cu_2_O nanoparticles as bridges providing the stability, contribute to the derived more excellent HER performance compared with 2D MXenes (Fig. 18b), attributed to the unique merit of MQDs. However, for the △G_H_* of O-terminated MQDs there is still a distance away from the theoretical value. Thus, some means such as the modification of transition metal atoms at the O sites enable weakening the binding energy of O–H bonding.

#### Electrocatalytic OER, ORR, and MOR

##### MQDs as Electronic Conductor

The MQDs also acting as co-catalyst to promote the ORR and methanol oxidation reaction (MOR) are of great significance for improving the commercial application of methanol fuel cells (DMFCs). However, there is only one report on the application MQDs in ORR and MOR. The internal electron transmission of electrocatalyst is enhanced due to the excellent electronic conductivity of MQDs. So, the MoS_2_QDs @Ti_3_C_2_T_x_QDs@MWCNTs nanocomposites show excellent electrochemical performance due to the largest embedded area (Fig. 18c-d) [66]. Therefore, MQDs can be as electrons conductor to promote electrocatalytic reaction.

##### The Defect of MQDs as Active Sites

The presence of surface defects can promote local charge distribution of active sites, control intermediate adsorption behavior. So, it leads to reducing the redox energy barriers during Li_2_O_2_ formation and decomposition, achieving enhanced electrocatalytic kinetics. Wang et al. [137] prepared Ti_3_C_2_ MXene quantum dot clusters (Ti_3_C_2_ QDC) with rich grain boundaries and edge defects through hydrothermal thermal-shearing reaction method. The defects were firstly characterized by using AC-STEM. Compared to perfect crystal of 2D MXene (Fig. 18e), the MQDs with defects show considerable grain boundary and unsaturated edge sites (Fig. 18f). Thus, MQDs exhibit better Li-O_2_ catalytic activity with high capacity and cycling stability compared with 2D MXene (Fig. 18g-h). Such atomic-scale clarification of the catalytic reaction mechanism with multiple defect-dominated MQDs provides a strategy for designing highly active catalysts.

### Photocatalysis

Photocatalysis is a redox reaction based on the photocatalyst surface under visible light, which has been regarded as one of the potential green technologies to solve the problems of energy shortage and environmental problems [233]. Broadening the spectral response range, increasing the carrier concentration, and reducing the recombination of photogenerated carriers are the keys to design efficient and stable photocatalysts [234]. MQDs have been considered as ideal co-catalysts due to their excellent conductivity, large surface areas, tunable bandgap, and strong quantum conferment effect, which has been successfully applied to photocatalytic hydrogen production, pollutant degradation (e.g., NO, heavy metal), CO_2_ reduction, NH_3_ production, and H_2_O_2_ production.

#### Photocatalytic Water Splitting

Hydrogen (H_2_) has been considered as one of the ideal fuel due to low densities, high calorific value, abundant raw materials, and non-polluting combustion products [235, 236]. The light-driven water splitting to obtain H_2_ is a promising conversion technology [237]. Over the past few years, transition metal oxides, transition chalcogenides, and organic semiconductors have been developed for photocatalytic hydrogen production due to their suitable energy band structures. The high-performance photocatalysts need to meet the following conditions: (i) the semiconductor possesses broad light adsorption range to generate more photogenerated carriers; (ii) the adequate energy band structure meets the thermodynamic requirements of water splitting; (iii) photogenerated electron and holes can be effectively separated. Therefore, designing the structure of semiconductors is of great significance for realizing an efficient photocatalytic water splitting reaction.

##### MQDs as Photoelectrons Acceptor

**Fig. 19:**
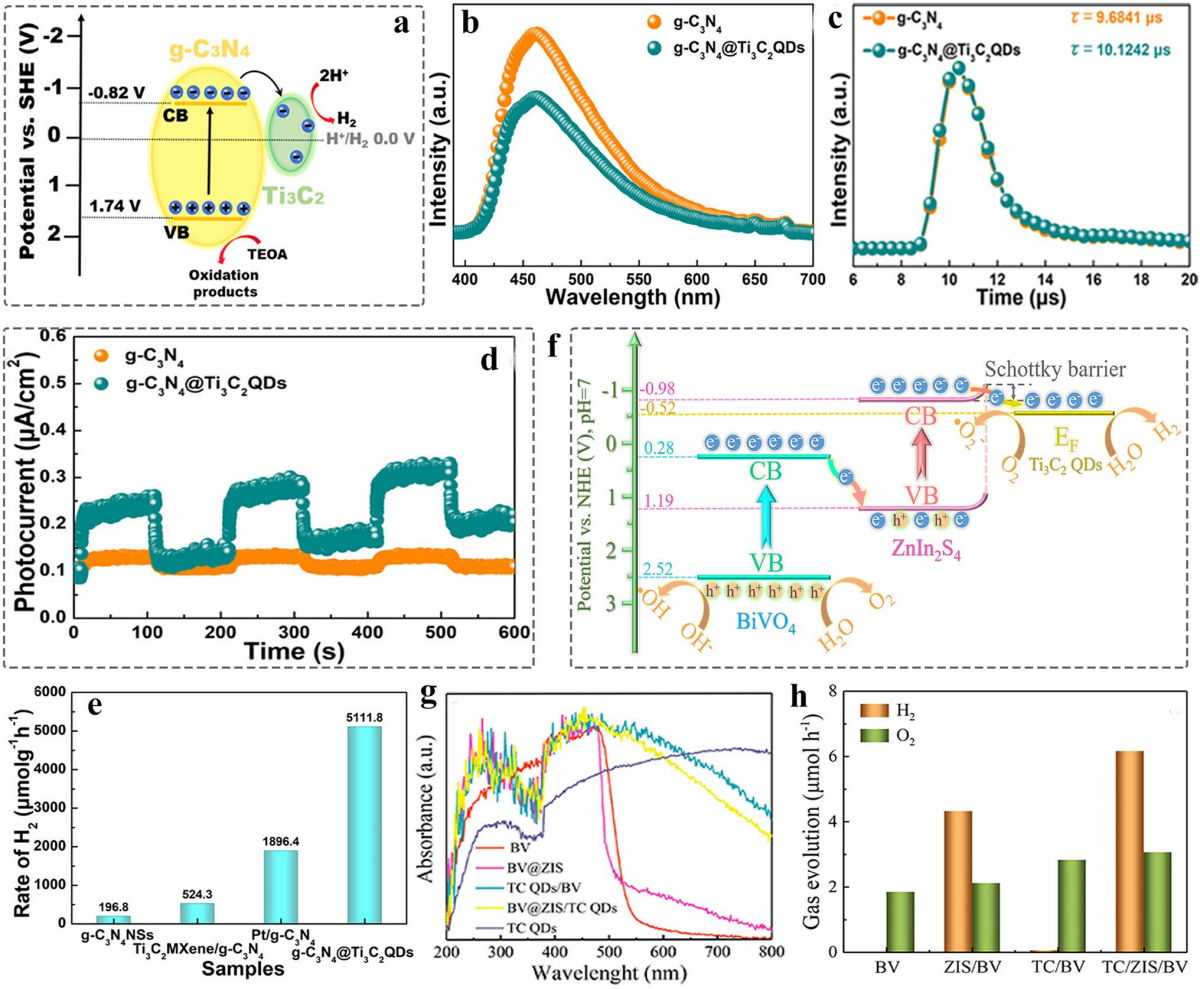
Photocatalytic water splitting performances of MQDs-based heterostructure. **a** Schematic react mechanism of g-C_3_N_4_@Ti_3_C_2_ QD; **b** Steady photoluminescence spectra of g-C_3_N_4,_ MQDs@g-C_3_N_4_; **c** Time-resolved fluorescence decay spectra under the 325 nm excitation wavelength; **d** The transient photocurrent response; **e** Photocatalytic HER rate plot of catalyst [160]. Copyright @2019, American Chemical Society. **f** Schematic photocatalytic mechanism of BV@ZIS/TC QDs; **g** UV–visible diffuse reflectance spectra of BV@ZIS/TC QDs and control sample; **h** Photocatalytic gas production of BV@ZIS/TC QDs and control sample [68]. Copyright @2020, Elsevier

Compared to the traditional TiO_2_ photocatalyst, the layered g-C_3_N_4_ possesses narrow band gap (2.7 eV) and visible light activity, whereas low light response ranges from 450 to 460 nm, and high electron–hole pairs recombination rate results in poor photocatalyst performance [238]. Thus, the Ti_3_C_2_ QDs with excellent conductivity were co-catalyst to improve it [160]. As shown in Fig. 19a, the conduction band of MQDs with abundant catalytic active sites is more positive than that of g-C_3_N_4_, which can capture electrons to facilitate surface redox reactions [160]. Such behavior of low recombination capability of photogenerated electron-holes pairs induced a low PL intensity. The time-resolved fluorescence decay spectra show that lifetime of carriers increased to 10.1242 μs, further demonstrating the result (Fig. 19b-c). As a result, the photocurrent intensity of Ti_3_C_2_ QDs/g-C_3_N_4_ is higher than 2D g-C_3_N_4_ nanosheets (Fig. 19d). Therefore, MQDs as electron acceptors to capture quickly the photogenerated electrons, facilitating the efficient carrier transfer. Finally, the H_2_ production rate of Ti_3_C_2_ QDs/g-C_3_N_4_ (5111.8 μmol g^−1^ h^−1^) is far higher than g-C_3_N_4_ (196.8 μmol g^−1^ h^−1^), Pt/g-C_3_N_4_ (1896.4 μmol g^−1^ h^−1^) and 2D MXene/g-C_3_N_4_ (524.3 μmol g^−1^ h^−1^) at the same conditions (Fig. 19e), highlighting the merit of MQDs.

##### Designing the Z-scheme structure of MQDs-based photocatalyst

For designing favorable energy band structure, the instruction of Schottky junction to increase the extraction of photoelectrons is an effective strategy. The MQDs as co-catalyst to construct Z-scheme structure of BiVO_4_@ZnIn_2_S_4_/Ti_3_C_2_ (BV@ZIS/TC QDs) [68], forming the Schottky barrier at the interface. As described in Fig. 19f, the photogenerated electrons of conduction band in BV were injected into the valance band of ZIS. Then more abundant photogenerated electrons were injected into the conduction band of MQD, achieving an effective carrier separation. The presence of MQDs broadens the light response range from visible light to near-infrared region, helping to produce more photoelectrons, and thereof promoting efficient H_2_ and O_2_ production rates (Fig. 19g-h). Also, the Ti_3_C_2_-QDs/ZnIn_2_S_4_/Ti(IV) heterojunction photocatalyst was prepared for improving hydrogen production performance [78].

#### *Photocatalytic CO*_*2*_* Reduction*

The goal of carbon neutrality is an inevitable choice based on the high-quality development of China's economy and society. Photocatalytic CO_2_ reduction is an efficient technology to achieve a low carbon economy [239]. Furthermore, methanol, the reduction product of CO_2_, can also be used as a fuel. It is worth noting that the photocatalytic CO_2_ reduction activity is related to the light adsorption ability, photogenerated carriers’ separation efficiency, and the activation ability of photocatalysts for CO_2_ molecules [240]. Compared to 2D MXene, MQDs offer controllable bandgap due to size effect, abundant unsaturated sites to adsorb CO_2_ molecules, contributing to the selectivity of reaction products.

**Fig. 20:**
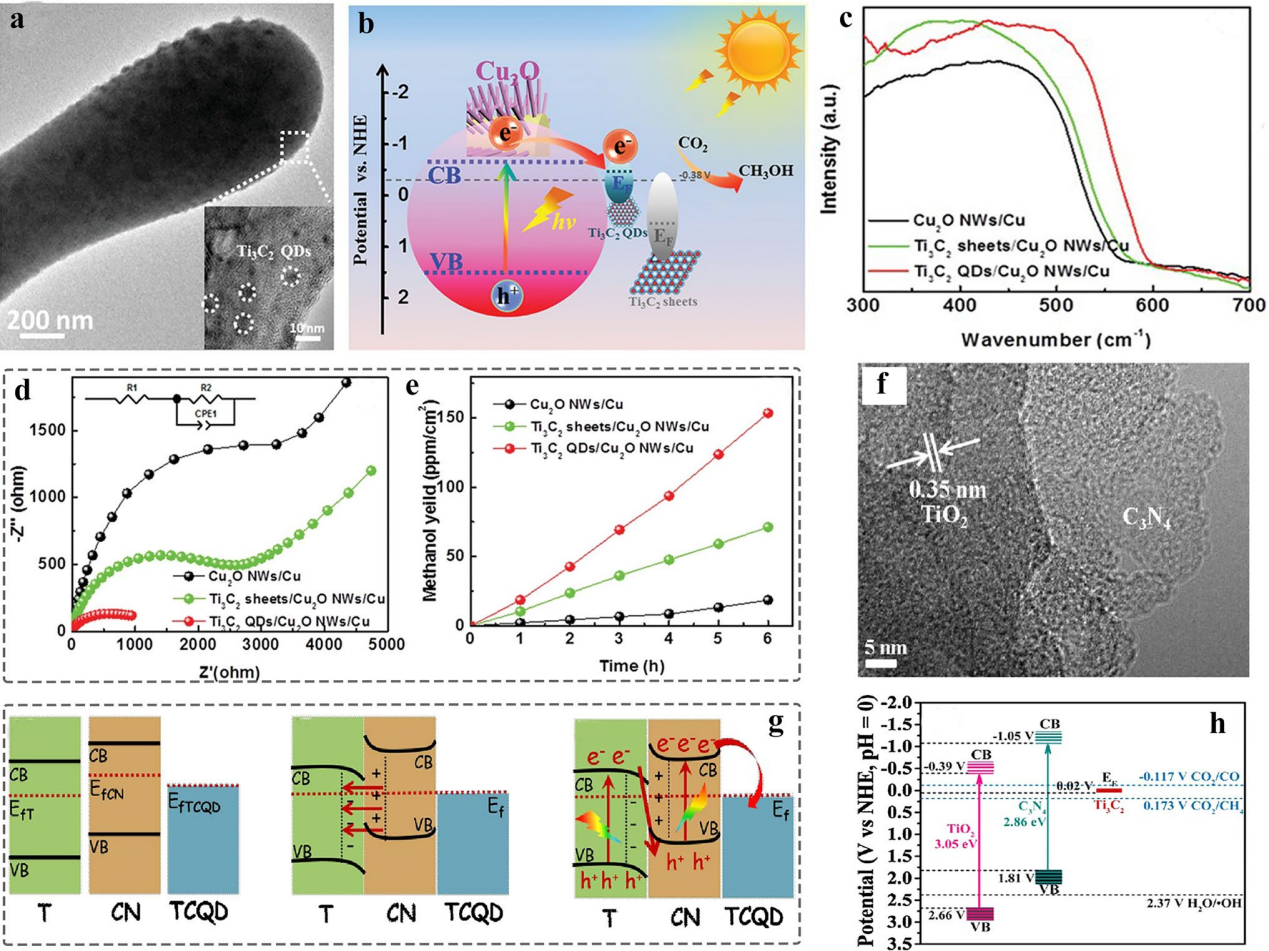
Photocatalytic CO_2_ reduction performances of MQDs-based photocatalysts. **a** TEM image of Ti_3_C_2_ MQDs/Cu_2_O/Cu; **b** Photocatalytic reaction mechanism of Ti_3_C_2_ MQDs/Cu_2_O/Cu; **c** UV–Vis diffuse reflectance spectra (DRS) of samples; **d** Nyquist plots of samples; **e** Methanol yield of samples [67]. Copyright @2019, WILEY–VCH. **f** TEM of TiO_2_/C_3_N_4_ with core–shell; **g** S-scheme heterojunction before and after contact, and light irradiation; **h** Schematic diagram of structure of TiO_2_/C_3_N_4_/Ti_3_C_2_ MQDs [159]. Copyright @2020, Elsevier

Designing the S-scheme structure of MQDs-based photocatalyst: The semiconductor Cu_2_O has narrow bandgap of 2.2 eV, and the more negative potential of conduction band than reduction potential of CO_2_, which is unfavorable photocatalytic CO_2_ reduction. Therefore, it is important for designing the energy band structure to suppress recombination of the carriers to promote effective reaction. In 2019 [67], Zeng et al. constructed S-scheme heterojunction by electrostatic self-assembly (Fig. 20a). The energy band structure shows that the photogenerated electrons of conduction band (CB) in Cu_2_O transfer into CB of MQDs due to the Fermi level of MQDs lower than CB of Cu_2_O, avoiding the recombination of carriers (Fig. 20b). The MQDs as co-catalyst to increase light adsorption capability of Cu_2_O nanowires, and reduce the charge transfer resistance (Fig. 20c-d). Therefore, compared to the 2D MXene, the Ti_3_C_2_ MQDs/Cu_2_O/Cu contributes to high CH_3_OH yield (Fig. 20e). Furthermore, since designing core–shell MQDs-coupled nanosheet (TiO_2_/C_3_N_4_) with S-scheme heterojunction (Fig. 20f), the formation of band edge bending and internal electric field at the interface enables balancing the Fermi level (Fig. 20g). Thus, the photoelectrons are accumulated on the conduction band of MQDs to achieve an efficient redox reaction for improving the selectivity of products (Fig. 20h). Such result shows the 0D MQDs as electron acceptor to accelerate spatial migration of electrons compared with 2D MXene, which is an effective strategy to control the type and amount of heterointerface [159].

#### *Photocatalytic NH*_*3*_* and H*_*2*_*O*_*2*_* Production*

Compared to the traditional complex Haber–Bosch process, photocatalytic NH_3_ production is a feasible method due to economy, environmentally friendly, and facile conditions [241]. There are three conditions to achieve high ammonia yield: (i) the weak bond energy of N≡N; (ii) the broad-spectrum response range for increasing the concentration of carrier; (iii) the efficient carrier separation rate. Among them, the recombination of photogenerated carries is a main obstacle to hinder the activation of N_2_ molecules. The MQDs with quantum confinement effect and good conductivity can promote the bandgap control and effective charge transfer.

##### Designing the Band Structure of MQDs-Based Photocatalyst

**Fig. 21:**
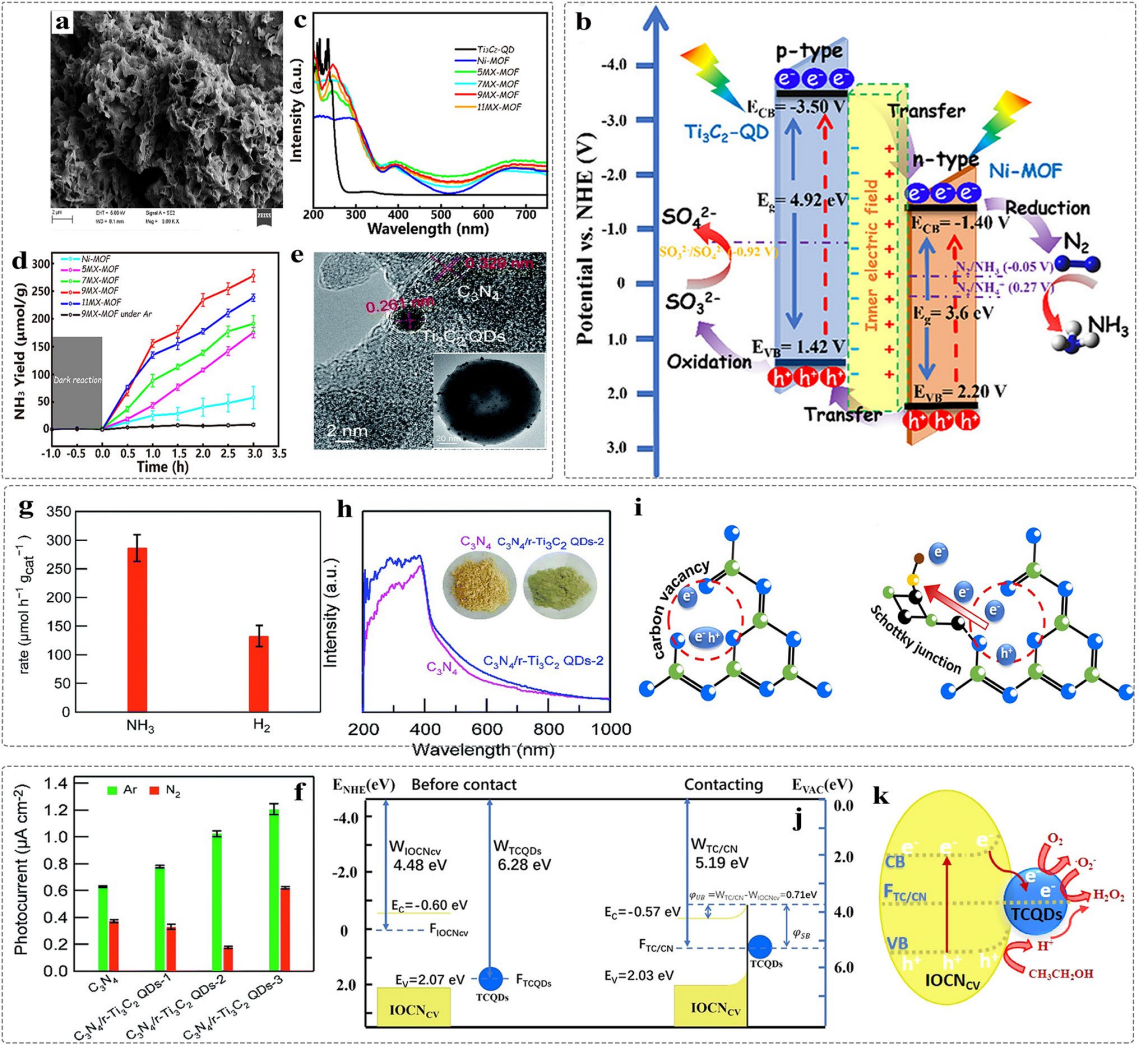
Photocatalytic performances of NH_3_, H_2_O_2_ production. **a** SEM image of Ti_3_C_2_ MQDs/Ni-MOF; **b** Energy band structure of Ti_3_C_2_ MQDs/Ni-MOF; **c** UV–Vis diffuse reflectance spectra of Ti_3_C_2_ MQDs, Ni-MOF, Ti_3_C_2_ MQDs/Ni-MOF nanocomposites with different loads of MQDs; **d** The NH_3_ yield of Ti_3_C_2_ MQDs/Ni-MOF [69]. Copyright @2020, American Chemical Society. **e** TEM image of C_3_N_4_/r-Ti_3_C_2_ MQDs Schematic diagram of photocatalytic N_2_ fixation; **f** Photocurrent test in Ar and N_2_ of samples; **g** UV–Vis DRS spectra of C_3_N_4_, C_3_N_4_/r-Ti_3_C_2_ MQDs; **h** Photocatalyst NH_3_ production rate and side reaction of H_2_ production [76]. Copyright @2022, The Royal Society of Chemistry. **i** Transfer of photogenerated electrons and holes near the carbon vacancies of C_3_N_4_ and C_3_N_4_/ r-Ti_3_C_2_ MQDs; **j** The energy band structure of C_3_N_4_/ r-Ti_3_C_2_ MQDs; **k** The photocatalytic reaction mechanism diagram of C_3_N_4_/r-Ti_3_C_2_ MQDs [73]. Copyright @2021, American Chemical Society

The MQDs as co-catalyst of Ni-MOF were anchored on the surface of 2D nanosheets Ni-MOF (Fig. 21a) [69]. The energy theory shows the excellent energy level matching hinders the recombination of photogenerated carrier under the simulated light irradiation, and more photogenerated electrons were accumulated on the CB of Ni-MOF (Fig. 21b), Compared to pure Ni-MOF photocatalyst, the MQDs help to enhance the light adsorption ability, increasing the carrier concentration (Fig. 21c). Leading to high-yield ammonia production (Fig. 21d).

##### Constructing Defects of MQDs for Reactant Adsorption

The defect sites with lower binding energy facilitate to promote the adsorption of N_2_ molecules. In 2022, Chang et al. [76] engineered a photocatalyst with Schottky junction and defects. The Ti_3_C_2_ MQDs with amount of oxygen vacancies (OV) and Ti^3+^ sites were anchored on the surface of mesoporous C_3_N_4_ hollow nanosphere (Fig. 21e). Such unique design has two advantages: 1) the vacancy defects are beneficial to increase the adsorption and activation of N_2_ molecules; 2) the quantum confinement effect of MQDs promotes the light adsorption intensity of C_3_N_4_ (Fig. 21f-g), leading to high carrier concentration and excellent photocatalytic activity (Fig. 21h). This is of great significance for guiding the design of MQDs-based photocatalysts. In addition, the rich carbon vacancies of MQDs as bridge site induced the bonding MQDs with g‑C_3_N_4_, forming Schottky junction at the interface. Thus, it increases the work function of energy band, promoting the photoexcited carrier separation [73]. Such optimized photocatalytic activity of Ti_3_C_2_ MQDs/g‑C_3_N_4_ achieves a high yield of 560.7 μmol L^−1^ h^−1^ (Fig. 21i-k).

#### Photocatalytic Pollutant Degradation

**Fig. 22:**
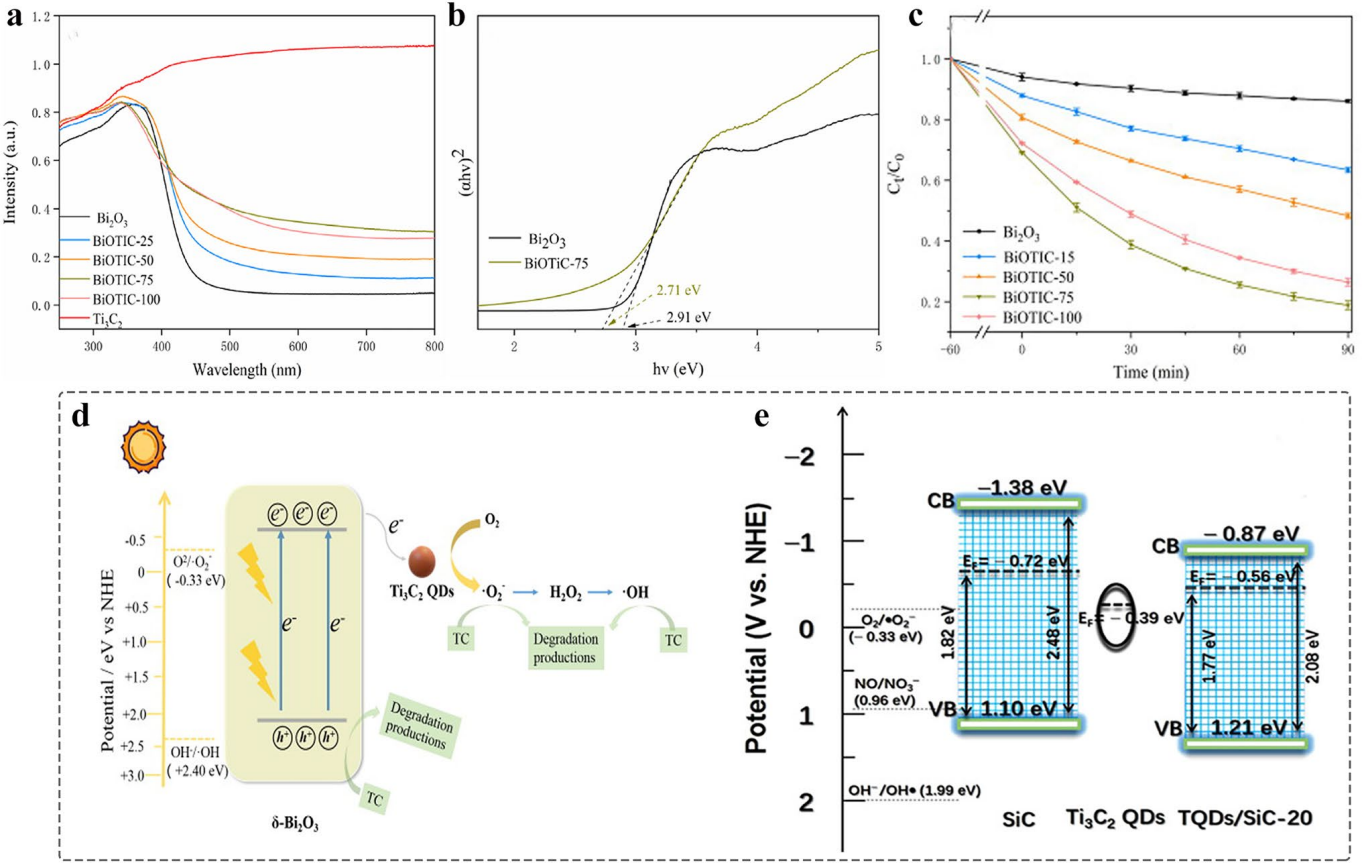
Photocatalytic pollutant degradation performances of MQDs-based heterojunction. **a** UV–Vis diffuse reflectance spectra (DRS) of samples; **b** Calculation of band gap of Bi_2_O_3_, BiOTIC-75; **c** Photocatalytic reaction mechanism illustration of BiOTIC-75; **d** Photocatalytic degradation of TC by Bi_2_O_3_, Ti_3_C_2_ MQDs/Bi_2_O_3_ with different content of MQDs [75]. Copyright @2021, Elsevier. **e** Energy band structure of MQDs/SiC, SiC, MQDs [71]. Copyright @2020, American Chemical Society

The introduction of MQDs could improve the photocatalytic activity of photocatalysts, which is also reflected in the field of wastewater treatment and purification of air pollutants. In 2021, the Ti_3_C_2_ MQDs acted as co-catalyst of Bi_2_O_3_ (BiO/TiC) to boost photocatalytic tetracycline (TC) degradation in water [75]. Compared to pure Bi_2_O_3_, the MQDs/Bi_2_O_3_ nanocomposites show a broad visible light adsorption due to excellent metal conductivity of the MQDs (Fig. 22a). Furthermore, the band gap is reduced from 2.91 to 2.71 eV due to the quantum confinement effect of the MQDs (Fig. 22b). It is favorable to promote the photogenerated carrier separation, and the photoexcited electrons were thereof accumulated on the CB of MQDs (Fig. 22c). Thus, it leads to excellent photocatalytic tetracycline degradation effect (Fig. 22d). Compared to pure Bi_2_O_3_ photocatalyst, the MQDs as co-catalyst shows enhanced degradation efficiency by 5.85 times, and far surpass precious metal Au, Pt nanoparticles co-catalysts (1.75, 2.18 times). Likewise, such strategy of introducing of MQDs to balance the interface contact energy levels further leads to separation of photogenerated electron–hole pairs, which has been used to prepare other types of heterostructures to facilitate pollutant degradation. For example, the Ti_3_C_2_ MQDs have also been used as co-catalyst to prepare Ti_3_C_2_ MQDs/SiC nanocomposite [71] (Fig. 22e), all-solid-state WO_3_/TQDs/In_2_S_3_ Z-scheme heterostructure [74] and Ni@MQDs [77] nanocomposite photocatalyst, achieving the goal of photocatalytic removal of NO purification and pollutants in water.

### Photoelectrocatalysis

Compared to photocatalysis and electrocatalysis, photoelectrocatalysis is a stronger method to promote electrochemical reaction, which combines the advantages of both the catalysis methods [242]. Currently, MQDs have been used to research photoelectrochemical water splitting, and the progress was made.

#### MQDs as Photoanode

**Fig. 23:**
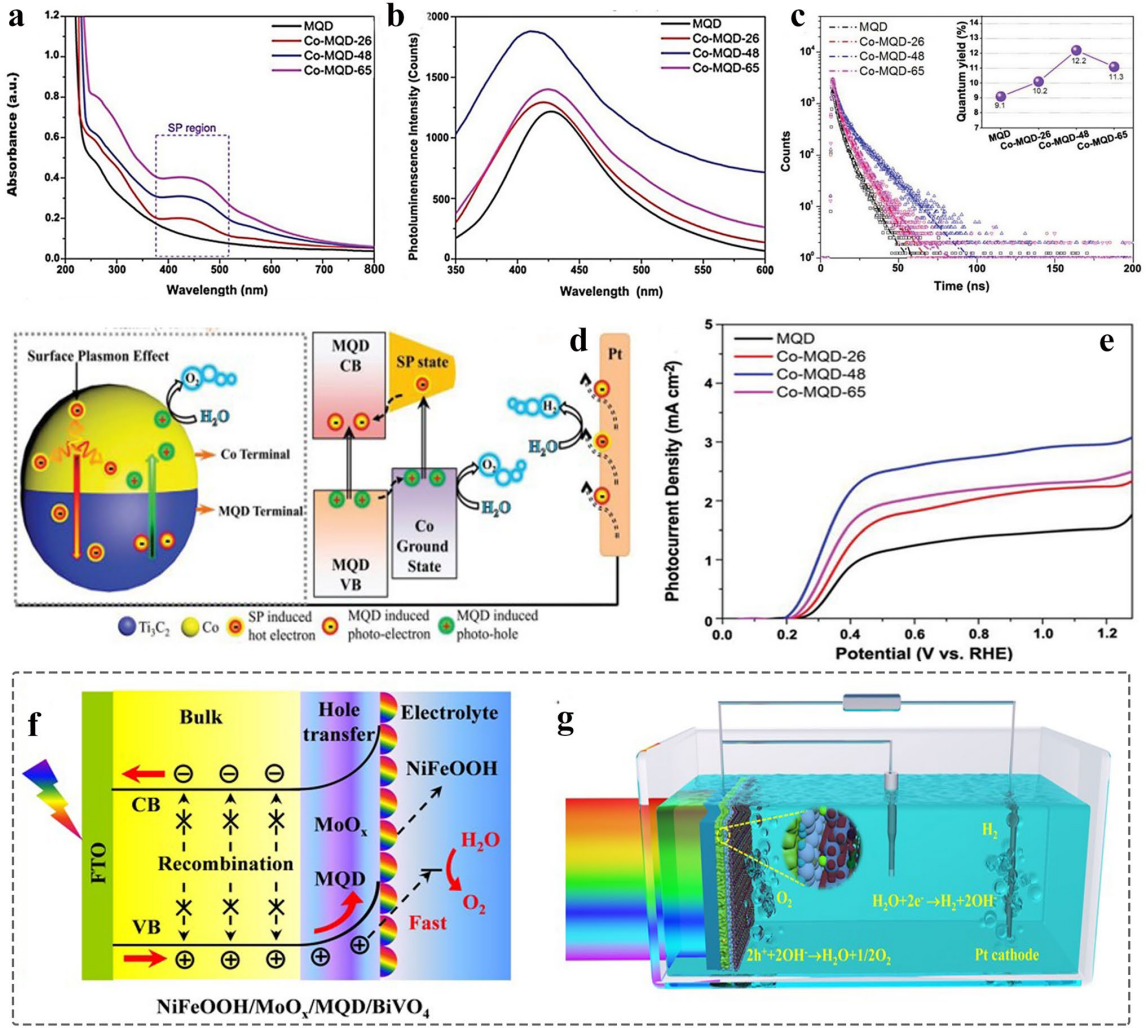
The photoelectrocatalytic performances of MQDs as co-catalyst. **a** UV–Vis adsorption spectra of Ti_3_C_2_ MQDs, Co-MQDs with different rates of Co/Ti; **b** Photoelectric conversion efficiency of Ti_3_C_2_ MQDs, Co-MQDs with different rates of Co/Ti; **c** Time-resolved photoluminescence (TRPL) spectra; **d** Photoelectrocatalytic water splitting mechanism under light irradiation; **e** Photocurrent test of Ti_3_C_2_ MQDs, Co-MQDs with different rates of Co/Ti [72]. Copyright @2020, WILEY–VCH. **f** Schematic illustration of the charge transfer process for NiFeOOH/MoOx/MQD/BiVO_4_ photoanodes; **g** Photoelectrochemical water splitting device [161]. Copyright @2022, Wiley–VCH

In 2020, the Janus-structure Co-Ti_3_C_2_ MQDs were prepared by thermal-cutting method, which was used as photoanode for water oxidation [72]. The Co nanoparticles were coupled with Ti_3_C_2_ MQDs forms Schottky junctions that increase the extraction of photogenerated carriers. Compared to pure MQDs, the introduction of Co triggers the concomitant surface plasmon effects, thus showing that enhanced light adsorption (200–600 nm) and additional adsorption peak (380–520 nm) (Fig. 23a), attributed to increase the amount of photogenerated carrier. Furthermore, the enhanced steady-state PL intensity indicates good carrier migration, while MQDs with high loadings as carrier recombination center reduces quantum yield, resulting in low PL intensity (Fig. 23b). As shown in Fig. 23c, the time-resolved photoluminescence (TRPL) spectra show that Co coupled with MQDs can increase the average carrier lifetime, indicating low carrier recombination rates, which is consistent with PL spectra result. Such Schottky hierarchical structure contributes to promote photogenerated electrons were extracted from the CB of Co to the CB of MQDs, promoting high photoelectrochemical water oxidation performance (Fig. 23d-e).

#### MQDs as Hole Transfer Layers

The stability and charge separation of photoanode is important for improving photoelectrochemical (PEC) water oxidation activity. The MQDs can be used as co-catalyst to construct the hole transfer layers of MoOx/MQDs for delaying carrier recombination (Fig. 23f), enhancing the light response range, leading to high activity and stability of PEC reaction (Fig. 23g) [161]. Such strategy broads the high-performance full-spectrum photoelectrochemical water splitting [157].

## Summary and Perspectives

Since in 2017, 2D MXene-derived 0D MQDs have made great progress in proceeding into a variety of catalysis due to their improved and optimal physicochemical properties. Research has shown that the surface metal ions, functional groups, and abundant edge sites of MQDs can act as active sites to adsorb and activate the gas molecules, and the MQDs are also treated as co-catalyst to promote an efficient charge separation and enhance charge transfer kinetics. Consequently, it reveals that MQDs have promising potential in the field of catalysis. However, there are some problems upon MQDs remain to be resolved, such as low yield, easy aggregation, poor stability in preparation, and difficulty to precisely control the surface chemistry. Such disadvantages are not beneficial to the comprehensive development of MQDs in the field of catalysis.

In this review, we update the recent research progress in catalysis, including the research status, involved with the synthesis of pure MQDs and functional MQDs, and relevant characterization techniques, in order to design high-performance MQDs-based catalysts. Meanwhile, the challenges must be confronted on the basis of synthesis, formation mechanism, wide application, surface defects, and advanced characterization techniques of MQDs.

### Synthesis Condition and Formation Mechanism of MQDs

Currently, the synthesis methods of MQDs are based on single-layer or multilayer MXene nanosheets as precursors, and MXene was prepared by F-containing etchant (e.g., HF, LiF + HCl). Many studies show that F terminal is unfavorable to electrochemical reaction process. Therefore, the preparation of F-free MQDs should be fully considered. Furthermore, since most of MQDs was prepared by hydrothermal strategy, the surface suffers from an easy oxidation in this process. So, it results in the difficulty to obtain high-purity MQDs. For such the irregularity of synthesis method, and the influence of impurities in the product, the formation mechanism of MQDs is not well clarified; it poses a challenge to precisely control the growth of MQDs. Also, for preparing high-quality MQDs (the desired structure, shape, size, distribution of functional groups, and types of surface defects), it is necessary to systematically study the composition of MQDs and various reaction conditions (solvent, temperature, reaction time, power, and pH) effect on the performance of MQDs. Moreover, the introduction of in situ characterization techniques can contribute to elucidate the formation mechanism and nanostructure.

Furthermore, Bottom-up method has been used to synthesize other QDs due to the advantages of adjustable surface chemistry, morphology and size, and the precise controlling of synthesis conditions. Thus, such method can be considered for synthesizing MQDs with excellent crystallinity, monodisperses, and stability.

### Synthesis of Novel MQDs

There are many kinds of MXenes reported so far experimentally and in theoretical prediction, while the application focuses mainly on Ti_3_C_2_ MQDs. The structure and properties of MQDs are correlated with the type, quantity, and arrangement of metallic elements, which affect the energy band structure. Therefore, exploring the performance of different types of MQDs in catalysis not only is beneficial to building the relationship between the composition, structure, and properties of MQDs, but also to searching for low-cost, high-activity, and high-stability catalysts.

### Role of MQDs in Electrochemical Reaction

Like other inorganic and organic QDs, MQDs tend to agglomerate due to surface effects during catalytic reactions. The choices of the support, and the study of the interaction between MQDs and the support, especially the chemical properties at the interface are of great significance for improving the electrochemical performance. Furthermore, apart from the performance of MQDs as catalysts, MQDs can also be considered as the support of nanomaterials such as single atoms and metal nanoparticles, benefited from the abundant functional groups on the surface of MQDs that can directly serve as anchoring sites, avoiding additional surface modification steps such as carbon-based materials.

### Novel Application of MQDs

On the basis of the progresses, the application of MQDs in electrocatalysis is undoubtedly in the infancy stage. Especially, there are still lots of space in the field of HER and OER. Currently, some fluorescent QDs such as carbon dots (CDs), graphene QDs (GQDs), carbon QDs (CQDs) have been used to apply on catalysis. Their synthetic methods, surface-modified strategies, the roles recognition of catalytic reaction, characteristic techniques, and the exploration of catalytic mechanism have been widely investigated. As analogy, compared to carbon-based QDs, MQDs have controllable composition, complex internal structure. Thus, the exploration of MQDs for catalytic application can learn from the research style of carbon-based QDs, however, the challenges remain. For further guiding the design of high-performance catalysts, it is necessary to construct some theoretical models to predict the effect of surface state and external environment (temperature, pressure, and illumination) on the catalytic activity.

### Surface Defects of MQDs

Defective sites with low binding energy are commonly considered as key sites for catalytic activity. They not only act as adsorption sites of reactants, but as the coupling site of metal nanoparticles. MXene QDs with surface defects can be obtained by atomic doping, electrochemical reduction, reducing agents, etc. The introduction of surface defects facilitates to improve the electronic structure of the active sites around the MQDs. So, the electron transfer process and the adsorption/desorption behavior of reactants can be well controlled during the electrocatalytic reaction. In addition, when MQDs are used as photocatalysts, the controllable size of MQDs enables a unique band gap structure, beneficial to photocatalytic, and photoelectrocatalytic reactions.

Generally, the photocatalytic reaction performance is related to the separation efficiency of photogenerated carriers and light absorption range. Constructing surface defect is an effective strategy. Furthermore, studies have shown that the passivation of surface defects induced by amino groups facilitates to improve the PL properties of MQDs, resulting in bright blue fluorescence and enhanced fluorescence lifetime [140]. The doping includes both non-metallic elements such as N, P, and S, and transition metal elements such as Ni, Co, and Cu. As a result, a variety of surface and subsurface defects are produced. Controlling the surface oxygen content of MQDs can also achieve surface defects, unfortunately yet to be realized. The effect of surface defects on the PL properties remains to be revealed.

### In Situ Characterization Techniques

The atomic structure of MQDs was observed under ultra-high-resolution electron microscopy to study the distribution of defects, the arrangement of atoms, and the special sample-supporting mesh was used to visualize the dynamic evolution of catalysts, which is something expected. Moreover, the advanced in situ techniques such as Raman, synchrotron radiation, FTIR expect to elucidate the nanostructure and formation mechanism of MQDs and MQDs-based catalyst, and contribute to reveal the structural evolution in catalytic process, toward designing high-performance MQDs-based catalysts. It is worth noting that the surface reconstruction of MQDs-based nanocomposites during the electrochemical reaction can be intuitively observed by using in situ characterization. It is thereof beneficial to clarify the real catalytic active sites, and confirm the morphology and structural changes after the reaction. On the basis of such foundation, it is expected to construct optimized performance catalysts.

In general, an increasing number of investigations concentrate on the synthesis, modification, application of MQDs in the past five years. Therefore, our review provides new insights into the recognition of MQDs, and illustrates recent progress in catalysis. As a result, it will provide guidance and reference for the preparation of novel MQDs and the design of high-performance MQDs-based catalysts.

## References

[1] Winderlich R (1948). Jons Jakob Berzelius. J. Chem. Educ..

[2] Morachevskii AG (2004). Jons Jakob Berzelius (to 225th anniversary of his birthday). Russ. J. Appl. Chem..

[3] Rupert FF (1910). The solid hydrates of ammonia II. J. Am. Chem. Soc..

[4] Shi MM, Bao D, Wulan BR, Li YH, Zhang YF (2017). Au sub-nanoclusters on TiO_2_ toward highly efficient and selective electrocatalyst for N_2_ conversion to NH_3_ at ambient conditions. Adv. Mater..

[5] Ling C, Bai X, Ouyang Y, Du A, Wang J (2018). Single molybdenum atom anchored on N-doped carbon as a promising electrocatalyst for nitrogen reduction into ammonia at ambient conditions. J. Phys. Chem. C.

[6] Arquer FPG, Talapin DV, Klimov VI, Arakawa Y, Bayer M (2021). Semiconductor quantum dots: technological progress and future challenges. Science.

[7] Naguib M, Kurtoglu M, Presser V, Lu J, Niu J (2011). Two-dimensional nanocrystals produced by exfoliation of Ti_3_AlC_2_. Adv. Mater..

[8] Huang D, Xie Y, Lu D, Wang Z, Wang J (2019). Demonstration of a white laser with V_2_C MXene-based quantum dots. Adv. Mater..

[9] Xiong J, Pan L, Wang H, Du F, Chen Y (2018). Synergistically enhanced lithium storage performance based on titanium carbide nanosheets (MXene) backbone and SnO_2_ quantum dots. Electrochim. Acta.

[10] Yuan W, Cheng L, An Y, Wu H, Yao N (2018). MXene nanofibers as highly active catalysts for hydrogen evolution reaction. ACS Sustain. Chem. Eng..

[11] Therrien AJ, Hensley AJR, Marcinkowski MD, Zhang R, Lucci FR (2018). An atomic-scale view of single-site Pt catalysis for low-temperature CO oxidation. Nat. Catal..

[12] Xu H, Shang H, Wang C, Du Y (2020). Ultrafine Pt-based nanowires for advanced catalysis. Adv. Funct. Mater..

[13] Pagliaro M, Campestrini S, Ciriminna R (2005). Ru-based oxidation catalysis. Chem. Soc. Rev..

[14] Singha S, Serrano E, Mondal S, Daniliuc CG, Glorius F (2020). Diastereodivergent synthesis of enantioenriched α, β-disubstituted γ-butyrolactones via cooperative N-heterocyclic carbene and Ir catalysis. Nat. Catal..

[15] Zhu L, Wang LS, Li B, Fu B, Zhang CP (2016). Operationally simple hydrotrifluoromethylation of alkenes with sodium triflinate enabled by Ir photoredox catalysis. Chem. Commun..

[16] Zheng YR, Vernieres J, Wang Z, Zhang K, Hochfilzer D (2022). Monitoring oxygen production on mass-selected iridium–tantalum oxide electrocatalysts. Nat. Energy.

[17] Hunt ST, Milina M, Alba-Rubio AC, Hendon CH, Dumesic JA (2016). Self-assembly of noble metal monolayers on transition metal carbide nanoparticle catalysts. Science.

[18] Zhao P, Zhang B, Hao X, Yi W, Chen J (2022). Rational design and synthesis of adjustable Pt and Pt-based 3D-nanoframeworks. ACS Appl. Energy Mater..

[19] Li D, Li T, Hao G, Guo W, Chen S (2020). IrO_2_ nanoparticle-decorated single-layer NiFe LDHs nanosheets with oxygen vacancies for the oxygen evolution reaction. Chem. Eng. J..

[20] Zhang H, Qiu X, Chen Y, Wang S, Skrabalak SE (2020). Shape control of monodispersed sub-5 nm Pd tetrahedrons and laciniate Pd nanourchins by maneuvering the dispersed state of additives for boosting ORR performance. Small.

[21] Song K, Feng Y, Zhang W, Zheng W (2022). MOFs fertilized transition-metallic single-atom electrocatalysts for highly-efficient oxygen reduction: spreading the synthesis strategies and advanced identification. J. Energy Chem..

[22] Fan M, Zhang B, Wang L, Li Z, Liang X (2021). Germanium-regulated adsorption site preference on ruthenium electrocatalyst for efficient hydrogen evolution. Chem. Commun..

[23] Luo W, Liu H, Liu X, Liu L, Zhao W (2021). Biocompatibility nanoprobe of MXene N-Ti_3_C_2_ quantum dot/Fe^3+^ for detection and fluorescence imaging of glutathione in living cells. Colloids Surf. B Biointerf..

[24] Luo M, Zhao Z, Zhang Y, Sun Y, Xing Y (2019). PdMo bimetallene for oxygen reduction catalysis. Nature.

[25] Wang M, Wang L, Li H, Du W, Khan MU (2015). Ratio-controlled synthesis of CuNi octahedra and nanocubes with enhanced catalytic activity. J. Am. Chem. Soc..

[26] Qian G, Chen J, Yu T, Luo L, Yin S (2021). N-doped graphene-decorated NiCo alloy coupled with mesoporous NiCoMoO nano-sheet heterojunction for enhanced water electrolysis activity at high current density. Nano-Micro Lett..

[27] Jiang H, Hu Y, Guo S, Yan C, Lee PS (2014). Rational design of MnO/carbon nanopeapods with internal void space for high-rate and long-life Li-ion batteries. ACS Nano.

[28] Zhang X, Li J, Yang Y, Zhang S, Zhu H (2018). Co_3_O_4_/Fe_0.33_Co_0.66_P interface nanowire for enhancing water oxidation catalysis at high current density. Adv. Mater..

[29] Noori MT, Tiwari BR, Ghangrekar MM, Min B (2019). Azadirachta indica leaf-extract-assisted synthesis of CoO–NiO mixed metal oxide for application in a microbial fuel cell as a cathode catalyst. Sustain. Energy Fuels.

[30] Favet T, Cottineau T, Keller V, Khakani MAE (2020). Comparative study of the photocatalytic effects of pulsed laser deposited CoO and NiO nanoparticles onto TiO_2_ nanotubes for the photoelectrochemical water splitting. Sol. Energy Mater. Sol. Cells.

[31] Liu W, Zheng D, Deng T, Chen Q, Zhu C (2021). Boosting electrocatalytic activity of 3d-block metal (hydro)oxides by ligand-induced conversion. Angew. Chem. Int. Ed..

[32] Hu L, Li M, Wei X, Wang H, Wu Y (2020). Modulating interfacial electronic structure of CoNi LDH nanosheets with Ti_3_C_2_T_x_ MXene for enhancing water oxidation catalysis. Chem. Eng. J..

[33] Feng Y, Song K, Zhang W, Zhou X, Yoo SJ (2022). Efficient ORR catalysts for zinc-air battery: biomass-derived ultra-stable Co nanoparticles wrapped with graphitic layers via optimizing electron transfer. J. Energy Chem..

[34] Bicalho HA, Lopez JL, Binatti I, Batista PFR, Ardisson JD (2017). Facile synthesis of highly dispersed Fe(II)-doped g-C_3_N_4_ and its application in Fenton-like catalysis. Mol. Catal..

[35] Chen Y, Zhou Q, Zhao G, Yu Z, Wang X (2018). Electrochemically inert g-C_3_N_4_ promotes water oxidation Catalysis. Adv. Funct. Mater..

[36] Huang C, Chen C, Zhang M, Lin L, Ye X (2015). Carbon-doped BN nanosheets for metal-free photoredox catalysis. Nat. Commun..

[37] Sinthika S, Kumar EM, Thapa R (2014). Doped h-BN monolayer as efficient noble metal-free catalysts for CO oxidation: the role of dopant and water in activity and catalytic de-poisoning. J. Mater. Chem. A.

[38] Luo Y, Zhang Z, Yang F, Li J, Liu Z (2021). Stabilized hydroxide-mediated nickel-based electrocatalysts for high-current-density hydrogen evolution in alkaline media. Energy Environ. Sci..

[39] Kwon KC, Baek JH, Hong K, Kim SY, Jang HW (2022). Memristive devices based on two-dimensional transition metal chalcogenides for neuromorphic computing. Nano-Micro Lett..

[40] Zhang C, Ma Y, Zhang X, Abdolhosseinzadeh S, Sheng H (2020). Two-dimensional transition metal carbides and nitrides (MXenes): synthesis, properties, and electrochemical energy storage applications. Energy Environ. Mater..

[41] Yin L, Li Y, Yao X, Wang Y, Jia L (2021). MXenes for solar cells. Nano-Micro Lett..

[42] Khan K, Tareen AK, Aslam M, Sagar RUR, Zhang B (2020). Recent progress, challenges, and prospects in two-dimensional photo-catalyst materials and environmental remediation. Nano-Micro Lett..

[43] Fan C, Shi J, Zhang Y, Quan W, Chen X (2022). Fast and recoverable NO_2_ detection achieved by assembling ZnO on Ti_3_C_2_T_x_ MXene nanosheets under UV illumination at room temperature. Nanoscale.

[44] Huang K, Li Z, Lin J, Han G, Huang P (2018). Two-dimensional transition metal carbides and nitrides (MXenes) for biomedical applications. Chem. Soc. Rev..

[45] Anasori B, Lukatskaya MR, Gogotsi Y (2017). 2D metal carbides and nitrides (MXenes) for energy storage. Nat. Rev. Mater..

[46] Niu Y, Tian C, Gao J, Fan F, Zhang Y (2021). Nb_2_C MXenes modified SnO_2_ as high quality electron transfer layer for efficient and stability perovskite solar cells. Nano Energy.

[47] Zhu X, Liu B, Hou H, Huang Z, Zeinu KM (2017). Alkaline intercalation of Ti_3_C_2_ MXene for simultaneous electrochemical detection of Cd(II), Pb(II), Cu(II) and Hg(II). Electrochim. Acta.

[48] Wu X, Wang Z, Yu M, Xiu L, Qiu J (2017). Stabilizing the MXenes by carbon nanoplating for developing hierarchical nanohybrids with efficient lithium storage and hydrogen evolution capability. Adv. Mater..

[49] Yu Y, Yi P, Xu W, Sun X, Deng G (2022). Environmentally tough and stretchable MXene organohydrogel with exceptionally enhanced electromagnetic interference shielding performances. Nano-Micro Lett..

[50] VahidMohammadi A, Rosen J, Gogotsi Y (2021). The world of two-dimensional carbides and nitrides (MXenes). Science.

[51] Bu F, Zagho MM, Ibrahim Y, Ma B, Elzatahry A (2020). Porous MXenes: synthesis, structures, and applications. Nano Today.

[52] Li Y, Shao H, Lin Z, Lu J, Liu L (2021). Author correction: a general Lewis acidic etching route for preparing MXenes with enhanced electrochemical performance in non-aqueous electrolyte. Nat. Mater..

[53] Lukatskaya MR, Halim J, Dyatkin B, Naguib M, Buranova YS (2014). Room-temperature carbide-derived carbon synthesis by electrochemical etching of MAX phases. Angew. Chem. Int. Ed..

[54] Pang SY, Wong YT, Yuan S, Liu Y, Tsang MK (2019). Universal strategy for HF-free facile and rapid synthesis of two-dimensional MXenes as multifunctional energy materials. J. Am. Chem. Soc..

[55] Xu Q, Niu Y, Li J, Yang Z, Gao J (2022). Recent progress of quantum dots for energy storage applications. Carbon Neutrality.

[56] Wang Z, Xuan J, Zhao Z, Li Q, Geng F (2017). Versatile cutting method for producing fluorescent ultrasmall MXene sheets. ACS Nano.

[57] Zhang YZ, El-Demellawi JK, Jiang Q, Ge G, Liang H (2020). MXene hydrogels: fundamentals and applications. Chem. Soc. Rev..

[58] Xue Q, Zhang H, Zhu M, Pei Z, Li H (2017). Photoluminescent Ti_3_C_2_ MXene quantum dots for multicolor cellular imaging. Adv. Mater..

[59] Lu S, Sui L, Liu Y, Yong X, Xiao G (2019). White photoluminescent Ti_3_C_2_ MXene quantum dots with two-photon fluorescence. Adv. Sci..

[60] Gao G, O’Mullane AP, Du A (2017). 2D MXenes: a new family of promising catalysts for the hydrogen evolution reaction. ACS Catal..

[61] Seh ZW, Fredrickson KD, Anasori B, Kibsgaard J, Strickler AL (2016). Two-dimensional molybdenum carbide (MXene) as an efficient electrocatalyst for hydrogen evolution. ACS Energy Lett..

[62] Li S, Tuo P, Xie J, Zhang X, Xu J (2018). Ultrathin MXene nanosheets with rich fluorine termination groups realizing efficient electrocatalytic hydrogen evolution. Nano Energy.

[63] Diao J, Hu M, Lian Z, Li Z, Zhang H (2018). Ti_3_C_2_T_x_ MXene catalyzed ethylbenzene dehydrogenation: active sites and mechanism exploration from both experimental and theoretical aspects. ACS Catal..

[64] Chen HR, Meng WM, Wang RY, Chen FL, Li T (2022). Engineering highly graphitic carbon quantum dots by catalytic dehydrogenation and carbonization of Ti_3_C_2_T_x_-MXene wrapped polystyrene spheres. Carbon.

[65] Li J, Wang S, Du Y, Liao W (2019). Catalytic effect of Ti_2_C MXene on the dehydrogenation of MgH_2_. Int. J. Hydro. Energy.

[66] Yang X, Jia Q, Duan F, Hu B, Wang M (2019). Multiwall carbon nanotubes loaded with MoS_2_ quantum dots and MXene quantum dots: non-Pt bifunctional catalyst for the methanol oxidation and oxygen reduction reactions in alkaline solution. Appl. Surf. Sci..

[67] Zeng Z, Yan Y, Chen J, Zan P, Tian Q (2019). Boosting the photocatalytic ability of Cu_2_O nanowires for CO_2_ conversion by mxene quantum dots. Adv. Funct. Mater..

[68] Du X, Zhao T, Xiu Z, Xing Z, Li Z (2020). BiVO_4_@ZnIn_2_S_4_/Ti_3_C_2_ MXene quantum dots assembly all-solid-state direct Z-Scheme photocatalysts for efficient visible-light-driven overall water splitting. Appl. Mater. Today.

[69] Qin J, Liu B, Lam KH, Song S, Li X (2020). 0D/2D MXene quantum dot/Ni-MOF ultrathin nanosheets for enhanced N_2_ photoreduction. ACS Sustain. Chem. Eng..

[70] Jin Z, Liu C, Liu Z, Han J, Fang Y (2020). Rational design of hydroxyl-rich Ti_3_C_2_T_x_ MXene quantum dots for high-performance electrochemical N_2_ reduction. Adv. Energy Mater..

[71] Wang H, Zhao R, Hu H, Fan X, Zhang D (2020). 0D/2D Heterojunctions of Ti_3_C_2_ MXene QDs/SiC as an efficient and robust photocatalyst for boosting the visible photocatalytic NO pollutant removal ability. ACS Appl. Mater. Interfaces.

[72] Tang R, Zhou S, Li C, Chen R, Zhang L (2020). Janus-structured Co-Ti_3_C_2_ MXene quantum dots as a Schottky catalyst for high-performance photoelectrochemical water oxidation. Adv. Funct. Mater..

[73] Lin S, Zhang N, Wang F, Lei J, Zhou L (2021). Carbon vacancy mediated incorporation of Ti_3_C_2_ quantum dots in a 3D inverse opal g-C_3_N_4_ Schottky junction catalyst for photocatalytic H_2_O_2_ production. ACS Sustain. Chem. Eng..

[74] Yuan Z, Huang H, Li N, Chen D, Xu Q (2021). All-solid-state WO_3_/TQDs/In_2_S_3_ Z-scheme heterojunctions bridged by Ti_3_C_2_ quantum dots for efficient removal of hexavalent chromium and bisphenol A. J. Hazard. Mater..

[75] Lai C, An Z, Yi H, Huo X, Qin L (2021). Enhanced visible-light-driven photocatalytic activity of bismuth oxide via the decoration of titanium carbide quantum dots. J. Colloid Interface Sci..

[76] Chang B, Guo Y, Liu H, Li L, Yang B (2022). Engineering a surface defect-rich Ti_3_C_2_ quantum dots/mesoporous C_3_N_4_ hollow nanosphere Schottky junction for efficient N_2_ photofixation. J. Mater. Chem. A.

[77] Guo Y, Cheng Y, Li X, Li Q, Li X (2022). MXene quantum dots decorated Ni nanoflowers for efficient Cr (VI) reduction. J. Hazard. Mater..

[78] Yang L, Chen Z, Wang X, Jin M (2022). High-stability Ti_3_C_2_-QDs/ZnIn_2_S_4_/Ti(IV) flower-like heterojunction for boosted photocatalytic hydrogen evolution. Nanomaterials.

[79] Cheng Y, Li X, Shen P, Guo Y, Chu K (2022). MXene quantum dots/copper nanocomposites for synergistically enhanced N_2_ electroreduction. Energy Environ. Mater..

[80] Shen S, Wang J, Wu Z, Du Z, Tang Z (2020). Graphene quantum dots with high yield and high quality synthesized from low cost precursor of aphanitic graphite. Nanomaterials.

[81] Xu ZL, Lin S, Onofrio N, Zhou L, Shi F (2018). Exceptional catalytic effects of black phosphorus quantum dots in shuttling-free lithium sulfur batteries. Nat. Commun..

[82] Yu B, Chen D, Wang Z, Qi F, Zhang X (2020). Mo_2_C quantum dots@graphene functionalized separator toward high-current-density lithium metal anodes for ultrastable Li-S batteries. Chem. Eng. J..

[83] Xiao SJ, Wang LZ, Yuan MY, Huang XH, Ding JH (2020). Peroxidase-mimetic and Fenton-like activities of molybdenum oxide quantum dots. ChemistrySelect.

[84] Chen X, Sun X, Xu W, Pan G, Zhou D (2018). Ratiometric photoluminescence sensing based on Ti_3_C_2_ MXene quantum dots as an intracellular pH sensor. Nanoscale.

[85] Cao Y, Wu T, Zhang K, Meng X, Dai W (2019). Engineered exosome-mediated near-infrared-II region V_2_C quantum dot delivery for nucleus-target low-temperature photothermal therapy. ACS Nano.

[86] Xu Q, Ma J, Khan W, Zeng X, Li N (2020). Highly green fluorescent Nb_2_C MXene quantum dots. Chem. Commun..

[87] Shao J, Zhang J, Jiang C, Lin J, Huang P (2020). Biodegradable titanium nitride MXene quantum dots for cancer phototheranostics in NIR-I/II biowindows. Chem. Eng. J..

[88] Jin Z, Xu G, Niu Y, Ding X, Han Y (2020). Ti_3_C_2_T_x_ MXene-derived TiO_2_/C-QDs as oxidase mimics for the efficient diagnosis of glutathione in human serum. J. Mater. Chem. B.

[89] Kong W, Niu Y, Liu M, Zhang K, Xu G (2019). One-step hydrothermal synthesis of fluorescent MXene-like titanium chock for carbonitride quantum dots. Inorg. Chem. Commun..

[90] Zhang H, Yang L, Zhang P, Lu C, Sha D (2021). MXene-derived Ti_n_O_2n−1_ quantum dots distributed on porous carbon nanosheets for stable and long-life Li–S batteries: enhanced polysulfide mediation via defect engineering. Adv. Mater..

[91] Fan Y, Li L, Zhang Y, Zhang X, Geng D (2022). Recent advances in growth of transition metal carbides and nitrides (MXenes) crystals. Adv. Funct. Mater..

[92] Manawi YM, Ihsanullah A, Samara T, Al-Ansari MAA (2018). A review of carbon nanomaterials’ synthesis via the chemical vapor deposition (CVD) method. Materials.

[93] Maleski K, Mochalin VN, Gogotsi Y (2017). Dispersions of two-dimensional titanium carbide mxene in organic solvents. Chem. Mater..

[94] Mashtalir O, Cook KM, Mochalin VN, Crowe M, Barsoum MW (2014). Dye adsorption and decomposition on two-dimensional titanium carbide in aqueous media. J. Mater. Chem. A.

[95] Rolsten RF (1968). Solution chemistry of the electrochemical machining of titanium, niobium and tantalum. J. Appl. Chem..

[96] Xu G, Niu Y, Yang X, Jin Z, Wang Y (2018). Preparation of Ti_3_C_2_T_x_ MXene-derived quantum dots with white/blue-emitting photoluminescence and electrochemiluminescence Adv. Opt. Mater..

[97] Naguib M, Mochalin VN, Barsoum MW, Gogotsi Y (2014). 25th anniversary article: MXenes: a new family of two-dimensional materials. Adv. Mater..

[98] Hong L, Klie RF, Öğüt S (2016). First-principles study of size- and edge-dependent properties of MXene nanoribbons. Phys. Rev. B.

[99] Lian P, Dong Y, Wu ZS, Zheng S, Wang X (2017). Alkalized Ti_3_C_2_ MXene nanoribbons with expanded interlayer spacing for high-capacity sodium and potassium ion batteries. Nano Energy.

[100] Mashtalir O, Naguib M, Mochalin VN, Dall'Agnese Y, Heon M (2013). Intercalation and delamination of layered carbides and carbonitrides. Nat. Commun..

[101] Zhou H, Chen Z, López AV, López ED, Lam E (2021). Engineering the Cu/Mo_2_CT_x_ (MXene) interface to drive CO_2_ hydrogenation to methanol. Nat. Catal..

[102] Lukatskaya MR, Bak SM, Yu X, Yang XQ, Barsoum MW (2015). Probing the mechanism of high capacitance in 2D titanium carbide using in situ X-ray absorption spectroscopy. Adv. Energy Mater..

[103] Handoko AD, Fredrickson KD, Anasori B, Convey KW, Johnson LR (2018). Tuning the basal plane functionalization of two-dimensional metal carbides (MXenes) to control hydrogen evolution activity. ACS Appl. Energy Mater..

[104] Yang S, Zhang P, Wang F, Ricciardulli AG, Lohe MR (2018). Fluoride-free synthesis of two-dimensional titanium carbide (MXene) using a binary aqueous system. Angew. Chem. Int. Ed..

[105] Jiang Y, Sun T, Xie X, Jiang W, Li J (2019). Oxygen-functionalized ultrathin Ti_3_C_2_T_x_ MXene for enhanced electrocatalytic hydrogen evolution. Chemsuschem.

[106] Pandey M, Thygesen KS (2017). Two-dimensional MXenes as catalysts for electrochemical hydrogen evolution: a computational screening study. J. Phys. Chem. C.

[107] Wang L, Zhang N, Li Y, Kong W, Gou J (2021). Mechanism of nitrogen-doped Ti_3_C_2_ quantum dots for free-radical scavenging and the ultrasensitive H_2_O_2_ detection performance. ACS Appl. Mater. Interfaces.

[108] Xu N, Li H, Gan Y, Chen H, Li W (2020). Zero-dimensional MXene-based optical devices for ultrafast and ultranarrow photonics applications. Adv. Sci..

[109] Yang F, Ge Y, Yin T, Guo J, Zhang F (2020). Ti_3_C_2_T_x_ MXene quantum dots with enhanced stability for ultrafast photonics. ACS Appl. Nano Mater..

[110] Yu X, Cai X, Cui H, Lee SW, Yu XF (2017). Fluorine-free preparation of titanium carbide MXene quantum dots with high near-infrared photothermal performances for cancer therapy. Nanoscale.

[111] Neupane GP, Wang B, Tebyetekerwa M, Nguyen HT, Taheri M (2021). Highly enhanced light-matter interaction in MXene quantum dots-monolayer WS_2_ heterostructure. Small.

[112] Wang X, Zhang X, Cao H, Huang Y (2020). A facile and rapid approach to synthesize uric acid-capped Ti_3_C_2_ MXene quantum dots for the sensitive determination of 2,4,6-trinitrophenol both on surfaces and in solution. J. Mater. Chem. B.

[113] Liu M, Bai Y, He Y, Zhou J, Ge Y (2021). Facile microwave-assisted synthesis of Ti_3_C_2_ MXene quantum dots for ratiometric fluorescence detection of hypochlorite. Microchim. Acta.

[114] Zhang T, Jiang X, Li G, Yao Q, Lee JY (2018). A red-phosphorous-assisted ball-milling synthesis of few-layered Ti_3_C_2_T_x_ (MXene) nanodot composite. ChemNanoMat.

[115] Yuan Y, Jiang L, Li X, Zuo P, Zhang X (2022). Ultrafast shaped laser induced synthesis of MXene quantum dots/graphene for transparent supercapacitors. Adv. Mater..

[116] Alijani H, Rezk AR, Farsani MMK, Ahmed H, Halim J (2021). Acoustomicrofluidic synthesis of pristine ultrathin Ti_3_C_2_T_z_ MXene nanosheets and quantum dots. ACS Nano.

[117] Zhao L, Wang Z, Li Y, Wang S, Wang L (2021). Designed synthesis of chlorine and nitrogen co-doped Ti_3_C_2_ MXene quantum dots and their outstanding hydroxyl radical scavenging properties. J. Mater. Sci. Tech..

[118] Shao B, Liu Z, Zeng G, Wang H, Liang Q (2020). Two-dimensional transition metal carbide and nitride (MXene) derived quantum dots (QDs): synthesis, properties, applications and prospects. J. Mater. Chem. A.

[119] Wang Y, Li C, Han X, Liu D, Zhao H (2018). Ultrasmall Mo_2_C nanoparticle-decorated carbon polyhedrons for enhanced microwave absorption. ACS Appl. Nano Mater..

[120] Cheng H, Ding LX, Chen GF, Zhang L, Xue J (2018). Molybdenum carbide nanodots enable efficient electrocatalytic nitrogen fixation under ambient conditions. Adv. Mater..

[121] Urbankowski P, Anasori B, Hantanasirisakul K, Yang L, Zhang L (2017). 2D molybdenum and vanadium nitrides synthesized by ammoniation of 2D transition metal carbides (MXenes). Nanoscale.

[122] Du CF, Sun X, Yu H, Liang Q, Dinh KN (2019). Synergy of Nb doping and surface alloy enhanced on water-alkali electrocatalytic hydrogen generation performance in Ti-based MXene. Adv. Sci..

[123] Li J, Yan D, Hou S, Li Y, Lu T (2018). Improved sodium-ion storage performance of Ti_3_C_2_T_x_ MXenes by sulfur doping. J. Mater. Chem. A.

[124] Ramalingam V, Varadhan P, Fu HC, Kim H, Zhang D (2019). Heteroatom-mediated interactions between ruthenium single atoms and an MXene support for efficient hydrogen evolution. Adv. Mater..

[125] Lu C, Yang L, Yan B, Sun L, Zhang P (2020). Nitrogen-doped Ti_3_C_2_ MXene: mechanism investigation and electrochemical analysis. Adv. Funct. Mater..

[126] Ling Z, Ren CE, Zhao MQ, Yang J, Giammarco JM (2014). Flexible and conductive MXene films and nanocomposites with high capacitance. PNAS.

[127] Sun R, Zhang HB, Liu J, Xie X, Yang R (2017). Highly conductive transition metal carbide/carbonitride(MXene)@polystyrene nanocomposites fabricated by electrostatic assembly for highly efficient electromagnetic interference shielding. Adv. Funct. Mater..

[128] Guo J, Zhao Y, Liu A, Ma T (2019). Electrostatic self-assembly of 2D delaminated MXene (Ti_3_C_2_) onto Ni foam with superior electrochemical performance for supercapacitor. Electrochim. Acta.

[129] Xu Q, Gao J, Wang S, Wang Y, Liu D (2021). Quantum dots in cell imaging and their safety issues. J. Mater. Chem. B.

[130] Yan X, Ma J, Yu K, Li J, Yang L (2020). Highly green fluorescent Nb_2_C MXene quantum dots for Cu^2+^ ion sensing and cell imaging. Chin. Chem. Lett..

[131] Qiao W, Feng Z (2021). 100 MHz frequency-spacing switchable single-dual-frequency laser based on MXene QDs and a phase-shifted FBG. Opt. Exp..

[132] Liu X, Ji L, Zhu F, Gan Y, Wen Q (2021). Linear-cavity-based single frequency fiber laser with a loop mirror and Ti_2_CT_x_ quantum dots. Opt. Mater..

[133] Xu Q, Yang W, Wen Y, Liu S, Liu Z (2019). Hydrochromic full-color MXene quantum dots through hydrogen bonding toward ultrahigh-efficiency white light-emitting diodes. Appl. Mater. Today.

[134] Chen X, Xu W, Ding N, Ji Y, Pan G (2020). Dual interfacial modification engineering with 2D MXene quantum dots and copper sulphide nanocrystals enabled high-performance perovskite solar cells. Adv. Funct. Mater..

[135] Ge J, Li W, He X, Chen H, Fang W (2020). Charge behavior modulation by titanium-carbide quantum dots and nanosheets for efficient perovskite solar cells. Mater. Today Energy.

[136] Han X, Li N, Xiong P, Jung MG, Kang Y (2021). Electronically coupled layered double hydroxide/MXene quantum dot metallic hybrids for high-performance flexible zinc–air batteries. InfoMat.

[137] Wang P, Zhao D, Hui X, Qian Z, Zhang P (2021). Bifunctional catalytic activity guided by rich crystal defects in Ti_3_C_2_ MXene quantum dot clusters for Li–O_2_ batteries. Adv. Energy Mater..

[138] Yang Y, Lu H, Feng S, Yang L, Dong H (2021). Modulation of perovskite crystallization processes towards highly efficient and stable perovskite solar cells with MXene quantum dot-modified SnO_2_. Energy Environ. Sci..

[139] Gao X, Shao X, Qin L, Li Y, Huang S (2021). N, N-dimethyl formamide regulating fluorescence of MXene quantum dots for the sensitive determination of F^e3^+. Nanoscale Res. Lett..

[140] Ai F, Fu C, Cheng G, Zhang H, Feng Y (2021). Amino-functionalized Ti_3_C_2_ MXene quantum dots as photoluminescent sensors for diagnosing histidine in human serum. ACS Appl. Nano Mater..

[141] Jiang X, Wang H, Shen Y, Hu N, Shi W (2022). Nitrogen-doped Ti_3_C_2_ MXene quantum dots as novel high-efficiency electrochemiluminescent emitters for sensitive mucin 1 detection. Sens. Actuators B Chem..

[142] Bai Y, He Y, Wang Y, Song G (2021). Nitrogen, boron-doped Ti_3_C_2_ MXene quantum dot-based ratiometric fluorescence sensing platform for point-of-care testing of tetracycline using an enhanced antenna effect by Eu^3+^. Microchim. Acta.

[143] Zhao Z, Wu X, Luo C, Wang Y, Chen W (2022). Rational design of Ti_3_C_2_Cl_2_ MXenes nanodots-interspersed MXene@NiAl-layered double hydroxides for enhanced pseudocapacitor storage. J. Colloid Interface Sci..

[144] Guan Q, Ma J, Yang W, Zhang R, Zhang X (2019). Highly fluorescent Ti_3_C_2_ MXene quantum dots for macrophage labeling and Cu^2+^ ion sensing. Nanoscale.

[145] Gou J, Zhao L, Li Y, Zhang J (2021). Nitrogen-doped Ti_2_C MXene quantum dots as antioxidants. ACS Appl. Nano Mater..

[146] Fu C, Ai F, Huang J, Shi Z, Yan X (2022). Eu doped Ti_3_C_2_ quantum dots to form a ratiometric fluorescence platform for visual and quantitative point-of-care testing of tetracycline derivatives. Spectrochim. Acta Part A Mol Biomol. Spectrosco..

[147] Xu Q, Ding L, Wen Y, Yang W, Zhou H (2018). High photoluminescence quantum yield of 18.7% by using nitrogen-doped Ti3C2 MXene quantum dots. J. Mater. Chem. C.

[148] Yan F, Sun J, Zang Y, Sun Z, Zhang H (2021). Solvothermal synthesis of nitrogen-doped MXene quantum dots for the detection of alizarin red based on inner filter effect. Dyes Pigments.

[149] Feng Y, Zhou F, Deng Q, Peng C (2020). Solvothermal synthesis of in situ nitrogen-doped Ti_3_C_2_ MXene fluorescent quantum dots for selective Cu^2+^ detection. Ceram. Int..

[150] Huang D, Wu Y, Ai F, Zhou X, Zhu G (2021). Fluorescent nitrogen-doped Ti_3_C_2_ MXene quantum dots as a unique “on-off-on” nanoprobe for chrominum (VI) and ascorbic acid based on inner filter effect. Sens. Actuators B Chem..

[151] Liu M, Zhou J, He Y, Cai Z, Ge Y (2019). ε-Poly-L-lysine-protected Ti_3_C_2_ MXene quantum dots with high quantum yield for fluorometric determination of cytochrome c and trypsin. Microchim. Acta.

[152] Liu M, He Y, Zhou J, Ge Y, Zhou J (2020). A ’’naked-eye’’ colorimetric and ratiometric fluorescence probe for uric acid based on Ti_3_C_2_ MXene quantum dots. Anal. Chim. Acta.

[153] Lu Q, Wang J, Li B, Weng C, Li X (2020). Dual-emission reverse change ratio photoluminescence sensor based on a probe of nitrogen-doped Ti_3_C_2_ quantum dots@DAP to detect H_2_O_2_ and xanthine. Anal. Chem..

[154] Al-Duais MA, Mohammedsaleh ZM, Al-Shehri HS, Al-Awthan YS, Bani-Atta SA (2022). Bovine serum albumin functionalized blue emitting Ti_3_C_2_ MXene quantum dots as a sensitive fluorescence probe for Fe^3+^ ion detection and its toxicity analysis. Luminescence.

[155] Mao H, Gu C, Yan S, Xin Q, Cheng S (2020). MXene quantum dot/polymer hybrid structures with tunable electrical conductance and resistive switching for nonvolatile memory devices. Adv. Electron. Mater..

[156] Pandey P, Sengupta A, Parmar S, Bansode U, Gosavi S (2020). CsPbBr_3_-T_i3_C_2_T_x_ MXene QD/QD heterojunction: photoluminescence quenching, charge transfer, and Cd ion sensing application. ACS Appl. Nano Mater..

[157] Chen X, Xu W, Shi Z, Ji Y, Lyu J (2021). Plasmonic gold nanorods decorated Ti_3_C_2_ MXene quantum dots-interspersed nanosheet for full-spectrum photoelectrochemical water splitting. Chem. Eng. J..

[158] Nie Y, Liang Z, Wang P, Ma Q, Su X (2021). MXene-derived quantum dot@gold nanobones heterostructure-based electrochemiluminescence sensor for triple-negative breast cancer diagnosis. Anal. Chem..

[159] He F, Zhu B, Cheng B, Yu J, Ho W (2020). 2D/2D/0D TiO_2_/C_3_N_4_/Ti_3_C_2_ MXene composite S-scheme photocatalyst with enhanced CO_2_ reduction activity. Appl. Catal. B Environ..

[160] Li Y, Ding L, Guo Y, Liang Z, Cui H (2019). Boosting the photocatalytic ability of g-C_3_N_4_ for hydrogen production by Ti_3_C_2_ MXene quantum dots. ACS Appl. Mater. Interfaces.

[161] Song Y, Zhang X, Zhang Y, Zhai P, Li Z (2022). Engineering MoO_x_/MXene hole transfer layers for unexpected boosting photoelectrochemical water oxidation. Angew. Chem. Int. Ed..

[162] Song L, Zhu S, Tong L, Wang W, Ouyang C (2021). MXene quantum dot rivet reinforced Ni-Co LDH for boosting electrochemical activity and cycling stability. Mater. Adv..

[163] Chen X, Li J, Pan G, Xu W, Zhu J (2019). Ti_3_C_2_ MXene quantum dots/TiO_2_ inverse opal heterojunction electrode platform for superior photoelectrochemical biosensing. Sens. Actuators B Chem..

[164] Liu Y, Zhang X, Zhang W, Ge X, Wang Y (2022). MXene-based quantum dots optimize hydrogen production via spontaneous evolution of Cl- to O-terminated surface groups. Energy Environ. Mater..

[165] Sun J, Du H, Chen Z, Wang L, Shen G (2021). MXene quantum dot within natural 3D watermelon peel matrix for biocompatible flexible sensing platform. Nano Res..

[166] Clarke SJ, Hollmann CA, Zhang Z, Suffern D, Bradforth SE (2006). Photophysics of dopamine-modified quantum dots and effects on biological systems. Nat. Mater..

[167] Zhu MQ, Chang E, Sun J, Drezek RA (2007). Surface modification and functionalization of semiconductor quantum dots through reactive coating of silanes in toluene. J. Mater. Chem..

[168] Yue Z, Lisdat F, Parak WJ, Hickey SG, Tu L (2013). Quantum-dot-based photoelectrochemical sensors for chemical and biological detection. ACS Appl. Mater. Interfaces.

[169] Yu B, Tawiah B, Wang LQ, Yuen ACY, Zhang ZC (2019). Interface decoration of exfoliated MXene ultra-thin nanosheets for fire and smoke suppressions of thermoplastic polyurethane elastomer. J. Hazard. Mater..

[170] Zhu SE, Wang FD, Liu JJ, Wang LL, Wang C (2021). BODIPY coated on MXene nanosheets for improving mechanical and fire safety properties of ABS resin. Compos. Part B Eng..

[171] Li J, Guan Q, Wu H, Liu W, Lin Y (2019). Highly active and stable metal single-atom catalysts achieved by strong electronic metal–support interactions. J. Am. Chem. Soc..

[172] Huang ZF, Song J, Du Y, Dou S, Sun L (2019). Optimizing interfacial electronic coupling with metal oxide to activate inert polyaniline for superior electrocatalytic hydrogen generation. Carbon Energy.

[173] Wu Y, Cai J, Xie Y, Niu S, Zang Y (2020). Regulating the interfacial electronic coupling of Fe_2_N via orbital steering for hydrogen evolution catalysis. Adv. Mater..

[174] Su DW, Ran J, Zhuang ZW, Chen C, Qiao SZ (2020). Atomically dispersed Ni in cadmium-zinc sulfide quantum dots for high-performance visible-light photocatalytic hydrogen production. Sci. Adv..

[175] Wang Q, Li J, Tu X, Liu H, Shu M (2020). Single atomically anchored cobalt on carbon quantum dots as efficient photocatalysts for visible light-promoted oxidation reactions. Chem. Mater..

[176] Jin S, Ni Y, Hao Z, Zhang K, Lu Y (2020). A universal graphene quantum dot tethering design strategy to synthesize single-atom catalysts. Angew. Chem. Int. Ed..

[177] Xia C, Qiu Y, Xia Y, Zhu P, King G (2021). General synthesis of single-atom catalysts with high metal loading using graphene quantum dots. Nat. Chem..

[178] Cai Y, Fu J, Zhou Y, Chang YC, Min Q (2021). Insights on forming N, O-coordinated Cu single-atom catalysts for electrochemical reduction C_O_2 to methane. Nat. Commun..

[179] Sivanantham A, Ganesan P, Shanmugam S (2016). Hierarchical NiCo_2_S_4_ nanowire arrays supported on Ni foam: an efficient and durable bifunctional electrocatalyst for oxygen and hydrogen evolution reactions. Adv. Funct. Mater..

[180] Di J, Xiong J, Li H, Liu Z (2018). Ultrathin 2D photocatalysts: electronic-structure tailoring, hybridization, and applications. Adv. Mater..

[181] Jiang H, Wang Z, Yang Q, Tan L, Dong L (2019). Ultrathin Ti_3_C_2_T_x_ (MXene) nanosheet-wrapped NiSe_2_ octahedral crystal for enhanced supercapacitor performance and synergetic electrocatalytic water splitting. Nano-Micro Lett..

[182] Du Z, Yang S, Li S, Lou J, Zhang S (2020). Conversion of non-van der Waals solids to 2D transition-metal chalcogenides. Nature.

[183] Xu K, Cao P, Heath JR (2010). Graphene visualizes the first water adlayers on mica at ambient conditions. Science.

[184] Kim H, Kim HH, Jang JI, Lee SK, Lee GW (2014). Doping graphene with an atomically thin two dimensional molecular layer. Adv. Mater..

[185] Sang X, Xie Y, Lin MW, Alhabeb M, Aken KLV (2016). Atomic defects in monolayer titanium carbide (Ti_3_C_2_T_x_) MXene. ACS Nano.

[186] Lipatov A, Alhabeb M, Lukatskaya MR, Boson A, Gogotsi Y (2016). Effect of synthesis on quality, electronic properties and environmental stability of individual monolayer Ti_3_C_2_ MXene flakes. Adv. Electron. Mater..

[187] Hong J, Jin C, Yuan J, Zhang Z (2017). Atomic defects in two-dimensional materials: from single-atom spectroscopy to functionalities in opto-/electronics, nanomagnetism, and catalysis. Adv. Mater..

[188] Zhao MQ, Xie X, Ren CE, Makaryan T, Anasori B (2017). Hollow MXene spheres and 3D macroporous MXene frameworks for Na-ion storage. Adv. Mater..

[189] Shang T, Lin Z, Qi C, Liu X, Li P (2019). 3D macroscopic architectures from self-assembled MXene hydrogels. Adv. Funct. Mater..

[190] Li X, Yin X, Liang S, Li M, Cheng L (2019). 2D carbide MXene Ti_2_CT_X_ as a novel high-performance electromagnetic interference shielding material. Carbon.

[191] Han M, Yin X, Wu H, Hou Z, Song C (2016). Ti_3_C_2_ MXenes with modified surface for high-performance electromagnetic absorption and Shielding in the X-band. ACS Appl. Mater. Interfaces.

[192] Sun W, Shah SA, Chen Y, Tan Z, Gao H (2017). Electrochemical etching of Ti_2_AlC to Ti_2_CT_x_ (MXene) in low-concentration hydrochloric acid solution. J. Mater. Chem. A.

[193] Zhang CJ, Pinilla S, McEvoy N, Cullen CP, Anasori B (2017). Oxidation stability of colloidal two-dimensional titanium carbides (MXenes). Chem. Mater..

[194] Zhao X, Vashisth A, Prehn E, Sun W, Shah SA (2019). Antioxidants unlock shelf-stable Ti_3_C_2_T_x_ (MXene) nanosheet dispersions. Matter.

[195] Yang EJ, Jeon OS, Yang JU, Shin MK, Yoo YJ (2020). Room temperature manufacturing photoluminescent graphene quantum dots based on MXene. Carbon.

[196] Shi X, Meng H, Sun Y, Qu L, Lin Y (2019). Far-red to near-infrared carbon dots: preparation and applications in biotechnology. Small.

[197] Liu Z, Li B, Feng Y, Jia D, Li C (2021). Strong electron coupling of Ru and vacancy-rich carbon dots for synergistically enhanced hydrogen evolution reaction. Small.

[198] Wareing TC, Gentile P, Phan AN (2021). Biomass-based carbon dots: current development and future perspectives. ACS Nano.

[199] Liu Y, Huang S, Li J, Wang M, Wang C (2021). 0D/2D heteronanostructure–integrated bimetallic CoCu-ZIF nanosheets and MXene-derived carbon dots for impedimetric cytosensing of melanoma B16–F10 cells. Microchim. Acta.

[200] Lee NE, Lee SY, Lim HS, Yoo SH, Cho SO (2020). A novel route to high-quality graphene quantum dots by hydrogen-assisted pyrolysis of silicon carbide. Nanomaterials.

[201] Zhou L, Wu F, Yu J, Deng Q, Zhang F (2017). Titanium carbide (Ti_3_C_2_T_x_) MXene: a novel precursor to amphiphilic carbide-derived graphene quantum dots for fluorescent ink, light-emitting composite and bioimaging. Carbon.

[202] Rafieerad A, Yan W, Amiri A, Dhingra S (2020). Bioactive and trackable MXene quantum dots for subcellular nanomedicine applications. Mater. Des..

[203] Guo Z, Zhu X, Wang S, Lei C, Huang Y (2018). Fluorescent Ti_3_C_2_ MXene quantum dots for an alkaline phosphatase assay and embryonic stem cell identification based on the inner filter effect. Nanoscale.

[204] Cai G, Yu Z, Tong P, Tang D (2019). Ti_3_C_2_ MXene quantum dot-encapsulated liposomes for photothermal immunoassays using a portable near-infrared imaging camera on a smartphone. Nanoscale.

[205] Yang G, Zhao J, Yi S, Wan X, Tang J (2020). Biodegradable and photostable Nb_2_C MXene quantum dots as promising nanofluorophores for metal ions sensing and fluorescence imaging. Sens. Actuators B Chem..

[206] Su DS, Zhang B, Schlögl R (2015). Electron microscopy of solid catalysts—transforming from a challenge to a toolbox. Chem. Rev..

[207] Zhao CX, Li BQ, Liu JN, Zhang Q (2021). Intrinsic electrocatalytic activity regulation of M-N–C single-atom catalysts for the oxygen reduction reaction. Angew. Chem. Int. Ed..

[208] Qi K, Cui X, Gu L, Yu S, Fan X (2019). Single-atom cobalt array bound to distorted 1T MoS_2_ with ensemble effect for hydrogen evolution catalysis. Nat. Commun..

[209] Zhang W, Zheng W (2016). Single atom excels as the smallest functional material. Adv. Funct. Mater..

[210] Hu L, Wu H (2013). Annealing-induced bimodal size distribution of small CdSe quantum dots with white-light emission. Phys. Status Solidi A.

[211] Duan L, Hu L, Guan X, Lin CH, Chu D (2021). Quantum dots for photovoltaics: a tale of two materials. Adv. Energy Mater..

[212] Halim J, Cook KM, Naguib M, Eklund P, Gogotsi Y (2016). X-ray photoelectron spectroscopy of select multi-layered transition metal carbides (MXenes). Appl. Surf. Sci..

[213] Li T, Yao L, Liu Q, Gu J, Luo R (2018). Fluorine-free synthesis of high-purity Ti_3_C_2_T_x_ (T=OH, O) via alkali treatment. Angew. Chem. Int. Ed..

[214] Chen L, Huang Y, Zou R, Ma J, Yang Y (2021). Regulating TiO_2_/MXenes catalysts to promote photocatalytic performance of highly selective oxidation of d-xylose. Green Chem..

[215] Qin Y, Wang Z, Liu N, Sun Y, Han D (2018). High-yield fabrication of Ti_3_C_2_T_x_ MXene quantum dots and their electrochemiluminescence behavior. Nanoscale.

[216] Fang Y, Liu Z, Han J, Jin Z, Han Y (2019). High-performance electrocatalytic conversion of N_2_ to NH_3_ using oxygen-vacancy-rich TiO_2_ in situ grown on Ti_3_C_2_T_x_ MXene. Adv. Energy Mater..

[217] Wang Y, Jia G, Cui X, Zhao X, Zhang Q (2021). Coordination number regulation of molybdenum single-atom nanozyme peroxidase-like specificity. Chem.

[218] Deng T, Zhang W, Arcelus O, Kim JG, Carrasco J (2017). Atomic-level energy storage mechanism of cobalt hydroxide electrode for pseudocapacitors. Nat. Commun..

[219] Chen W, Pei J, He CT, Wan J, Ren H (2018). Single tungsten atoms supported on MOF-derived N-doped carbon for robust electrochemical hydrogen evolution. Adv. Mater..

[220] Nordgren J, Guo J (2000). Instrumentation for soft X-ray emission spectroscopy. J. Electron. Spectrosc. Relat. Phenom..

[221] Qin T, Zhang W, Ma Y, Zhang W, Dong T (2022). Mechanistic insights into the electrochemical Li/Na/K-ion storage for aqueous bismuth anode. Energy Storage Mater..

[222] Terauchi M, Sato Y, Hyodo H, Kimura K (2009). Soft X-ray emission spectroscopy study of the valence electron states of α-rhombohedral boron. J. Phys. Conf. Series.

[223] Sharbirin AS, Akhtar S, Kim J (2021). Light-emitting MXene quantum dots. Opto-Electron. Adv..

[224] Mohanty B, Giri L, Jena BK (2021). MXene-derived quantum dots for energy conversion and storage applications. Energy Fuels.

[225] Xu X, Zhang H, Diao Q, Zhu Y, Yang G (2020). Highly sensitive fluorescent sensing for intracellular glutathione based on Ti_3_C_2_ quantum dots. J. Mater. Sci. Mater. Electron..

[226] Yang Y, Yao M, Wang X, Huang H (2019). Theoretical prediction of catalytic activity of Ti_2_C MXene as cathode for Li–O_2_ batteries. J. Phys. Chem. C.

[227] Wei B, Fu Z, Legut D, Germann TC, Du S (2021). Rational design of highly stable and active MXene-based bifunctional ORR/OER double-atom catalysts. Adv. Mater..

[228] Liu A, Liang X, Zhu H, Ren X, Gao L (2022). Two-dimensional MXene supported bismuth for efficient electrocatalytic nitrogen reduction. ChemCatChem.

[229] Guo Y, Wang T, Yang Q, Li X, Li H (2020). Highly efficient electrochemical reduction of nitrogen to ammonia on surface termination modified Ti_3_C_2_T_x_ MXene nanosheets. ACS Nano.

[230] Chatt J, Dilworth JR, Richards RL (1978). Recent advances in the chemistry of nitrogen fixation. Chem. Rev..

[231] Wang T, Li S, He B, Zhu X, Luo Y (2021). Commercial indium-tin oxide glass: a catalyst electrode for efficient N_2_ reduction at ambient conditions. Chin. J. Catal..

[232] Luo Y, Chen GF, Ding L, Chen X, Ding LX (2019). Efficient electrocatalytic N_2_ fixation with MXene under ambient conditions. Joule.

[233] Gao C, Low J, Long R, Kong T, Zhu J (2020). Heterogeneous single-atom photocatalysts: fundamentals and applications. Chem. Rev..

[234] Cheng C, He B, Fan J, Cheng B, Cao S (2021). An inorganic/organic S-scheme heterojunction H_2_-production photocatalyst and its charge transfer mechanism. Adv. Mater..

[235] Mueller-Langer F, Tzimas E, Kaltschmitt M, Peteves S (2007). Techno-economic assessment of hydrogen production processes for the hydrogen economy for the short and medium term. Int. J. Hydro. Energy.

[236] Abdin Z, Zafaranloo A, Rafiee A, Mérida W, Lipiński W (2020). Hydrogen as an energy vector. Renew. Sustain. Energy Rev..

[237] Zhou L, Martirez JMP, Finzel J, Zhang C, Swearer DF (2020). Light-driven methane dry reforming with single atomic site antenna-reactor plasmonic photocatalysts. Nat. Energy.

[238] Malik R, Tomer VK (2021). State-of-the-art review of morphological advancements in graphitic carbon nitride (g-CN) for sustainable hydrogen production. Renew. Sustain. Energy Rev..

[239] Otto IM, Donges JF, Cremades R, Bhowmik A, Hewitt RJ (2020). Social tipping dynamics for stabilizing earth’s climate by 2050. PNAS.

[240] Gong E, Ali S, Hiragond CB, Kim HS, Powar NS (2022). Solar fuels: research and development strategies to accelerate photocatalytic CO_2_ conversion into hydrocarbon fuels. Energy Environ. Sci..

[241] Kandemir T, Schuster ME, Senyshyn A, Behrens M, Schlögl R (2013). The Haber–Bosch process revisited: on the real structure and stability of “ammonia iron” under working conditions. Angew. Chem. Int. Ed..

[242] Pan L, Sun S, Chen Y, Wang P, Wang J (2020). Advances in piezo-phototronic effect enhanced photocatalysis and photoelectrocatalysis. Adv. Energy Mater..

